# Effect of inpatient rehabilitation treatment ingredients on functioning, quality of life, length of stay, discharge destination, and mortality among older adults with unplanned admission: an overview review

**DOI:** 10.1186/s12877-022-03169-2

**Published:** 2022-06-11

**Authors:** K. Lambe, S. Guerra, G. Salazar de Pablo, S. Ayis, I. D. Cameron, N. E. Foster, E. Godfrey, C. L. Gregson, F. C. Martin, C. Sackley, N. Walsh, K. J. Sheehan

**Affiliations:** 1 Department of Population Health Sciences, School of Life Course and Population Sciences, Faculty of Life Sciences & Medicine, Kings College London, London, UK; 2 Early Psychosis: Interventions and Clinical-detection (EPIC) Lab, Department of Psychosis Studies, Institute of Psychiatry, Psychology and Neuroscience, King’s College London, London, UK; 3 John Walsh Centre for Rehabilitation Research, Northern Sydney Local Health District and University of Sydney, Sydney, Australia; 4 Surgical Treatment and Rehabilitation Service (STARS) Education and Research Alliance, The University of Queensland and Metro North Health, Queensland, Australia; 5 Primary Care Centre Versus Arthritis, School of Medicine, Keele University, Keele, UK; 6 Department of Psychology, Institute of Psychiatry, Psychology and Neuroscience, King’s College London, London, UK; 7 Musculoskeletal Research Unit, Translation Health Sciences, Bristol Medical School, University of Bristol, Bristol, UK; 8 Centre for Health and Clinical Research, University of the West of England Bristol, Bristol, UK

**Keywords:** Physiotherapy, Exercise, Geriatrics, Acute care, Hospital, Trauma, Injury, Illness

## Abstract

**Background:**

To synthesise the evidence for the effectiveness of inpatient rehabilitation treatment ingredients (versus any comparison) on functioning, quality of life, length of stay, discharge destination, and mortality among older adults with an unplanned hospital admission.

**Methods:**

A systematic search of Cochrane Library, MEDLINE, Embase, PsychInfo, PEDro, BASE, and OpenGrey for published and unpublished systematic reviews of inpatient rehabilitation interventions for older adults following an unplanned admission to hospital from database inception to December 2020. Duplicate screening for eligibility, quality assessment, and data extraction including extraction of treatment components and their respective ingredients employing the Treatment Theory framework. Random effects meta-analyses were completed overall and by treatment ingredient. Statistical heterogeneity was assessed with the inconsistency-value (I^2^).

**Results:**

Systematic reviews (*n* = 12) of moderate to low quality, including 44 non-overlapping relevant RCTs were included. When incorporated in a rehabilitation intervention, there was a large effect of *endurance exercise, early intervention* and *shaping knowledge* on walking endurance after the inpatient stay versus comparison. *Early intervention*, *repeated practice activities*, *goals and planning*, *increased medical care* and/or *discharge planning* increased the likelihood of discharge home versus comparison. The evidence for activities of daily living (ADL) was conflicting. Rehabilitation interventions were not effective for functional mobility, strength, or quality of life, or reduce length of stay or mortality. Therefore, we did not explore the potential role of treatment ingredients for these outcomes.

**Conclusion:**

Benefits observed were often for subgroups of the older adult population e.g., *endurance exercise* was effective for endurance in older adults with chronic obstructive pulmonary disease, and *early intervention* was effective for endurance for those with hip fracture. Future research should determine whether the effectiveness of these treatment ingredients observed in subgroups, are generalisable to older adults more broadly. There is a need for more transparent reporting of intervention components and ingredients according to established frameworks to enable future synthesis and/or replication.

**Trial registration:**

PROSPERO Registration CRD42018114323.

**Supplementary Information:**

The online version contains supplementary material available at 10.1186/s12877-022-03169-2.

## Introduction

The world’s population is ageing, reflecting advances in economic and social development, public health, sanitation, and medicine [1]. Although people are living longer, multiple chronic and complex health issues increase with age [2]. This demographic trend, the changing health patterns of multimorbidity in old age contribute to fluctuating health service use and associated increased costs [3, 4]. A consequent increase in unplanned hospital admissions for older adults has the potential to lead to hospital associated deconditioning [5], with slower and poorer recovery without appropriate rehabilitation [6].

**Fig. 1:**
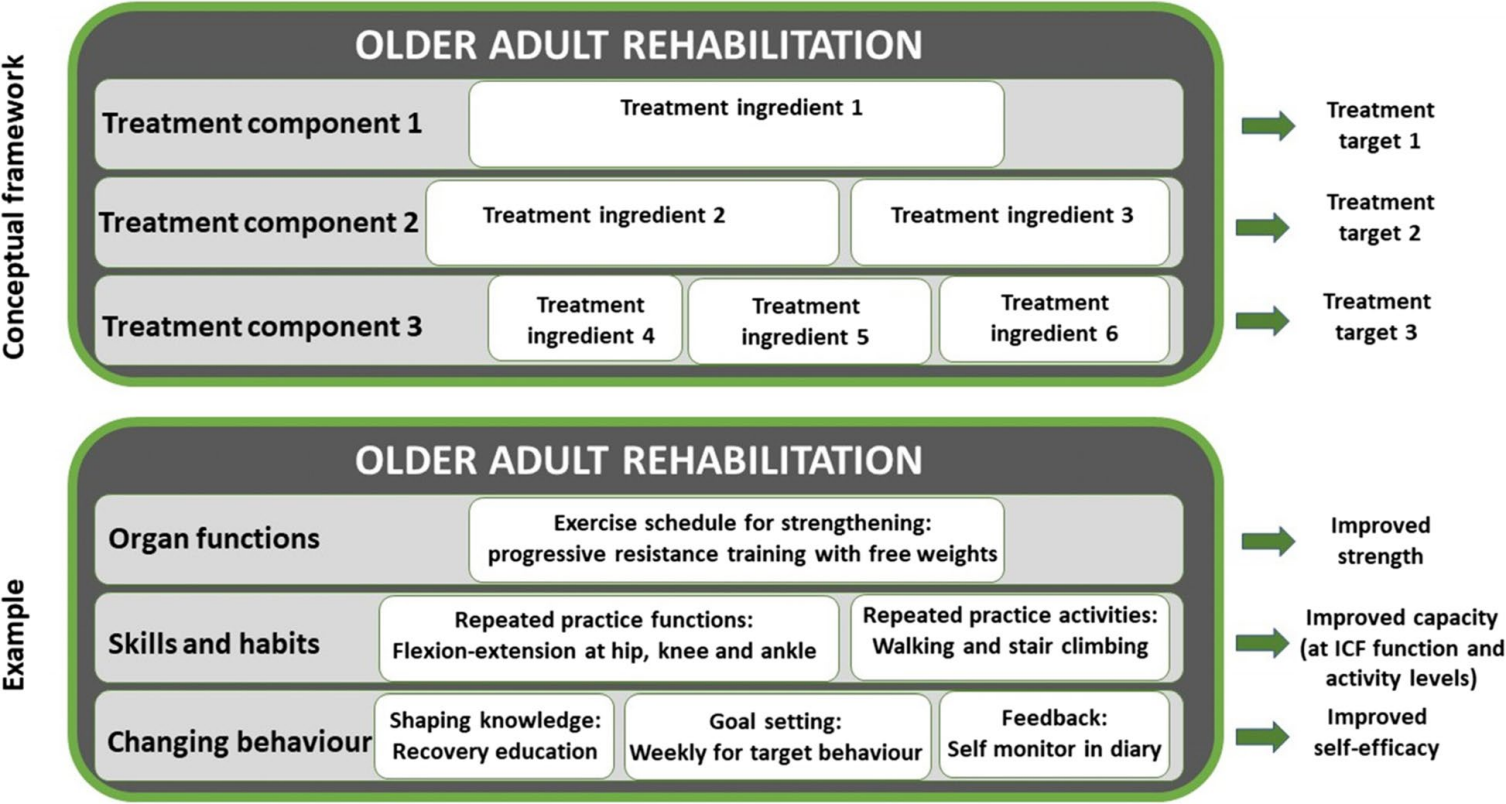
Rehabilitation as a complex intervention made up of treatment components addressing different targets; each treatment component is made up of more specific and measurable treatment ingredients [12]. ICF: International Classification of Functioning

Rehabilitation is defined as a “*set of measures aimed at individuals who have experienced or are likely to experience disability to assist them in achieving and maintaining optimal functioning* (all body functions, activities and participation [7]) *when interacting with their environments.*” [8]. Treatment theory “*refers to a class of specific theories that specify mechanisms by which ingredients of a treatment produce change in the treatment target, the aspect of function that is directly impacted by the treatment”* [9–12]. Treatment theory conceptualises rehabilitation as a complex intervention made up of treatment components which address different targets; each treatment component e.g.*,* skills and habits, is made up of more specific and measurable treatment ingredients, e.g.*,* strength exercises or repeated practice activities (Fig. 1) [9–12]. Healthcare policies are shifting care away from the inpatient setting and into the community – either home or facility [13]. Inpatient rehabilitation may reduce the impact and complications of various health conditions and facilitate the earlier restoration of function, maximising potential for discharge home (and not to a facility) [14]. It is therefore essential to maximise the potential benefits from rehabilitation offered in this setting.

There is a plethora of studies evidencing the effectiveness of inpatient rehabilitation for older adults admitted to hospital with an unplanned episode of injury or illness, summarised in systematic reviews and meta-analyses [15–17]. This rehabilitation often includes multiple treatment ingredients with uncertainty over which ingredient(s) account for the reported change in outcome [12]. This poses a challenge for clinicians when justifying the inclusion of a given ingredient in practice, and for researchers when determining which ingredient(s) to include in future studies of rehabilitation interventions [18].

It would be of value to both clinicians and researchers to determine which treatment ingredient(s) contribute to the effectiveness of rehabilitation [19]. We proposed to address this evidence gap through application of Treatment Theory in an overview review of rehabilitation treatment ingredients for older adults with unplanned hospital admission.

## Aims and objectives

The aims of this overview review were to inform evidence-based inpatient rehabilitation for older adults following an unplanned hospital admission, and to identify gaps in the evidence to inform future research. More specifically, the primary objective was to synthesise the evidence for the effectiveness of inpatient rehabilitation treatment ingredients (versus any comparison) on functioning (body functions, activities) among older adults with an unplanned hospital admission. Secondary objectives included synthesizing the evidence for additional outcomes of quality of life, length of stay, discharge destination, and mortality.

## Methods

We registered the protocol on the international prospective register of systematic reviews (PROSPERO: CRD42018114323). We reported this review in adherence to the Preferred Reporting Items for Systematic Reviews and Meta-Analysis (PRISMA) statement [20]. We did not require ethical approval as it used data from published systematic reviews and meta-analyses.

### Eligibility criteria

Eligibility criteria are outlined in Table 1. Briefly, we included systematic reviews and meta-analyses of randomised controlled trials (RCTs) which compared the effectiveness of inpatient rehabilitation [21] to any comparator group on functioning (body functions, activities), quality of life, discharge destination, length of stay, and/or mortality after inpatient rehabilitation (and where available longest follow-up to one-year) among older adults with an unplanned hospital admission (Table 1). We applied no publication date, language, or geographical limits. We excluded reviews focusing exclusively on older adults post-stroke to avoid conclusions being dominated by the larger evidence base post-stroke.

**Table 1:** Eligibility criteria of systematic reviews and meta-analyses in overview review

	Include	Exclude
**Population**	Reviews of adults with unplanned admission (urgent/emergency) to acute hospital care for any diagnosis other than stroke. Explicitly targeted RCTs of ‘older adults’ as described in eligibility criteria, or which included a subgroup analysis for older adults. Where age was not specified in the review eligibility, we selected relevant RCTs from within reviews which had a median/mean age of at least 65 years.	Reviews of planned admission to acute care. Explicitly targeted RCTs of children, young- or middle-aged adults, adults with stroke, and/or without explicit target of ‘older adults’, and no subgroup analysis for older adults. Where age was not specified in the review eligibility, we excluded RCTs from within reviews which had a median/mean age of less than 65 years.
**Intervention**^**a**^	*All reviews of rehabilitation provided/prescribed by rehabilitation professionals:* • which include exercise • to enable people with disabilities to attain or maintain maximum functioning at the level of body function, activity, and/or participation ^a^ • to prevent immobility related secondary health conditions or complications arising from a primary health condition	*All reviews of:* • rehabilitation involving prevention of first-time health conditions • acute medical management/chronic health condition management unless a goal is explicitly to address functioning (e.g. pulmonary rehabilitation for chronic lung disease with the goal of improving functioning) • rehabilitation directed at improving mental health ^a^ • rehabilitation not within the scope of the practice of rehabilitation professionals (e.g. homeopathy, invasive procedures for deep brain stimulation, hyperbaric oxygen therapy) • rehabilitation not specific to functioning (e.g. targeting a reduction in nonattendance rates) • first aid, pharmacological (including nutritional), paramedic, emergency, and surgical care ^a^
**Comparison**	• Usual care • Placebo • Sham rehabilitation • Alternative rehabilitation	• No comparison
**Outcome**	Validated measure of functioning^b^, and/or quality of life. Measure of length of stay, discharge destination, or mortality. All measured at intervention end with or without follow up (up to 1 year).	Not (validated) measure of functioning^b^ or quality of life and no measure of length of stay, discharge destination, or mortality. Absence of measure at intervention end.
**Time**	Rehabilitation endpoint of discharge from inpatient care.	Rehabilitation endpoint after discharge from inpatient care.
**Study design**	Systematic review and/or meta-analysis where at least 1 of the included primary studies are randomized controlled trials.	Not systematic review, primary research. Systematic review where no primary studies are randomized controlled trials.
**Other**	• Human • Any geographical region • Any language • Any publication dates	• Non-human

^a^ Modified Cochrane Rehabilitation’s criteria for identifying reviews as relevant to rehabilitation [21]. ^b^ categorised by the domains body functions and/or activities (capacity) as specified by the World Health Organisations International Classification of Functioning [7].

### Search methods

We developed structured search strategies, in consultation with a librarian using thesaurus terms for intervention, setting and study design for each database (e.g., EMTREE for EMBASE, MeSH for MEDLINE) and free text, targeting the “title” and “abstract” fields (Supplementary File 1). We searched from inception to December 10th 2020 for published and unpublished systematic reviews in the following electronic databases: Cochrane Library, MEDLINE, Embase, PsychInfo, PEDro, BASE, and OpenGrey. We also screened reference lists of eligible systematic reviews for additional reviews not identified through our search strategies. We exported references to Covidence for deduplication, screening, selection, and quality appraisal [22].

### Screening and selection

We screened titles and abstracts and potentially eligible full text reviews in duplicate against eligibility criteria (KL, CK, SG, KS). A third researcher resolved any discrepancies. We quantified inter-rater reliability using Cohen’s Kappa statistic [23]. We avoided double-counting outcome data in our overview by primary RCT overlap with the creation of a citation matrix ordered first by publication date and then by lead author surname and excluded eligible reviews with no unique RCTs (retaining the most recent reviews) [24].

### Quality appraisal

We assessed the methodological quality of each included review in duplicate using the Assessment of Multiple Systematic Reviews (AMSTAR 2) tool (KL, SH, SG, KS) [25]. AMSTAR 2 is a 16-item checklist which informs an overall qualitative rating on the confidence in the results of a review, based on weaknesses in critical domains [25]. Such domains include whether a protocol was registered, adequacy of literature search, exclusion criteria, and risk of bias. Four options were available when rating, ranging from critically low confidence to high confidence. A third researcher resolved any discrepancies.

### Data extraction

We extracted data onto Microsoft Excel table templates defined a priori in duplicate (KL, EE, CK, SG, KS). A third researcher resolved any discrepancies. We contacted authors to supplement missing or incomplete data.

We extracted the following data items for the systematic reviews: review author, review year, population, intervention, comparators, outcome, number of studies eligible for the current overview, number of patients from eligibility studies. We extracted the following data items for eligible RCTs within the systematic reviews: RCT author, RCT year, country, sample size (intervention and comparator), characteristics of the population where available -age, gender, target group, and preadmission residence, comparison/s, interventions, outcomes and follow-up relevant to the current overview. For the interventions, we extracted three main treatment components and their more specific and measurable treatment ingredients specified by Treatment Theory [9–12]. Component 1: *Organ Functions* (example more specific treatment ingredient: strengthening exercise) [12]; Component 2: *Skills and Habits* (example more specific treatment ingredient: repeated practice of activities +/− increasing demands) [12]; and Component 3: *Changing Behaviour* (example more specific treatment ingredients: goals and planning, shaping knowledge) [26]. Where treatment ingredients did not fall under these three treatment components (e.g.*,* increased medical care), we extracted them under *Other Components*. All treatment ingredients cited were assigned to a component in this review. For our outcomes, we extracted mean and standard deviation in each treatment arm for continuous outcome measures and proportions for categorical outcomes after inpatient rehabilitation and on longest follow-up (up to 1 year). We contacted all authors who presented data as medians, ranges, or 95% confidence intervals for means and standard deviations. If no response was received, we converted data presented as medians and ranges to means and standard deviations using methods as described by Hozo et al. [27]. We converted data presented as 95% confidence intervals to standard errors [28] and subsequently standard deviations (standard deviation = standard error x √sample size).

### Data synthesis

All systematic reviews met the eligibility criteria for inclusion; however, 1) not all RCTs within reviews were relevant, and 2) there was considerable primary RCT overlap between reviews. Therefore, we re-analysed the data by performing random-effects meta-analyses within the subgroup of relevant RCTs for each outcome across the systematic reviews [29]. We estimated Hedges’ g or mean differences for continuous outcomes and log odds ratios for categorical outcomes. We interpreted effect sizes of 0.2 as small, 0.5 moderate, and 0.8 as large [28]. We completed sensitivity analyses with RCTs from reviews of low or critically low quality removed from the analyses.

We stratified meta-analyses which indicated the effectiveness of interventions on outcomes by individual treatment ingredients, e.g. *endurance exercise* [21]. For meta-analyses with at least ten RCTs, small study sample bias was assessed using Egger’s test for continuous outcomes and Peters test for categorical outcomes [28]. We assessed the potential for heterogeneity using I^2^ and followed the Cochrane convention of 0–40% heterogeneity as may not be important, 30–60% as moderate, 50–90% as substantial, and 75–100% as considerable heterogeneity [28]. Where at least ten RCTs were included in the meta-analysis, we also explored the potential for heterogeneity due to differences in characteristics of the RCTs (mean age, target group, continent of publication, and year of publication) with random-effects meta-regression [28] and stratified meta-analysis where there was a plausible characteristic which may explain the heterogeneity e.g., RCT geography on length of stay due to different organisation of care. All analyses were completed in Stata v16 [30]. We summarised RCT findings descriptively where meta-analysis was not possible.

## Results

### Selection

**Fig. 2:**
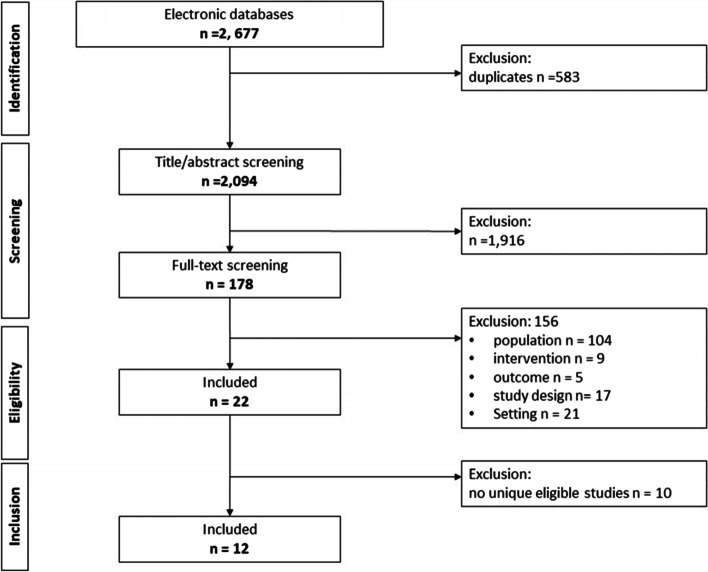
PRISMA Flow Diagram

We included 12 systematic reviews in this overview review. Initial searches identified 2677 systematic reviews, of which 583 were duplicates. On the title and abstract screening, a further 1916 were excluded. Of the 178 reviews assessed at full text screening, 155 were ineligible for the following reasons: population (*n* = 104), intervention (*n* = 9), outcome (*n* = 5), study design (*n* = 17), setting (*n* = 21). Cohen’s Kappa statistic following full text review was 0.73 indicating substantial agreement between assessors. Following generation of a citation matrix ordered by publication date, we excluded a further 10 reviews [16, 31–39] which contained no RCTs not already included in a more recent review (Supplementary File 2) Fig. 2.

### Quality

The results of the quality assessment are presented in Table 2. Overall, seven systematic reviews were assigned a moderate rating for overall confidence in review results (more than one non-critical weakness but no critical flaws) [15, 40, 42, 45, 47–49], four a low rating (one critical flaw – study selection not in duplication or failure to consider risk of bias for interpretation) [17, 41, 43, 46], and one a critically low rating (more than one critical flaw) [44]. Almost all included systematic reviews met the requirements for defining an appropriate research question (*n* = 12) [15, 17, 40–49], search strategy (*n* = 11) [15, 17, 40–43, 45–49], study selection (*n* = 11) [15, 17, 40–45, 47–49], risk of bias assessment (*n* = 11) [15, 17, 40–43, 45–49], explanation of heterogeneity in analyses (*n* = 9) [15, 17, 40–42, 45, 46, 48, 49], and declaring sources of conflicts of interest (*n* = 12) [15, 17, 40–49]. Most systematic reviews failed to explain their selection of the study designs for inclusion (*n* = 11) [15, 17, 40–42, 44–49], declare sources of funding for studies included in the review (*n* = 11) [15, 40–49] and/or carry out an adequate investigation of potential publication bias (*n* = 5) [40, 42, 43, 46, 47].

**Table 2:** Quality assessment of systematic reviews and meta-analyses included in this overview review using AMSTAR 2

AMSTAR 2 DOMAIN																	
Author, Year (Reference)	1	2	3	4	5	6	7	8	9	10	11	12	13	14	15	16	AMSTAR 2 Rating
**Bachmann 2010** [15]	Y	N	N	PY	Y	N	N	Y	PY	N	Y	Y	Y	Y	Y	Y	Moderate
**De Morton, 2007** [17]	Y	N	N	Y	Y	Y	Y	PY	Y	Y	Y	N	N	Y	Y	Y	Low
**Handoll, 2011** [40]	Y	Y	N	PY	Y	Y	Y	Y	Y	N	NMA	NMA	Y	Y	N	Y	Moderate
**Heldmann, 2019** [41]	Y	PY	N	PY	Y	Y	N	Y	Y	N	NMA	NMA	N	Y	NMA	Y	Low
**Machado, 2020** [42]	Y	PY	N	PY	Y	N	N	PY	Y	N	Y	N	Y	Y	N	Y	Moderate
**Martinez-Velilla, 2016** [43]	Y	N	Y	PY	Y	N	N	Y	PY	N	NMA	NMA	N	N	N	Y	Low
**Peck 2020** [44]	Y	N	N	N	Y	N	N	Y	N	N	NMA	NMA	N	N	NMA	Y	Critically low
**Peiris 2018** [45]	Y	Y	N	PY	Y	Y	N	Y	Y	N	Y	Y	Y	Y	Y	Y	Moderate
**Scrivener, 2015** [46]	Y	Y	N	PY	N	Y	N	Y	Y	N	Y	Y	Y	Y	N	Y	Low
**Smith, 2020a** [47]	Y	Y	N	Y	Y	Y	Y	Y	Y	Y	Y	N	Y	N	N	Y	Moderate
**Smith 2020b** [48]	Y	Y	N	PY	Y	Y	N	Y	Y	N	Y	N	Y	Y	Y	Y	Moderate
**Yasmeen 2020** [49]	Y	Y	N	Y	Y	Y	N	Y	Y	N	NMA	NMA	Y	Y	NMA	Y	Moderate

*Abbreviations: *AMSTAR 2: *Y* meets the requirement, *PY *partial yes, *N* = does not meet the requirement, *NMA *no meta-analysis conducted, *NSRI *Only includes non-randomised studies of interventions, *RCT* Only includes RCTs

AMSTAR 2 DOMAINS: 1. PICO - “Did the research questions and inclusion criteria for the review include the components of PICO? 2. Protocol – “Did the report of the review contain an explicit statement that the review methods was established prior to the conduct of the review, and did the report justify any significant deviations from the protocol? 3. Study design – Did the review authors explain their selection of the study designs for inclusion in the review? 4. Search strategy – Did the review authors use a comprehensive literature search strategy? 5. Study selection – Did the review authors perform study selection in duplicate? 6. Data extraction – Did the review authors perform data extraction in duplicate? 7. Excluded studies – Did the review authors provide a list of excluded studies and justify the exclusions? 8. Included studies – Did the review authors describe the included studies in adequate detail? 9. Risk of bias – Did the review authors use a satisfactory technique for assessing the risk of bias (RoB) in individual studies that were included in the review? 10. Funding sources – Did the review authors report on the sources of funding for the studies included in the review? 11. Meta-analysis – If a meta-analysis was justified did the review authors use appropriate methods for statistical combination of results? 12. Impact risk of bias – If meta-analysis was performed did the review authors assess the potential impact of RoB in individual studies on the results of the meta-analysis or other evidence synthesis? 13. Discussing risk of bias – Did the review authors account for RoB in individual studies when interpreting/discussing the results of the review? 14. Heterogeneity – Did the review authors provide a satisfactory explanation for, and discussion of, any heterogeneity observed in the results of the review? 15. Publication bias – If they performed quantitative synthesis did the review authors carry out an adequate investigation of publication bias (small study bias) and discuss its likely impact on results of the review? 16. Conflicts of interest – Did the review authors report any potential sources of conflict of interest, including any funding they received for conducting the review?

### Characteristics

The 12 systematic reviews included 41 unique RCTs and 10,444 older adults with an unplanned hospital admission relevant to this overview (mean (min - max) sample size per RCT: 261 (12–1531)) (Table 3). The target population of systematic reviews included older adults admitted for a general medical reason (*n* = 5) [15, 17, 43, 45, 48], for any unplanned reason (*n* = 3) [41, 46, 49], with hip fracture (*n* = 2) [40, 47], orthopaedic trauma (*n* = 1) [44], or an exacerbation of chronic obstructive pulmonary disease (COPD) (*n* = 1) [42]. Outcomes captured by the systematic reviews included functional mobility, ADLs, walking endurance, walking speed, and/or lower limb strength (*n* = 11) [15, 17, 40–46, 48, 49]; quality of life (*n* = 4) [40, 42, 48, 49]; length of stay (*n* = 9) [15, 17, 40–42, 45–48]; discharge destination (*n* = 2) [41, 47]; and mortality (*n* = 7) [15, 17, 40, 41, 43, 48, 49].

**Table 3:** Characteristics of reviews included in overview review

Author, year	Population	Intervention	Comparator	Outcomes	Studies eligible for current overview n (%)	Number of patients (*n* = 10,444) ^a^
**Bachmann, 2010** [15]	Medical admission	Inpatient rehabilitation specifically designed for geriatric patients, including multidisciplinary and accelerated rehabilitation programmes	Usual care	Function Length of stay Mortality	1 (5.88)	71
**De Morton, 2007** [17]	Medical admission	Exercise or multidisciplinary program with exercise	Usual care or no treatment	Function Length of stay Mortality	1 (11.1)	237
**Handoll, 2011** [40]	Hip fracture	Post-operative mobilisation strategies such as weight bearing, exercises, physical training and muscle stimulation, and mobilisation and nutrition	Any comparator	Function Length of stay Quality of life Mortality	5 (26.3)	568
**Heldmann, 2019** [41]	Hip fracture Medical admission Abdominal surgery	Exercise or multidisciplinary program with exercise	Any comparator	Function Length of stay Discharge destination Mortality	15 (62.5)	4941
**Machado, 2020** [42]	COPD	Pulmonary rehabilitation, exercise training, breathing techniques, airway clearance techniques and/or education and psychosocial support	Usual care of any component of pulmonary rehabilitation	Function Length of stay Quality of life	12 (28.6)	716
**Martinez-Velilla, 2016** [43]	Medical admission	Exercise and early rehabilitation (physical therapy, occupational therapy, and physical activity as soon as physiological stable)	Any comparator	Function Mortality	3 (17.7)	325
**Peck, 2020** [44]	Orthopaedic trauma	Mobilisation, defined as any form of activity or exercise, within the first 24 hours of admission	Any comparator	Function	1 (12.5)	89
**Peiris, 2018** [45]	Medical admission	Additional physical therapy (extra and/or longer sessions) supervised by physical therapists or physical therapy assistants	Usual care	Function Length of stay	1 (4.16)	996
**Scrivener, 2015** [46]	Hospital admission	After-hours or weekend rehabilitation in any form (e.g., arm exercise, mobility training) and could be unsupervised (i.e., self-monitored programs) or supervised (e.g., therapists, families, assistants, nursing staff)	Any comparator	Function Length of stay	1 (14.3)	47
**Smith, 2020a** [47]	Hip fracture with/ without dementia	New models of care e.g., protocols for interdisciplinary working and/or discharge planning, enhanced complications monitoring, intensive rehabilitation, extension of rehabilitation into community after discharge, enhanced rehabilitation for persons with dementia	Usual care	Length of stay Discharge destination	1 (14.3)	12
**Smith, 2020b** [48]	Medical admission	Mobilisation programmes to increase ward-based physical activity, with education for carers and patients, change in healthcare practice (e.g. enhanced rehabilitation, staff allocation and time, earlier assessments of barriers) and/or environmental changes	Any comparator	Function Length of stay Mortality Quality of life	4 (57.1)	2308
**Yasmeen, 2020** [49]	Hospital admission	Caregiver-mediated interventions to improve mobility or ADL, by providing education, training, preparation for discharge, and/ or collaborating with providers	Any comparator	Function Mortality Quality of life	1 (2.50)	134

*COPD* chronic obstructive pulmonary disease ^a^ Number assigned from studies relevant to the current overview

Characteristics (as well as their treatment components and more specific and measurable ingredients) of the 44 RCTs included in the 12 systematic reviews are detailed in Supplementary Files 3 and 4. Examples of each treatment ingredient are specified in Table 4. For component 1 Organ Functions, treatment ingredients included: endurance exercise (*n* = 13), strengthening (*n* = 12), energy applied to soft tissue (*n* = 7), and/or breathing related exercises/training (*n* = 6). For component 2 Skills and Habits, treatment ingredients included: repeated practice activities (*n* = 15), functions (*n* = 8), and/or ‘exercise rehabilitation’ (*n* = 6). For component 3 Changing Behaviour, treatment ingredients included: shaping knowledge (*n* = 16), feedback and monitoring (*n* = 14), goals and planning (*n* = 11), antecedents (*n* = 12), natural consequences (*n* = 5), social support (*n* = 2), and/or comparison of behaviour (*n* = 1). For Other Components, treatment ingredients included: increased medical care for e.g., avoidance of complications and/or pain management (*n* = 14), early intervention (*n* = 12), team meetings and care planning (n = 11), discharge planning (*n* = 9), nutritional intervention (*n* = 8), home visits during inpatient stay (*n* = 5), and/or cognitive orientation exercise (*n* = 2).

**Table 4:** Examples of treatment ingredients identified from RCTs included in systematic reviews of inpatient rehabilitation for older adults with unplanned admission to hospital

Treatment Component	Treatment ingredient	Examples
**Organ functions**	Strengthening exercise	Quadriceps strengthening, leg extensor strengthening, progressive resistance training with weights, elastic bands, and/or body weight, calisthenics, sit to stand or stair training.
	Endurance exercise	Treadmill training, pedal/cycle ergometer, walking programme.
	Energy applied to soft tissues	Neuromuscular electrical stimulation, vibrating platforms.
	Breathing related exercise/training	Deep breathing, relaxation techniques, pursed lip breathing.
**Skills and habits**	Repeated practice functions	Active range of motion exercises for the upper and lower limb in lying, sitting, or standing.
	Repeated practice activities	ADL training (mobility in bed, sitting and standing, chair to bed transfers, wheelchair to bed/toilet transfers, dressing, bathing, personal hygiene, toilet use), transfer practice.
	Repeated exercise rehabilitation	Exercise rehabilitation at an increased frequency.
**Changing behaviour**	Goals and planning	Action planning, goal setting for target behaviour or target outcome.
	Feedback and monitoring	Monitoring outcomes of behaviour without feedback to the participant, self-monitoring through diary entries, feedback during behaviour with modifications as needed e.g., reduce repetitions.
	Social support	Group sessions with other patients, sessions with patients and their carers to build confidence in ADL, assistance at mealtimes.
	Shaping knowledge	Instructions on how to perform a behaviour in person / with leaflet.
	Natural consequences	Information on condition/injury delivered in person with visual aid e.g., leaflet /Xray.
	Comparison of behaviour	Demonstration of an exercise/use of equipment.
	Antecedents	Restructuring the physical environment e.g., removal of clutter from hallways. Assessment and intervention on social environment. Adding objects to the environment e.g., mobility aids, provision of clocks and calendars.
**Other intervention components**	Cognitive orientation exercise	Set of questions asked regularly to improve orientation -day, month, year, date, ward, bed number, nurse name.
	Team meetings and care planning	Multidisciplinary team meetings of increased frequency for planning.
	Discharge planning	Early discharge planning with multidisciplinary team.
	Increased medical care	Increased monitoring of pain, provision of oxygen enriched air, increased monitoring for potential complications e.g., pressure ulcers.
	Nutritional intervention	Protein-enriched meals, nutritional supplements, assistance at mealtimes.
	Early intervention	Early mobilisation (often on day of or after surgery), early start of rehabilitation, early discharge planning, early geriatrician review
	Home visit	Pre-discharge home visit by physiotherapy or occupational therapy

*ADL* activities of daily living

The comparator was usual care for the majority of RCTs (*n* = 42, 95.5%) identified from the systematic reviews. Physiotherapy/occupational therapy was a core component of usual care for 21 RCTs (50.0%), provided following a physician referral for 5 RCTs (11.9%), not a component of usual care for 13 RCTs (30.9%), or not specified for 1 RCT (2.4%). Two RCTs (4.8%) included education and usual care (1 RCT with physiotherapy/occupational therapy, 1 RCT usual care not specified) as the comparator. The comparator was an alternative intervention - delayed ambulation or delayed weight bearing for 2 RCTs (4.6%).

### Synthesis

Meta-analyses were completed for function (functional mobility, ADL, walking speed, walking endurance, lower limb strength), health-related quality of life, length of stay, discharge destination, and mortality (Table 5). Details for population, intervention treatment ingredients, comparator, outcome measurement, and follow-up for each RCT included in each meta-analysis are available alongside forest plots in Supplementary File 5. We noted no difference in effect estimates or confidence intervals for sensitivity analyses which excluded RCTs from reviews of low or critically low quality. If interventions favored the control group, this is specified in text alongside the results of meta-analyses. Forest plots for meta-analyses by treatment ingredient are available in Supplementary File 6. Meta-regression was used to explore heterogeneity in analyses for ADL, discharge home and length of stay. Outcomes which could not be included in meta-analyses due to absence of measure of central tendency or dispersion, sole study, and/or multiple measures for the same outcome are summarised in text and in Supplementary File 7.

**Table 5:** Meta-analyses of the effectiveness of inpatient rehabilitation on function, quality of life, length of stay, discharge destination and mortality, versus comparison, among older adults with unplanned admission to acute care, overall and by treatment ingredient

	n studies	n total (intervention)	n total (comparison)	Effect size^a^ (95% CI)	Z Score	p	Test for Heterogeneity			Test for small study sample bias†
							Q	I^**2**^	p	p
**Functional mobility after inpatient rehabilitation**										
**overall**	**5**	**761**	**733**	**0.10 (−0.04, 0.24)**	**1.38**	**0.17**	**4.36**	**22.30**	**0.36**	**–**
**Functional mobility at longest follow-up (4–12 months)**										
**overall**	**2**	**159**	**141**	**0.30 (− 0.26, 0.86)**	**1.05**	**0.29**	**3.15**	**68.22**	**0.08**	**–**
**ADL after inpatient rehabilitation**										
**overall**	**15**	**1992**	**1937**	**0.21 (0.00, 0.42)**	**2.00**	**0.04**	**54.12**	**86.58**	**< 0.01**	**0.01**
endurance exercise	4	109	116	0.51 (− 0.34, 1.36)	1.18	0.24	27.68	89.43	< 0.01	–
strengthening exercise	3	71	65	0.30 (−0.05, 0.64)	1.69	0.09	2.06	5.57	0.36	–
energy applied to soft tissue	3	58	56	0.95 (0.23, 1.66)	2.60	0.01	6.51	70.20	0.04	–
repeated practice activities	7	1274	1246	−0.02 (− 0.13, 0.10)	− 0.27	0.78	7.67	30.29	0.26	–
repeated exercise rehabilitation	3	545	542	0.42 (− 0.04, 0.87)	1.80	0.07	7.36	69.94	0.03	–
goals and planning	9	643	613	0.22 (−0.17, 0.61)	1.12	0.26	42.16	90.45	< 0.01	–
feedback and monitoring	7	266	262	0.33 (−0.19, 0.84)	1.24	0.21	36.36	88.02	< 0.01	–
shaping knowledge	4	362	360	−0.13 (− 0.27, 0.02)	−1.73	0.08	3.01	0.00	0.39	–
antecedents	2	762	732	−0.08 (− 0.44, 0.28)	−0.46	0.65	3.04	67.16	0.08	–
increased medical care	4	971	932	0.10 (−0.23, 0.43)	0.60	0.55	9.79	85.43	0.02	–
nutritional intervention	2	891	862	0.06 (−0.03, 0.15)	1.25	0.21	0.09	0.00	0.76	–
early intervention	2	215	200	0.35 (−0.23, 0.93)	1.17	0.24	4.86	79.43	0.03	–
**ADL at longest follow-up (1–12 months)**										
**overall**	**5**	**649**	**246**	**0.04 (−0.31, 0.38)**	**0.21**	**0.83**	**16.44**	**82.69**	**< 0.01**	**–**
**Improved ADL after inpatient rehabilitation (categorical)**										
**overall**	**6**	**1445**	**1334**	**0.21 (− 0.07, 0.49)**	**1.49**	**0.14**	**17.85**	**71.46**	**< 0.01**	**–**
**Improved ADL at longest follow-up (categorical) (12 months)**										
**overall**	**2**	**333**	**293**	**0.45 (−0.05, 0.96)**	**1.78**	**0.08**	**2.10**	**52.36**	**0.15**	**–**
**Walking speed after inpatient rehabilitation**										
**overall**	**5**	**588**	**587**	**0.17 (0.05, 0.28)**	**2.85**	**< 0.01**	**6.16**	**0.00**	**0.19**	**–**
strengthening exercise	2	59	56	−0.03 (−0.39, 0.34)	−0.14	0.89	0.09	0.00	0.76	–
repeated exercise rehabilitation	2	509	511	0.53 (−0.34, 1.40)	1.20	0.03	4.80	79.15	0.03	–
**Walking endurance after inpatient rehabilitation**										
**overall**	**6**	**173**	**134**	**1.50 (0.39, 2.60)**	**2.66**	**0.01**	**41.35**	**94.40**	**< 0.01**	**–**
endurance exercise	3	110	71	2.44 (0.49, 4.38)	2.46	0.01	24.76	95.56	< 0.01	–
shaping knowledge	2	95	57	1.51 (0.56, 2.46)	3.11	< 0.01	5.97	83.24	0.01	–
early intervention	2	49	51	0.51 (0.12, 0.91)	2.56	0.01	0.04	0.00	0.85	–
**Walking endurance Pre/post intervention change**										
**overall**	**3**	**69**	**70**	**Log OR: 1.23 (0.68, 1.78)**	**4.36**	**< 0.01**	**4.32**	**54.96**	**0.12**	**–**
endurance exercise	2	54	56	Log OR: 0.98 (0.59, 1.37)	4.94	< 0.01	0.01	0.00	0.92	–
**Lower limb strength after inpatient rehabilitation**										
**overall**	**5**	**130**	**146**	**0.02 (−0.50, 0.55)**	**0.09**	**0.93**	**32.91**	**80.26**	**< 0.01**	**–**
**Health related quality of life after inpatient rehabilitation**										
**overall**	**5**	**795**	**788**	**−0.15 (− 0.37, 0.07)**	**−1.35**	**0.18**	**12.99**	**60.47**	**0.04**	**–**
**Health related quality of life at longest follow-up (12 months)**										
**overall**	**2**	**578**	**572**	**0.01 (−0.11, 0.12)**	**0.12**	**0.91**	**0.03**	**0.00**	**0.85**	–
**Health related quality of life pre/post intervention change**										
**overall**	**2**	**39**	**39**	**Log OR: −0.40 (− 0.84, 0.04)**	**−1.78**	**0.07**	**0.13**	**0.00**	**0.72**	**–**
**Length of stay (standardised mean difference)**										
**overall**	**28**	**3461**	**3510**	**MD: −0.54 (−1.32, 0.23)**	**−1.38**	**0.17**	**233.20**	**88.13**	**< 0.01**	**< 0.001**
**Discharge destination: living at home after inpatient rehabilitation**										
**overall**	**11**	**1914**	**1837**	**Log OR: 0.47 (0.17, 0.76)**	**3.07**	**< 0.01**	**19.03**	**45.95**	**0.04**	**0.22**
repeated practice activities	6	1551	1232	Log OR: 0.49 (0.11, 0.87)	2.50	0.01	13.82	60.41	0.02	–
repeated exercise rehabilitation	2	44	31	Log OR: 0.94 (−0.03, 1.90)	1.91	0.06	0.12	0.00	0.73	–
goals and planning	2	55	25	Log OR: 0.83 (0.21, 1.45)	2.63	0.01	1.20	16.44	0.27	–
antecedents	5	1309	1208	Log OR: 0.20 (−0.25, 0.64)	0.86	0.39	7.99	47.82	0.09	–
increased medical care	8	1768	1683	Log OR: 0.38 (0.04, 0.73)	2.21	0.03	15.47	53.78	0.03	–
early intervention	7	632	647	Log OR: 0.60 (0.20, 1.00)	2.96	< 0.01	8.39	27.45	0.21	–
team meetings and care planning	6	1528	1421	Log OR: 0.42 (−0.04, 0.88)	1.80	0.07	15.46	65.52	0.01	–
discharge planning	6	1656	1580	Log OR: 0.46 (0.09, 0.84)	2.40	0.02	13.59	62.41	0.02	–
nutritional intervention	4	1414	1325	Log OR: 0.32 (−0.27, 0.91)	1.07	0.28	13.96	79.34	< 0.01	–
**Discharge destination: living at home at longest follow-up (3–12 months)**										
**overall**	**2**	**328**	**348**	**Log OR: 0.38 (0.03, 0.74)**	**2.14**	**0.03**	**0.16**	**0.00**	**0.69**	**–**
**Mortality after inpatient rehabilitation**										
**overall**	**12**	**2853**	**2766**	**Log OR: −0.09 (− 0.40, 0.23)**	**−0.55**	**0.58**	**12.62**	**4.24**	**0.32**	**0.09**
**Mortality at longest follow-up (1–12 months)**										
**overall**	**12**	**2108**	**2120**	**Log OR: −0.12 (− 0.28, 0.05)**	**− 1.42**	**0.16**	**8.29**	**0.00**	**0.69**	**0.49**

*Abbreviations: CI* confidence interval, *OR* odds ratio, *MD* mean difference ^a^ Hedges g unless stated otherwise † for meta-analysis with at least 10 randomised controlled trials

### Function

#### Walking endurance

Rehabilitation had a large effect on walking endurance versus comparison after inpatient stay (Total score: 6 RCTs including 307 participants; Hedges’ g = 1.50, 95% CI: 0.39, 2.60. I^2^ = 94.40; Change score: 3 RCTs including 139 participants; Log OR = 1.23, 95% CI: 0.68, 1.78. I^2^ = 54.96) supported by results of RCTs from one systematic review not included in the meta-analysis [42]. When included in a rehabilitation intervention, the treatment ingredients *endurance exercise* (Total score: 3 RCTs including 181 participants; Hedges’ g = 2.44, 95% CI: 0.49, 4.38. I^2^ = 95.56; Change score: 2 RCTs including 110 participants; Log OR = 0.98, 95% CI: 0.59, 1.37. I^2^ = 0.00) and *shaping knowledge* (2 RCTs including 152 participants; Hedges’ g = 1.51, 95% CI: 0.56, 2.46. I^2^ = 83.24) had a large effect, while *early intervention* had a moderate effect (2 RCTs including 100 participants; Hedges’ g = 0.51, 95% CI: 0.12, 0.91. I^2^ = 0.00) on walking endurance versus comparison after inpatient stay.

#### Walking speed

Rehabilitation had a small effect on walking speed versus comparison after inpatient stay (5 RCTs including 1175 participants; Hedges’ g = 0.17, 95% CI: 0.05, 0.28. I^2^ = 0.00). One systematic review reported on one RCT which noted no effect at follow-up [40]. When included in a rehabilitation intervention, the treatment ingredients *strengthening exercise* or *repeated exercise rehabilitation* did not increase walking speed.

#### Activities of daily living

Rehabilitation had a small effect on ADL versus comparison after inpatient stay (15 RCTs including 3929 participants; Hedges’ g = 0.21, 95% CI: 0.00, 0.42. I^2^ = 86.58). The effect was similar but non-significant for ADL change score (6 RCTs including 2779 participants; Log OR = 0.21, 95% CI: − 0.07, 0.49. I^2^ = 71.46). The effect was not sustained at 1–12 month follow-up (Total score: 5 RCTs including 895 participants; Hedges’ g = 0.04, 95% CI: − 0.31, 0.38. I^2^ = 82.69, 1 RCT favoured comparison; Change score: 2 RCTs including 973 participants; Log OR = 0.45, 95% CI: − 0.05, 0.96. I^2^ = 52.36). The absence of an effect was supported by results of RCTs from six systematic reviews not included in the meta-analyses [41–43, 45, 48, 49]. There was evidence of small study sample bias for the analysis of total ADL after inpatient rehabilitation (*p* = 0.01)*.* For estimates of total ADL after inpatient stay, the total effect of rehabilitation interventions adjusted for age, target population, RCT geography, and publication year was not significant (*p* = 0.12) in meta-regression.

When included in a rehabilitation intervention, the treatment ingredient *energy applied to soft tissue* had a large effect versus comparison after inpatient stay (3 RCTs including 114 participants; Hedges’ g = 0.95, 95% CI: 0.23, 1.66. I^2^ = 70.20). There was no effect of *endurance exercise*, *strengthening exercise*, *repeated practice activities*, *repeated exercise rehabilitation*, *goals and planning*, *feedback and monitoring*, *shaping knowledge*, *antecedents*, *increased medical care, nutritional intervention, or early intervention*, on ADL versus comparison.

#### Other measures of function

Rehabilitation did not improve functional mobility or lower limb strength versus comparison after inpatient stay or functional mobility at follow-up evidenced by meta-analysis. Two systematic reviews identified RCTs reporting a between group difference in functional mobility when measured with the Physical Performance and Mobility Examination after inpatient rehabilitation [48] or the Short Physical Performance Battery at follow-up [41].

### Discharge destination

Rehabilitation was effective at increasing the odds of living at home versus comparison after inpatient rehabilitation (11 RCTs including 3751 participants; Log OR = 0.47, 95% CI: 0.17, 0.76. I^2^ = 45.95) and at 3–12-month follow-up (2 RCTs including 676 participants; Log OR = 0.38, 95% CI: 0.03, 0.74. I^2^ = 0.00). When included in a rehabilitation intervention, the treatment ingredients *repeated practice activities* (6 RCTs including 2783 participants; Log OR = 0.49, 95% CI: 0.11, 0.87. I^2^ = 60.41), *goals and planning* (2 RCTs including 80 participants; Log OR = 0.83, 95% CI: 0.21, 1.45. I^2^ = 16.44), *increased medical care* (8 RCTs including 3451 participants; Log OR = 0.38, 95% CI: 0.04, 0.73. I^2^ = 53.78) *early intervention* (7 RCTs including 1279 participants; Log OR = 0.60, 95% CI: 0.20, 1.00. I^2^ = 27.45), and *discharge planning* (6 RCTs including 3236 participants; Log OR = 0.46, 95% CI: 0.09, 0.84. I^2^ = 62.41) increased the odds of living at home versus comparison after inpatient rehabilitation. When included in a rehabilitation intervention, the rehabilitation ingredients *repeated exercise rehabilitation*, *antecedents*, *team meetings and care planning*, and *nutritional intervention* had no effect on the odds of living at home after the period of inpatient rehabilitation. There was no evidence of small study sample bias. For total estimates after inpatient stay, the total effect of age, target population, RCT geography, and publication year was not significant (*p* = 0.14) in meta-regression suggesting these variables do not explain the observed heterogeneity. Subsequent meta-analysis was not carried out.

### Quality of life

Rehabilitation did not increase health-related quality of life versus comparison after inpatient stay (Total score: 5 RCTs including 1583 participants; Hedges’ g = − 0.15, 95% CI: − 0.37, 0.07. I^2^ = 60.47; Change score: 2 RCTs including 78 participants; Log OR = − 0.40, 95% CI: − 0.84, 0.04. I^2^ = 0.00), or on 12-month follow-up (2 RCTs including 1150 participants; Hedges’ g = 0.01, 95% CI: − 0.11, 0.12. I^2^ = 0.00). Three systematic reviews reported on RCTs not incorporated in the meta-analysis which favoured rehabilitation intervention versus comparison after inpatient stay [42, 45] and reported conflicting evidence for follow-up [41, 42, 45].

### Length of stay

Rehabilitation did not reduce the length of stay versus comparison after inpatient stay (29 RCTs including 6971 participants; mean difference = − 0.54, 95% CI: − 1.32, 0.23. I^2^ = 88.13, 3 RCTs favoured comparison); however, evidence was detected for small study sample bias (*p* < 0.001). For estimates of length of stay, the total effect of rehabilitation interventions adjusted for age, target population, RCT geography, and publication year was significant (*p* < 0.001) in meta-regression. A subsequent stratified meta-analysis by RCT geography was conducted. The absence of an effect of rehabilitation on length of stay persisted across regions with substantial heterogeneity for Australia (I^2^ = 86.26) and Europe (I^2^ = 76.47), and heterogeneity which may not be important for the United States of America (I^2^ = 18.10%).

### Mortality

Rehabilitation did not reduce mortality among older adults with unplanned hospital admission versus comparison after inpatient rehabilitation (12 RCTs including 5619 participants; Hedges g = − 0.09, 95% CI: − 0.40, 0.23. I^2^ = 4.24, 1 RCT favoured comparison) or 1–12 month follow-up (13 RCTs including 4366 participants; Hedges’ g = − 0.12, 95% CI: − 0.28, 0.05. I^2^ = 0.00), further supported by an RCT from 1 systematic review not included in the meta-analysis [40]. No evidence was detected of small study sample bias.

## Discussion

### Main findings

We identified 12 systematic reviews of moderate to low quality which included 44 unique RCTs relevant to the current overview. When incorporated in a rehabilitation intervention, we report a large effect of the treatment ingredients *endurance exercise* (exclusively from RCTs of older adults with COPD)*, early intervention* (predominantly from RCTs of older adults after hip fracture) and *shaping knowledge* (exclusively from RCTs of older adults with COPD) on walking endurance after the inpatient stay versus comparison. We also reported beneficial effects of *early intervention*, *repeated practice activities*, *goals and planning*, *increased medical care* and/or *discharge planning* on discharge home. The evidence for effectiveness of treatment ingredients that improve ADL was conflicting. Rehabilitation interventions were not found to be effective for functional mobility, strength, or quality of life, or reduce length of stay or mortality. Therefore, we did not explore the potential role of treatment ingredients for these outcomes.

### Interpretation

Given ceaseless drives to decrease inpatient lengths of stay, it is important for clinicians to preferentially select treatment ingredients most likely to improve outcomes at discharge [13]. However, for effective inpatient rehabilitation interventions, previous systematic reviews highlighted a lack of sufficient data to determine the key features of successful interventions [15, 16]. We sought to supplement the existing evidence by exploring the role of individual treatment ingredients in the overall effectiveness of inpatient rehabilitation. We employed Treatment Theory [9–12] as a framework for the identification of treatment ingredients which may contribute to reported effectiveness. Our analyses identified a select few treatment ingredients for consideration by clinicians.

The treatment ingredient *endurance exercise* had a positive effect on walking endurance. This is important as objective quantitative data indicate adults over the age of 65 years take a median of just 468 steps per day during their inpatient stay (no difference by admitting reason or illness severity) [50]. Given the delay between discharge from the inpatient setting to initiation of community rehabilitation, it is important to optimise walking endurance early in rehabilitation [51, 52]. Three RCTs were included in the analysis of *endurance exercise;* all included patients with COPD exacerbations and these favoured the intervention group. The treatment ingredient was comprised of pedal ergometry daily with increased resistance [53], treadmill training twice daily with increasing duration (from 5 to 20 minutes) [54], or walking five times per day [55]. The largest individual effect sizes were noted for walking five times per day, followed by treadmill training twice daily, and then pedal ergometry (Supplementary File 6). A walking program does not require equipment and could be supported by members of the multidisciplinary team [56, 57] as well as formal and informal carers [49] during the inpatient stay. Where staffing levels are low and a walking programme could not be supported, pedal ergometry offers a low-cost alternative which could be completed at the bedside.

With bedrest, muscle strength is lost rapidly at a rate of 5% per day [5]. We found *early intervention* as a treatment ingredient to be effective at increasing endurance and the likelihood of a home discharge when incorporated into inpatient rehabilitation for older adults after an unplanned hospital admission. This is unsurprising given potential for rehabilitation to mitigate hospital-associated deconditioning [43] and prevent discharge to a higher level of care [58]. Most RCTs focused on older adults undergoing surgery for hip fracture (*n* = 7, 78%) with early intervention defined by mobilisation from bed within the first two days of surgery. This evidence has informed wide acceptance older adults with hip fracture should receive early mobilisation after surgery with early mobilisation a key performance indicator in national audits [59].

A discharge destination of home was more likely among participants who received interventions which incorporated treatment ingredients of *goals and planning*, *repeated practice of activities*, *increased medical care*, and/or *discharge planning* versus comparison. More specific detail for these treatment ingredients was limited. For example, *repeated practice of activities* often reflected ‘ADL training’ with no further detail related to the frequency, duration, or type of activities. One RCT specified transfers were practiced twice daily for 30 minutes [60]. Another indicated ADL training was completed twice daily for five days of the week but did not specify which activities were practiced [61].

### Comparison with other studies

The findings of the current overview are consistent with those of the underlying systematic reviews which conclude that inpatient rehabilitation can improve functioning [15, 43, 48, 49] and the likelihood of discharge to home [15, 17, 48], but has no effect on mortality [17, 48] or length of stay [17, 46, 48] versus comparison (usual care for 95% of RCTs). This current overview does not support previous findings where inpatient rehabilitation led to improvements in quality of life [42, 45], or reductions in length of stay [47] or mortality [15]. This absence of an effect for the current overview may be due to the fact usual care comprised some form of rehabilitation in 29 of the 44 RCTs (2 additional not specified) which may attenuate the estimate of rehabilitation effectiveness between groups.

### Limitations

There are several limitations to this overview review. First, we needed to make two protocol changes a) outcome data were extracted at ‘end of inpatient rehabilitation’, which was a change from our protocol which specified ‘on discharge’ due to lack of clarity in published data, and b) we excluded systematic reviews exclusively addressing post-stroke rehabilitation at full text selection due to their often impairment focus (e.g., upper limb motor deficit) that would not be potentially translatable to other admitting diagnoses. Second, where intervention detail was limited, we termed treatment ingredients such as *repeated exercise rehabilitation* where exercise rehabilitation was mentioned but not detailed, *shaping knowledge* where education was specified but not detailed, or *increased medical care* where examples of what ‘increased care’ may entail were provided but not explicitly measured. This may have led to an underestimation of more specific treatment ingredients. Third, we noted moderate to substantial heterogeneity for several outcomes overall and by treatment ingredient. It was not possible to complete meta-regression across all analyses due to the low number of RCTs [28]. For each analysis, we report the count of RCTs that favoured the comparison to guide the reader in their interpretation of uncertainty due to heterogeneity. Fourth, we attempted to reduce the number of analyses (and risk of multiplicity) by focusing on outcomes which changed following rehabilitation interventions [28]. Nonetheless, there is a risk some of the reported effects may be due to chance alone [28]. Fifth, we stratified meta-analyses by treatment ingredient to explore which treatment ingredients may be more or less effective. We were not able to determine whether potentially ineffective treatment ingredients become effective when combined with other treatment ingredients [10]. Sixth, we defined ‘functioning’ by body functions and activities and did not evaluate the effect of treatment ingredients on participation as an aspect of functioning [7]. Finally, an overview review only reports on data that have been published, systematically reviewed and/or meta-analysed and includes limitations of included RCTs [62].

### Implications for clinical practice and research

The effect of *endurance exercise* on endurance was reflective of three RCTs of older adults with an unplanned admission due to an exacerbation of COPD while the findings from *early intervention* predominantly reflected older adults with hip fracture. These treatment ingredients should be prioritised for implementation for these patient groups. It may be reasonable to generalise the recommendations to similar groups of older adults with an unplanned admission to hospital. For example, *early intervention* may be generalised to other non-hip fragility fractures [63], and *endurance exercise* to patients admitted with exacerbations of other chronic lung diseases [64]. Whether the recommendations may be generalised to less similar groups require more consideration. For example, in the current overview no systematic reviews included RCTs explicitly focusing on older adults with heart failure. This is likely as most cardiac rehabilitation spans both hospital and community settings (and therefore would be excluded from the current overview). *Endurance exercise* is a key component of most cardiac rehabilitation programmes offered to older adults with heart failure [65]. However, the time at which an endurance programme begin relative to hospital admission is not clear. Given *early intervention* (mobilisation) is recommended for older adults admitted with an exacerbation of heart failure [66] a walking programme with a gradual increase in intensity from early post-admission likely reflects current clinical practice. Whether outcomes would vary for higher dosage and following the use of alternate equipment e.g., cycle ergometers requires additional research.

It was possible to assign treatment ingredients to inpatient rehabilitation interventions. However, for many, the interventions were poorly described limiting exploration of more specific treatment ingredients and/or the ingredient dose. Moreover, the description of usual care comparator groups was limited and those inclusive of rehabilitation could attenuate the between group comparisons for effectiveness. These are not new findings with several previous systematic reviews highlighting the challenges in synthesizing the evidence for rehabilitation interventions [15, 16]. This may have contributed to the observed heterogeneity for some analyses of the current overview. There is a need for more transparent reporting of rehabilitation interventions in line with established frameworks such as the template for intervention description and replication (TIDieR) [67]. A taxonomy of rehabilitation techniques similar to the taxonomy of behaviour change techniques is required for future analyses by individual treatment ingredients and interactions between ingredients [26].

## Conclusion

The designation of treatment ingredients to interventions was challenging due to a paucity of detail specified by published interventions. Despite this, we reported the treatment ingredients *early intervention* and *endurance exercise* were effective at improving endurance, and *early intervention*, *goals and planning*, *repeated practice of activities*, *increased medical care*, and/or *discharge planning* effectively increased the likelihood of discharge to home for older adults following an unplanned admission to hospital. Benefits observed were often for subgroups of the older adult population e.g., *endurance exercise* was effective for endurance in older adults with COPD, and *early intervention* was effective for endurance for those with hip fracture. Future research should seek to determine whether the benefits observed from these treatment ingredients are generalisable to older adults more broadly. Further, there is a need for more transparent reporting of rehabilitation intervention treatment ingredients to enable future synthesis and/or replication. Finally, the challenge of making meaningful change during a short period of inpatient rehabilitation emphasizes the importance of comprehensive post-discharge rehabilitation.

## Supplementary Information

**Additional file 1: Supplementary File 1.** Search strategies. Search strategies for electronic databases of published and unpublished evidence.

**Additional file 2: Supplementary File 2.** Citation matrix. Citation matrix detailing the identification of unique (non-overlapping) randomized controlled trials from systematic reviews included in this overview review.

**Additional file 3: Supplementary File 3.** Characteristics of randomized controlled trials. Characteristics of eligible randomized controlled trials identified from systematic reviews included in this overview review.

**Additional file 4: Supplementary File 4.** Treatment ingredients. Treatment ingredients employed by eligible randomized controlled trials identified from systematic reviews included in this overview review.

**Additional file 5: Supplementary File 5.** Meta-analyses results. Results of meta-analyses (forest plot) of randomized controlled trials identified from systematic reviews included in this overview review for functioning, quality of life, length of stay, discharge destination and mortality. Each meta-analysis is accompanied by a table which describes the characteristics of each randomized controlled trial included in each meta-analysis.

**Additional file 6: Supplementary File 6.** Treatment ingredient meta-analyses results. Results of meta-analyses (forest plot) of randomized controlled trials identified from systematic reviews included in this overview review by treatment ingredient.

**Additional file 7: Supplementary File 7.** Narrative results. Results of randomized controlled trials identified from systematic reviews included in this overview review which were not incorporated into the meta-analyses and reasons why they were not incorporated.

## Data Availability

This overview review reflects a synthesis of previously published randomized controlled trials. All data generated or analysed during this study are included in this published article (and its supplementary files).

## Abbreviations

ADL: Activities of daily living
CI: Confidence Interval
OR: Odds ratio
ICF: International classification of functioning
PRISMA: Preferred Reporting Items for Systematic Reviews and Meta-Analysis
RCT: Randomised controlled trial
AMSTAR: Assessment of Multiple Systematic Reviews
