# Supplementary material for: Effect of inpatient rehabilitation treatment ingredients on functioning, quality of life, length of stay, discharge destination, and mortality among older adults with unplanned admission: an overview review
Source: BMC Geriatr. 2022 Jun 11;22:501. doi: 10.1186/s12877-022-03169-2 (PMC9188066; doi:10.1186/s12877-022-03169-2)
Supplement: Supplementary file 1 — Additional file 1: Supplementary File 1. Search strategies. Search strategies for electronic databases of published and unpublished evidence. [file 12877_2022_3169_MOESM1_ESM.docx]

**Supplementary File 1**

| **Source** | **Search Strategy** |
| --- | --- |
| Cochrane Library | #1 MeSH descriptor: [Rehabilitation] explode all trees  #2 rehab*  #3 MeSH descriptor: [Exercise] explode all trees  #4 exercis*  #5 MeSH descriptor: [Physical Therapy Modalities] explode all trees  #6 physiotherap*  #7 OR #2 OE #3 OR #4 OR #5 OR #6  #8 MeSH descriptor: [Inpatients] explode all trees  #9 inpatient  #10 “acute care”  #11 MeSH descriptor: [Subacute Care] this term only  #12 subacute care  #13 MeSH descriptor: [Subacute Care] this term only  #14 postacute care  #15 #8 OR #9 OR #10 OR #11 OR #12 OR #13 OR #14  #16 (#1 OR #2 OE #3 OR #4 OR #5 OR #6) AND (#8 OR #9 OR #10 OR #11 OR #12 OR #13 OR #14) |
| MEDLINE | 1 exp Rehabilitation/  2 rehabilitation.m_titl.  3 exp Exercise/  4 exercise.m_titl.  5 rehab*.mp.  6 exercis*.mp.  7 “physical therap*”.mp.  8 1 or 2 or 3 or 4 or 5 or 6 or 7  9 “acute care”.mp.  10 exp Subacute Care/  11 “subacute care”.mp.  12 “postacute care”.mp.  13 exp Inpatients/  14 inpatient*mp.  15 9 or 10 or 11 or 12 or 13 or 14  16 exp “Systematic Review”/  17 “systematic review”.mp  18 exp Meta-Analysis/  19 “meta analys*”.mp  20 16 or 17 or 18 or 19  21 8 and 15 and 20 |
| EMBASE | 1 exp rehabilitation/  2 rehab*.mp.  3 exp exercise/  4 exercise*.mp.  5 exp physiotherapy/  6 physical therapy.mp.  7 1 or 2 or 3 or 4 or 5 or 6  8 inpatient.mp.  9 exp subacute care/  10 subacute care.mp.  11 postacute care.mp.  12 acute care.mp.  13 8 or 9 or 10 or 11 or 12  14 exp “systematic review”/  15 systematic review.mp.  16 exp meta analysis/  17 meta analys*mp.  18 14 or 15 or 16 or 17  19 7 and 13 and 18 |
| PsycINFO | 1 exp Rehabilitation/  2 rehabilitation.mp.  3 exp Exercise/  4 exercise.mp.  5 rehab*.mp.  6 exercis*.mp.  7 exp Physical Therapy/  8 physiotherapy.mp.  9 1 or 2 or 3 or 4 or 5 or 6 or 7 or 8  10 inpatient.mp.  11 acute care.mp.  12 subacute care.mp.  13 exp Hospitalized Patients/  14 postacute care.mp.  15 10 or 11 or 12 or 13 or 14  16 exp “Systematic Review”/  17 systematic review.mp.  18 exp Meta Analysis/  19 meta-analysis.mp.  20 16 or 17 or 18 or 19  21 9 and 15 and 20 |
| PEDRo | 'systematic review' with each of:  acute care”  OR “sub-acute care”  OR “subacute care”  OR “sub-acute”  OR subacute  OR “post-acute care”  OR “post-acute”  OR “postacute care”  OR postacute  OR inpatient*  OR “emergency care”  ‘meta-analysis’ with each of:  “acute care”  OR “sub-acute care”  OR “subacute care”  OR “sub-acute”  OR subacute  OR “post-acute care”  OR “post-acute”  OR “postacute care”  OR postacute  OR inpatient*  OR “emergency care” |
| OpenGrey | Systematic review  Meta-analysis |
| BASE | systematic review (entire document row) and  subj:inpatient* |
