# Supplementary material for: Effect of inpatient rehabilitation treatment ingredients on functioning, quality of life, length of stay, discharge destination, and mortality among older adults with unplanned admission: an overview review
Source: BMC Geriatr. 2022 Jun 11;22:501. doi: 10.1186/s12877-022-03169-2 (PMC9188066; doi:10.1186/s12877-022-03169-2)
Supplement: Supplementary file 2 — Additional file 2: Supplementary File 2. Citation matrix. Citation matrix detailing the identification of unique (non-overlapping) randomized controlled trials from systematic reviews included in this overview review. [file 12877_2022_3169_MOESM2_ESM.docx]

Supplementary File 2

| Review with unique studies | Review Author, Year | RCT Author, Year | Unique RCT? |
| --- | --- | --- | --- |
| Yes | Machado 2020 | Borges 2014 | Yes |
|  |  | Greulich 2014 | Yes |
|  |  | He 2015 | Yes |
|  |  | Kirsten 1998 | Yes |
|  |  | Liao 2015 | Yes |
|  |  | Lopez-Lopez 2018 | Yes |
|  |  | Lopez-Lopez 2019a | Yes |
|  |  | Lopez-Lopez 2019b | Yes |
|  |  | Nava 1998 | Yes |
|  |  | Torres Sanchez 2017 | Yes |
|  |  | Torres Sanchez 2018 | Yes |
|  |  | Torres-Sanchez 2016 | Yes |
| Yes | Peck 2020 | Resnick 2016 | Yes |
| Yes | Smith, 2020a | Marcantonio, 2001 | Yes |
| Yes | Smith 2020b | Counsell 2000 | Yes |
|  |  | Landefeld 1995 | Yes |
|  |  | Lenze 2012 | Yes |
|  |  | Timmer 2019 | Yes |
| Yes | Yasmeen 2020 | Louie 2012 | Yes |
| Yes | Heldmann 2019 | Counsell 2000 | No |
|  |  | He 2015 | No |
|  |  | Huusko 2000 | No |
|  |  | Landefeld 1995 | No |
|  |  | Torres-Sanchez 2017 | No |
|  |  | Abizanda 2011 | Yes |
|  |  | Asplund 2000 | Yes |
|  |  | Barnes 2012 | Yes |
|  |  | Blanc-Bisson 2008 | Yes |
|  |  | Brown 2016 | Yes |
|  |  | Hagsten 2004 | Yes |
|  |  | Jeffs 2013 | Yes |
|  |  | Jones 2006 | Yes |
|  |  | Kimmel 2016 | Yes |
|  |  | Naglie 2002 | Yes |
|  |  | Oldmeadow 2006 | Yes |
|  |  | Prestmo 2015 | Yes |
|  |  | Stenvall 2007 | Yes |
|  |  | Vidan 2005 | Yes |
| No | Hu 2019 | Nava 1998 | No |
| No | Talevski, 2019 | Naglie 2002 | No |
|  |  | Prestmo 2015 | No |
|  |  | Stenvall 2007 | No |
|  |  | Vidan 2005 | No |
| Yes | Peiris 2018 | Kimmel 2016 | No |
|  |  | Peiris 2013 | Yes |
| Yes | Martinez-Velilla 2016 | Abizanda 2011 | No |
|  |  | Asplund 2000 | No |
|  |  | Blanc-Bisson 2008 | No |
|  |  | Counsell 2000 | No |
|  |  | Jones 2006 | No |
|  |  | Landefeld 1995 | No |
|  |  | Saltvedt 2002 | Yes |
|  |  | Tibaek 2014 | Yes |
| No | Puhan, 2016 | Borges, 2014 | No |
|  |  | He, 2015 | No |
|  |  | Kirsten, 1998 | No |
|  |  | Liao, 2015 | No |
|  |  | Nava, 1998 | No |
|  |  | Tang, 2012 | No |
| Yes | Scrivener 2015 | Peiris 2013 | No |
|  |  | Said 2012 | Yes |
| No | Thorne 2014 | Stenvall 2007, Olofsson2007 | No |
| No | Fox 2012 | Asplund 2000 | No |
|  |  | Barnes 2012 | No |
|  |  | Counsell 2000 | No |
|  |  | Landefeld 1995 | No |
|  |  | Stenvall 2007, Olofsson2007 | No |
| Yes | Handoll, 2011 | Oldmeadow, 2016 | No |
|  |  | Baker, 1992 | Yes |
|  |  | Graham, 1968 | Yes |
|  |  | Karumo, 1977 | Yes |
|  |  | Lauridsen, 2002 | Yes |
|  |  | Mitchell, 2001 | Yes |
| Yes | Bachmann 2010 | Naglie 2002 | No |
|  |  | Stenvall 2007 | No |
|  |  | Saltvedt 2002 | No |
|  |  | Swanson 1998 | Yes |
| No | Crotty, 2010 | Hagsten, 2004 | No |
| No | Van Craen 2010 | Counsell 2000 | No |
|  |  | Landefeld 1995 | No |
|  |  | Saltvedt 2002-2006 | No |
| No | Chudyk 2009 | Hagsten 2004 | No |
|  |  | Hagsten 2006 | No |
|  |  | Huusko 2000 | No |
|  |  | Lauridsen 2002 | No |
|  |  | Mitchell 2001 | No |
|  |  | Naglie 2002 | No |
|  |  | Swanson 1998 | No |
| No | Handoll, 2009 | Huusko, 2002 | No |
|  |  | Naglie, 2002 | No |
|  |  | Stenvall, 2007 | No |
|  |  | Swanson, 1998 | No |
|  |  | Vidan, 2005 | No |
| Yes | De Morton 2007 | Asplund 2000 | No |
|  |  | Counsell 2000 | No |
|  |  | Jones 2006 | No |
|  |  | Landefeld 1995 | No |
|  |  | Slaets 1997 | Yes |
| No | Cameron 2000 | Naglie 2002 | No |
|  |  | Swanson 1998 | No |

RCT: randomised controlled trial. RCTs in grey are duplicates. Reviews in grey are reviews with no unique studies after removing duplicates.
