# Supplementary material for: Effect of inpatient rehabilitation treatment ingredients on functioning, quality of life, length of stay, discharge destination, and mortality among older adults with unplanned admission: an overview review
Source: BMC Geriatr. 2022 Jun 11;22:501. doi: 10.1186/s12877-022-03169-2 (PMC9188066; doi:10.1186/s12877-022-03169-2)
Supplement: Supplementary file 3 — Additional file 3: Supplementary File 3. Characteristics of randomized controlled trials. Characteristics of eligible randomized controlled trials identified from systematic reviews included in this overview review. [file 12877_2022_3169_MOESM3_ESM.docx]

Supplementary File 3: Characteristics of 41 unique randomized controlled trials identified from 12 systematic reviews relevant to the current overview review.

| **Systematic Review** | **Randomized controlled trial** | **Population** | **Sample size I:C*** | **Intervention** | **Comparator*** | **Outcome measure** | **Follow up** |
| --- | --- | --- | --- | --- | --- | --- | --- |
| Bachmann 2010 | Swanson 1998 | Hip fracture  I: mean age 78.5 years; gender m:f 11:27; from home 92.1%  C: mean age 77.8 years; gender m:f 5:28; from home 87.9% | 38:33 | Repeated exercise rehabilitation; Team meetings & care planning; Discharge planning; Increased medical care; Early intervention; Home visit | Usual care (Australia). Physiotherapy. Occupational therapy on referral. | Modified Barthel Index, length of stay, discharge destination, mortality | 1 month |
| de Morton 2007 | Slaets 1997 | Medical admission (referred to Department of General Medicine on admission)  mean age 83 years, gender m:f 69:198; from home 72% | 140:97 | Repeated exercise rehabilitation; Team meetings & care planning; Increased medical care | Usual care (The Netherlands). Services provided by physicians and nurses. | SIVIS dependency scale, length of stay, mortality |  |
| Handoll 2011 | Baker 1991 | Hip fracture  mean age 83.5 years, m:f 0:12 | 6:6 | Endurance exercise; Antecedents | Usual care (Australia) including conventional gait training with ambulatory aids. | walking speed, length of stay |  |
| Handoll 2011 | Graham 1968 | Hip fracture | 141:132 | Endurance exercise; Early intervention | Delayed weight bearing until 12 weeks after surgery | 12 months |  |
| Handoll 2011 | Karumo 1977 | Hip fracture  mean age 80 years, gender m:f 22:65 | 39:48 | Repeated practice activities (+/- increasing demands); Repeated exercise rehabilitation | Usual care (Finland). Physiotherapy 30 minutes. | length of stay |  |
| Handoll 2011 | Lauridsen 2002 | Hip fracture  mean age 80 years, gender m:f 0:51 | 20:31 | Repeated practice functions (+/- increasing demands); Repeated practice activities (+/- increasing demands) | Usual care (Denmark). Physiotherapy 15-30 minutes per weekday. | length of stay |  |
| Handoll 2011 | Mitchell 2001 | Hip fracture  mean age 80 years, gender m:f 13:67 | 40:40 | Strengthening exercise; Repeated practice functions (+/-increasing demands) | Usual care (UK). 20 minutes physiotherapy per weekday. | Elderly Mobility Scale, Barthel Index, walking speed, Nottingham Power Rig leg extensor power (watts) | 16 weeks |
| Heldmann 2019 | Abizanda 2011 | Medical admission (acute medical illness e.g., pneumonia or exacerbation of previous chronic condition)  mean age 84 years, gender m:f 187:227 | 198:202 | Repeated practice activities (+/- increasing demands); Goals and planning; Shaping knowledge; Cognitive orientation exercise | Usual care (Spain). Includes physiotherapy. | Barthel Index, length of stay |  |
| Heldmann 2019 | Asplund 2000 | Medical admission (main presenting symptoms of chest pain, other pain, dyspnea, nausea/vomiting, vertigo)  mean age 81 years, gender m:f 162:251 | 190:223 | Early intervention; Discharge planning; Increased medical care | Usual care (Sweden). Physiotherapy and occupational therapy not routinely available. | Discharge destination, mortality | 3 months |
| Heldmann 2019 | Barnes 2012 | Medical admission (acute medical illness including pulmonary, gastrointestinal, cardiovascular, infection, neurological)  mean age 80.6 years, gender m:f 538:1094, from home 84% | 858:774 | Repeated practice activities (+/- increasing demands); Antecedents; Team meetings & care planning; Discharge planning; Increased medical care; Nutritional intervention | Usual care (USA). Physiotherapy if referred. | Katz ADL Index, discharge destination, mortality |  |
| Heldmann 2019 | Blanc-Bisson 2008 | Medical admission (admitted to acute-care geriatric medicine unit)  mean age 85 years, gender m:f 21:55 | 38:38 | Strengthening exercises; Repeated practice functions | Usual care (France). Physiotherapy. | Katz ADL |  |
| Heldmann 2019 | Brown 2016 | Medical admission (admitted to medical wards, common diagnoses included pneumonia, heart facilure and COPD)  mean age 74 years, gender m:f 97:3 | 50:50 | Endurance exercise; Goals and planning; Feedback and monitoring | Usual care (USA). Allied health if referred. | modified Katz ADL Index, length of stay |  |
| Heldmann 2019 | Hagsten 2004 | Hip fracture  mean age 80 years, gender m:f 20:80 | 50:50 | Repeated practice activities; Shaping knowledge; Home visit | Usual care (Sweden). Physiotherapy. | Modified Klein-Bell ADL Scale; |  |
| Heldmann 2019 | Jeffs 2013 | Medical admission (admitted to medical unit)  mean age 79 years, gender m:f 308:340 | 305:343 | Strengthening exercise; Feedback and monitoring; Shaping knowledge; Cognitive orientation exercise | Usual care (Australia). Allied health if referred. | length of stay |  |
| Heldmann 2019 | Jones 2006 | Medical admission (admitted to general medical ward)  mean age 82 years, gender m:f 68:92 | 80:80 | Strengthening exercise; Repeated practice activities (+/- increasing demands) | Usual care (Australia). Standard physiotherapy. | length of stay, discharge destination, mortality |  |
| Heldmann 2019 | Kimmel 2016 | Hip fracture  mean age 81 years, gender m:f 33:59 | 46:46 | Repeated exercise rehabilitation | Usual care (Australia). Daily physiotherapy. | Timed Up and Go, length of stay, discharge destination |  |
| Heldmann 2019 | Naglie 2002 | Hip fracture  mean age 84 years, gender m:f 56:223 | 141:138 | Repeated practice activities (+/- increasing demands); Feedback and monitoring; Shaping knowledge; Team meetings & care planning; Discharge planning; Increased medical care; Early intervention; Home visit | Usual care (Canada). Physiotherapy if referred. Occupational therapy rarely. | length of stay, discharge destination, mortality | 6 months |
| Heldmann 2019 | Oldmeadow 2006 | Hip fracture  I: mean age 78.8 years, gender m:f 8:21  C: mean age 80.8 years, gender m:f 11:20 | 29:31 | Early intervention | Delayed assisted ambulation to post op day 3 or 4 | Mean walking distance at 7 days (metres), length of stay, discharge destination, mortality |  |
| Heldmann 2019 | Prestmo 2015 | Hip fracture  I: mean age 83.4 years, gender m:f 53:145, from home alone 58%  C: mean age 83.2 years, gender m:f 51:148, from home alone 48% | 198:199 | Repeated practice activities (+/- increasing demands); Goals and planning; Team meetings & care planning; Discharge planning; Increased medical care; Nutritional intervention; Early intervention | Usual care (Norway). Physiotherapy according to guidelines. No occupational therapists. | Timed Up and Go, Barthel Index, EQ-5D, length of stay, discharge destination | 12 months |
| Heldmann 2019 | Stenvall 2007 | Hip fracture  mean age 82 years, gender m:f 51:148, from home 36.7% | 102:97 | Endurance exercise; Repeated practice activities (+/- increasing demands); Goals and planning; Shaping knowledge; Team meetings & care planning; Increased medical care; Nutritional intervention; Early intervention | Usual care (Sweden). Exercise rehabilitation with daily physiotherapy and occupational therapy as needed. | Less dependent, based on Katz Index, length of stay, mortality | 12 months |
| Heldmann 2019 | Vidan 2005 | Hip fracture  mean age 82 years, gender m:f 59:260, from home alone 84.3% | 155:164 | Repeated exercise rehabilitation; Antecedents; Team meetings & care planning; Increased medical care | Usual care (Spain) Physiotherapy. Occupational therapy not available. | length of stay, mortality | 12 months |
| Machado 2020 | Borges 2014 | COPD  I: mean age 64.1 years, gender m:f 8:7  C:mean age 67.8 years, gender m:f 10:4 | 15:14 | Repeated practice functions (+/-increasing demands); Shaping knowledge; Comparison of behavior | Usual care (Brazil). Chest physiotherapy and advice re physical activity. | Change in 6-minute walk test, St George’s Respiratory Questionnaire |  |
| Machado 2020 | Greulich 2014 | COPD  I:mean age 66.4 years, gender m:f 14:6  C: mean age 70.4 years, gender m:f 12:8 | 20:20 | Energy applied to soft tissue; Early intervention | Usual care (Germany). 20 minutes physiotherapy. | 6-minute walk test (metres), Chair rising test, St George’s Respiratory Questionnaire, length of stay |  |
| Machado 2020 | He 2015 | COPD  I:mean age 69.2 years, gender m:f 6:60  C: mean age 73.9 years, gender m:f 5:23 | 66:28 | Strengthening exercise; Endurance exercise; Energy applied to soft tissue; Breathing related exercise/training; Shaping knowledge; Natural consequences | Usual care (China) | 6-minute walk test (metres) |  |
| Machado 2020 | Kirsten 1998 | COPD  I: mean age 62.3 years, gender m:f 12:3  C: mean age 65.6 years, gender m:f 14:0 | 15:14 | Endurance exercise | Usual care (Germany). No regular exercise, walking assessments on 4 days. | 6-minute walk test (metres) |  |
| Machado 2020 | Liao 2015 | COPD  I:mean age 68 years, gender m:f 16:14  C: mean age 70 years, gender m:f 21:10 | 30:31 | Endurance exercise; Breathing related exercise/ training; Shaping knowledge; Natural consequences; Nutritional intervention | Usual care (Taiwan). Health education, monitoring of vital signs and symptoms, assessing nutritional status, and nasal oxygen therapy | Change in 6-minute walk test |  |
| Machado 2020 | Lopez-Lopez 2018 (1) | COPD  I:mean age 62.8 years, gender m:f 11:3  C: mean age 64.3 years, gender m:f 10:2 | 14:12 | Energy applied to soft tissue | Usual care (Spain). Medical care alone. | London Chest Activity of Daily Living Score, 5-times sit to stand, length of stay |  |
| Machado 2020 | Lopez-Lopez 2018 (2) | COPD  I:mean age 63.9 years, gender m:f 10:3  C: mean age 64.3, years, gender m:f 10:2 | 13:12 | Strengthening exercise | Usual care (Spain). Medical care alone. | 5-times sit to stand, London Chest Activity of Daily Living Score, length of stay |  |
| Machado 2020 | Lopez-Lopez 2019a (1) | COPD  I: mean age 71.2 years  C:mean age 71.35 years | 22:22 | Endurance exercise; Energy applied to soft tissue; Goals and planning; Feedback and monitoring | Usual care (Spain). Medical care alone. | Functional Independence Measure, 5-times sit to stand, EQ-5D, length of stay |  |
| Machado 2020 | Lopez-Lopez 2019a (2) | COPD  I: mean age 72.63 years  C: mean age 71.35 years | 22:22 | Endurance exercise; Energy applied to soft tissue; Goals and planning; Feedback and monitoring | Usual care (Spain). Medical care alone. | Functional Independence Measure, 5-times sit to stand, EQ-5D, length of stay |  |
| Machado 2020 | Lopez-Lopez 2019b (1) | COPD  I: mean age: 71.7 years  C: mean age 68.5 years | 15:16 | Energy applied to soft tissue; Repeated practice functions (+/-increasing demands) | Usual care (Spain). Medical care alone. | length of stay |  |
| Machado 2020 | Lopez-Lopez 2019b (2) | COPD  I: mean age 68.6 years  C: mean age 68.5 years | 17:16 | Energy applied to soft tissue; Breathing related exercise/ training; Repeated practice functions (+/-increasing demands); Goals and planning; Feedback and monitoring; Shaping knowledge; Natural consequences | Usual care (Spain). Medical care alone. | length of stay |  |
| Machado 2020 | Nava 1998 | COPD  mean age 66 years; m:f 51:29 | 60:20 | Strengthening exercise; Endurance exercise; Breathing related exercise/training | Usual care (Italy). Progressive ambulation programme. | 6-minute walk test |  |
| Machado 2020 | Torres-Sanchez 2016 | COPD  I: mean age 72.4 years, gender m:f 24:0  C: mean age 73.7 years, gender m:f 23:2 | 24:25 | Endurance exercise; Shaping knowledge | Usual care (Spain). Medical care alone. | 2-minutes step in place test (number of repetitions), EQ-5D, length of stay |  |
| Machado 2020 | Torres-Sanchez 2017 | COPD  I: mean age: 75.7 years, gender m:f 22:7  C: mean age 72.1 years, gender m:f 20:9 | 29:29 | Endurance exercise; Shaping knowledge | Usual care (Spain). Medical care alone. | Steps per day, 30-second sit to stand (number of repetitions), length of stay |  |
| Machado 2020 | Torres-Sanchez 2018 (1) | COPD  I: mean age 75.1 years, gender m:f 28:2  C: mean age 71.1 years, gender m:f 24.6 | 30:30 | Breathing related exercise/ training; Repeated practice functions (+/-increasing demands); Feedback and monitoring | Usual care (Spain). Medical care alone. | EQ-5D, length of stay |  |
| Martinez-Velilla, 2016 | Saltvedt 2002 | Medical admission (admitted to Internal Medicine)  I: mean age 81.4 years, gender m:f 46:81, from home 91%  C: mean age 82.4 years, gender m:f 43:84, from home 87% | 127:127 | Shaping knowledge; Antecedents; Team meetings & care planning; Discharge planning; Increased medical care; Early intervention; Home visit | Usual care (Norway), Allied health if referred. | mortality | 12 months |
| Martinez-Velilla 2016 | Tibaek 2014 | Older adult (admitted to the Department of Geriatric Rehabilitation, diagnoses included cancer, lung disease, falls, fracture or allopathic in lower extremities, stroke, Parkinsons disease, pancreatitis, heart disease, medicine disease, back disease)  I: mean age 80 years  C: mean age 79 years  gender m:f 30:41, from home alone 62% | 36:35 | Strengthening exercise | Usual care (Denmark). Physiotherapy. | Barthel Index, 10-metre walk test |  |
| Peck 2020 | Resnick 2016 | Orthopaedic trauma  mean age 80 years, gender m:f 30:59 | 50:39 | Repeated practice activities (+/- increasing demands); Goals and planning; Feedback and monitoring; Shaping knowledge; Antecedents; Increased medical care | Usual care (USA) plus education. | Physical Performance and Mobility Examination, Barthel Index, discharge destination, mortality | 1 month |
| Peiris 2018 | Peiris 2013 | Medical admission (orthopaedic, pain, cardiac/pulmonary, neurological, other disabling impairment)  I: mean age 75 years, gender m:f 188:308, from home 94%  C: mean age 74 years, gender m:f 171:329, from home 93% | 496:500 | Repeated exercise rehabilitation | Usual care (Australia). Physiotherapy weekdays. | Timed Up and Go, Functional Independence Measure, 10-metre walk test, EQ-5D, length of stay | 12 months |
| Scrivener 2015 | Said 2012 | Older adult (musculoskeletal, cardiac/respiratory, other surgical, neurological, falls/functional decline)  I: mean age 80.8 years, gender m:f 9:13  C: mean age 81.6 years, m:f 15:10 | 22:25 | Endurance exercise; Repeated practice activities (+/- increasing demands) | Usual care (Australia). Physiotherapy 1-2 sessions on weekdays. | Barthel Index, length of stay | 3 months |
| Smith 2020a | Marcantonio 2001 | Hip fracture  I: mean age 78 years, gender m:f 13:49  C: mean age 80 years, gender m:f 14:50 | 62:64 | Antecedents; Increased medical care; Nutritional intervention; Early intervention | Usual care (USA). Medical care. | length of stay, discharge destination |  |
| Smith 2020b | Lenze 2012 | Medical admission (cardiovascular problem, stroke, hip fracture, cervical spine fusion, colectomy, tibial fracture)  I: mean age 80.2 years, gender m:f 4:10  C: mean age 75.7 years, gender m:f 2:10 | 14:12 | Repeated exercise rehabilitation; Goals and planning; Feedback and monitoring | Usual care (USA). Physiotherapy and occupational therapy. | Barthel Index, walking speed, 6 minute walk test |  |
| Smith 2020b | Counsell 2020 | Medical admission (cardiac, infection, pulmonary, neurological, gastrointestinal, diabetes, failure to thrive)  I: mean age 80 years, gender m:f 305:462, from home 100%  C: mean age 79 years, gender m:f 300:464, from home 100% | 767:764 | Repeated practice activities (+/- increasing demands); Antecedents; Team meetings & care planning; Discharge planning; Increased medical care; Nutritional intervention | Usual care (USA). Medical care. | Independent Activities of Daily Living, modified Katz ADL Index, length of stay, mortality | 3 months |
| Smith 2020b | Timmer 2019 | Medical admission (respiratory, general medicine, digestive, circulatory, kidney)  I: mean age 80 years, gender m:f 14:37, from home 63%  C: mean age 81 years, gender m:f 12:37, from home 57% | 51:48 | Repeated practice activities (+/- increasing demands); Goals and planning; Feedback and monitoring; Social support; Shaping knowledge; Natural consequences | Usual care (Australia) plus a brief activity pacing education. Physiotherapy and occupational therapy. | Functional Independence Measure, length of stay, mortality | 3 months |
| Smith 2020b | Landefeld 1995 | Medical admission (cardiac, neurological, infection, pulmonary, gastrointestinal, diabetes, failure to thrive)  I: mean age 80.2 years, gender m:f 104:223, from home 93%  C: mean age 80.1 years, gender m:f 112:212, from home 91% | 327:324 | Repeated practice activities (+/- increasing demands); Antecedents; Team meetings & care planning; Discharge planning; Increased medical care; Nutritional intervention | Usual care (USA). Physiotherapy. | Katz ADL Index, length of stay, discharge destination, mortality | 3 months |
| Yasmeen 2020 | Louie 2012 | Hip fracture  I: mean age 77.81 years, gender m:f 11:52  C: mean age 78.7 years, gender m:f 14:57 | 63:71 | Repeated practice activities (+/- increasing demands); Goals and planning; Feedback and monitoring; Social support; Shaping knowledge; Natural consequences; Comparison of behavior | Usual care (Hong Kong, China). Hip fracture protocol including ADL training. | Functional Independence Measure (motor scores) |  |

I:C = intervention: control. m: f = male: female. ADL = activities of daily living. COPD = chronic obstructive pulmonary disease. EQ-5D = EuroQol 5 dimensions. SIVIS = the Health Care Information Center Foundation.
*comparator and extent to which exercise/professionals who prescribe exercise were involved in the usual care arm.
