# Supplementary material for: Effect of inpatient rehabilitation treatment ingredients on functioning, quality of life, length of stay, discharge destination, and mortality among older adults with unplanned admission: an overview review
Source: BMC Geriatr. 2022 Jun 11;22:501. doi: 10.1186/s12877-022-03169-2 (PMC9188066; doi:10.1186/s12877-022-03169-2)
Supplement: Supplementary file 4 — Additional file 4: Supplementary File 4. Treatment ingredients. Treatment ingredients employed by eligible randomized controlled trials identified from systematic reviews included in this overview review. [file 12877_2022_3169_MOESM4_ESM.docx]

Supplementary File 4

| **Review author, year** | **RCT author, year** | | | | | **Population** | | **ORGAN FUNCTIONS** | | | | | | | | | | | | | |
| --- | --- | --- | --- | --- | --- | --- | --- | --- | --- | --- | --- | --- | --- | --- | --- | --- | --- | --- | --- | --- | --- |
|  |  | | | | |  | | **Strengthening exercise** | | | **Endurance exercise** | | | | **Energy applied to soft tissue** | | | | | **Breathing related exercise/ training** | |
| Handoll, 2011 | Baker, 1991 | | | | | hip fracture | | 0 | | | 1 | | | | 0 | | | | | 0 | |
|  | Graham, 1968 | | | | | hip fracture | | 0 | | | 1 | | | | 0 | | | | | 0 | |
|  | Mitchell, 2001 | | | | | hip fracture | | 1 | | | 0 | | | | 0 | | | | | 0 | |
| Heldmann, 2019 | Stenvall, 2007 | | | | | hip fracture | | 0 | | | 1 | | | | 0 | | | | | 0 | |
|  | Blanc-Bisson, 2008 | | | | | medical admission | | 1 | | | 0 | | | | 0 | | | | | 0 | |
|  | Brown, 2016 | | | | | medical admission | | 0 | | | 1 | | | | 0 | | | | | 0 | |
|  | Jeffs, 2013 | | | | | medical admission | | 1 | | | 0 | | | | 0 | | | | | 0 | |
|  | Jones, 2006 | | | | | medical admission | | 1 | | | 0 | | | | 0 | | | | | 0 | |
| Machado, 2020 | Greulich, 2014 | | | | | copd | | 0 | | | 0 | | | | 1 | | | | | 0 | |
|  | Torres-Sanchez, 2017 | | | | | copd | | 0 | | | 1 | | | | 0 | | | | | 0 | |
|  | Torres-Sanchez 2016 | | | | | copd | | 1 | | | 0 | | | | 0 | | | | | 1 | |
|  | Lopez-Lopez 2019a (1) | | | | | copd | | 0 | | | 1 | | | | 1 | | | | | 0 | |
|  | Lopez-Lopez 2019a (2) | | | | | copd | | 0 | | | 1 | | | | 1 | | | | | 0 | |
|  | Lopez-Lopez 2019b (1) | | | | | copd | | 0 | | | 0 | | | | 1 | | | | | 0 | |
|  | Lopez-Lopez 2019b (2) | | | | | copd | | 0 | | | 0 | | | | 1 | | | | | 1 | |
|  | Torres-Sanchez 2018 (1) | | | | | copd | | 0 | | | 0 | | | | 0 | | | | | 1 | |
|  | Torres-Sanchez 2018 (2) | | | | | copd | | 1 | | | 0 | | | | 0 | | | | | 0 | |
|  | Lopez-Lopez 2018 (1) | | | | | copd | | 0 | | | 0 | | | | 1 | | | | | 0 | |
|  | Lopez-Lopez 2018 (2) | | | | | copd | | 1 | | | 0 | | | | 0 | | | | | 0 | |
|  | Borges, 2014 | | | | | copd | | 1 | | | 0 | | | | 0 | | | | | 0 | |
|  | He, 2015 | | | | | copd | | 1 | | | 1 | | | | 0 | | | | | 1 | |
|  | Kirsten, 1998 | | | | | copd | | 0 | | | 1 | | | | 0 | | | | | 0 | |
|  | Liao, 2015 | | | | | copd | | 0 | | | 1 | | | | 0 | | | | | 1 | |
|  | Nava, 1998 | | | | | copd | | 1 | | | 1 | | | | 0 | | | | | 1 | |
|  | Tang, 2012 | | | | | copd | | 1 | | | 1 | | | | 0 | | | | | 0 | |
| Martinez-Vellilla, 2016 | Tibaek 2014 | | | | | older adult | | 1 | | | 0 | | | | 0 | | | | | 0 | |
| Scrivener, 2015 | Said, 2012 | | | | | older adult | | 0 | | | 1 | | | | 0 | | | | | 0 | |
| **Total** |  | | | | |  | | **12** | | | **13** | | | | **7** | | | | | **6** | |
|  |  | | | | |  | |  | | |  | | |  |  | | | | |  | |
| **Review Author, year** | | **RCT author, year** | | | **Population** | | | **SKILLS AND HABITS** | | | | | | | | | | | | | |
|  | |  | | |  | | | **Repeated practice functions (+/-increasing demands)** | | | | **Repeated practice activities (+/- increasing demands)** | | | | | **Repeated exercise rehabilitation,  detail not specified** | | | | |
| Bachmann, 2010 | | Swanson, 1998 | | | hip fracture | | | 0 | | | | 0 | | | | | 1 | | | | |
| de Morton, 2007 | | Slaets 1997 | | | medical admission | | | 0 | | | | 0 | | | | | 1 | | | | |
| Handoll, 2011 | | Karumo, 1977 | | | hip fracture | | | 1 | | | | 1 | | | | | 0 | | | | |
|  | | Lauridsen, 2002 | | | hip fracture | | | 1 | | | | 1 | | | | | 0 | | | | |
|  | | Mitchell, 2001 | | | hip fracture | | | 1 | | | | 0 | | | | | 0 | | | | |
| Heldmann, 2019 | | Hagsten, 2004 | | | hip fracture | | | 0 | | | | 1 | | | | | 0 | | | | |
|  | | Counsell 2000 | | | medical admission | | | 0 | | | | 1 | | | | | 0 | | | | |
|  | | Landefeld 1995 | | | medical admission | | | 0 | | | | 1 | | | | | 0 | | | | |
|  | | Naglie, 2002 | | | hip fracture | | | 0 | | | | 1 | | | | | 0 | | | | |
|  | | Stenvall, 2007 | | | hip fracture | | | 0 | | | | 1 | | | | | 0 | | | | |
|  | | Vidan, 2005 | | | hip fracture | | | 0 | | | | 0 | | | | | 1 | | | | |
|  | | Kimmel, 2016 | | | hip fracture | | | 0 | | | | 0 | | | | | 1 | | | | |
|  | | Abizanda, 2011 | | | medical admission | | | 0 | | | | 1 | | | | | 0 | | | | |
|  | | Blanc-Bisson, 2008 | | | medical admission | | | 1 | | | | 0 | | | | | 0 | | | | |
|  | | Jones, 2006 | | | medical admission | | | 0 | | | | 1 | | | | | 0 | | | | |
|  | | Barnes, 2012 | | | medical admission | | | 0 | | | | 1 | | | | | 0 | | | | |
|  | | Prestmo, 2015 | | | hip fracture | | | 0 | | | | 1 | | | | | 0 | | | | |
| Machado, 2020 | | Torres-Sanchez 2016 | | | copd | | | 1 | | | | 0 | | | | | 0 | | | | |
|  | | Lopez-Lopez 2019b (1) | | | copd | | | 1 | | | | 0 | | | | | 0 | | | | |
|  | | Lopez-Lopez 2019b (2) | | | copd | | | 1 | | | | 0 | | | | | 0 | | | | |
|  | | Torres-Sanchez 2018 (1) | | | copd | | | 1 | | | | 0 | | | | | 0 | | | | |
| Peck,2020 | | Resnick, 2016 | | | orthopaedic trauma | | | 0 | | | | 1 | | | | | 0 | | | | |
| Peiris, 2018 | | Peiris, 2013 | | | medical admission | | | 0 | | | | 0 | | | | | 1 | | | | |
| Scrivener, 2015 | | Said, 2012 | | | older adult | | | 0 | | | | 1 | | | | | 0 | | | | |
| Smith, 2020b | | Lenze, 2012 | | | medical admission | | | 0 | | | | 0 | | | | | 1 | | | | |
| Smith, 2020b | | Timmer, 2019 | | | medical admission | | | 0 | | | | 1 | | | | | 0 | | | | |
| Yasmeen, 2020 | | Louie, 2012 | | | hip fracture | | | 0 | | | | 1 | | | | | 0 | | | | |
| **Total** | |  | | |  | | | **8** | | | | **15** | | | | | **6** | | | | |
|  | |  | | |  | | |  | | | |  | | | | |  | | | | |
|  | |  | | |  | | |  | | | |  | | | | |  | | | | |
|  | |  | | |  | | |  | | | |  | | | | |  | | | | |
|  | |  | | |  | | |  | | | |  | | | | |  | | | | |
|  | |  | | |  | | |  | | | |  | | | | |  | | | | |
|  | |  | | |  | | |  | | | |  | | | | |  | | | | |
|  | |  | | |  | | |  | | | |  | | | | |  | | | | |
| **Review author, year** | | **RCT author, year** | | **Population** | | | | **CHANGING BEHAVIOUR** | | | | | | | | | | | | | |
|  | |  | |  | | | | **Goals and planning** | | **Feedback and monitoring** | | **Social support** | **Shaping knowledge** | | | **Natural consequences** | | **Comparison of behaviour** | | | **Antecedents** |
| Handoll, 2011 | | Baker, 1991 | | hip fracture | | | | 0 | | 0 | | 0 | 0 | | | 0 | | 0 | | | 1 |
| Heldmann, 2019 | | Hagsten, 2004 | | hip fracture | | | | 0 | | 0 | | 0 | 1 | | | 0 | | 0 | | | 0 |
|  | | Counsell 2000 | | medical admission | | | | 0 | | 0 | | 0 | 0 | | | 0 | | 0 | | | 2 |
|  | | Landefeld 1995 | | medical admission | | | | 0 | | 0 | | 0 | 0 | | | 0 | | 0 | | | 2 |
|  | | Naglie, 2002 | | hip fracture | | | | 0 | | 1 | | 0 | 1 | | | 0 | | 0 | | | 0 |
|  | | Stenvall, 2007 | | hip fracture | | | | 1 | | 0 | | 0 | 1 | | | 0 | | 0 | | | 0 |
|  | | Vidan, 2005 | | hip fracture | | | | 0 | | 0 | | 0 | 0 | | | 0 | | 0 | | | 1 |
|  | | Abizanda, 2011 | | medical admission | | | | 1 | | 0 | | 0 | 1 | | | 0 | | 0 | | | 0 |
|  | | Brown, 2016 | | medical admission | | | | 1 | | 1 | | 0 | 0 | | | 0 | | 0 | | | 0 |
|  | | Jeffs, 2013 | | medical admission | | | | 0 | | 1 | | 0 | 1 | | | 0 | | 0 | | | 0 |
|  | | Barnes, 2012 | | medical admission | | | | 0 | | 0 | | 0 | 0 | | | 0 | | 0 | | | 2 |
|  | | Prestmo, 2015 | | hip fracture | | | | 1 | | 0 | | 0 | 0 | | | 0 | | 0 | | | 0 |
|  | | Torres-Sanchez, 2017 | | copd | | | | 0 | | 0 | | 0 | 1 | | | 0 | | 0 | | | 0 |
|  | | Torres-Sanchez 2016 | | copd | | | | 0 | | 1 | | 0 | 1 | | | 0 | | 0 | | | 0 |
|  | | Lopez-Lopez 2019a (1) | | copd | | | | 0 | | 1 | | 0 | 0 | | | 0 | | 0 | | | 0 |
|  | | Lopez-Lopez 2019a (2) | | copd | | | | 0 | | 1 | | 0 | 0 | | | 0 | | 0 | | | 0 |
|  | | Lopez-Lopez 2019b (2) | | copd | | | | 1 | | 2 | | 0 | 1 | | | 1 | | 0 | | | 0 |
|  | | Torres-Sanchez 2018 (1) | | copd | | | | 0 | | 1 | | 0 | 0 | | | 0 | | 0 | | | 0 |
|  | | Torres-Sanchez 2018 (2) | | copd | | | | 0 | | 1 | | 0 | 0 | | | 0 | | 0 | | | 0 |
|  | | He, 2015 | | copd | | | | 0 | | 0 | | 0 | 1 | | | 1 | | 0 | | | 0 |
|  | | Liao, 2015 | | copd | | | | 0 | | 0 | | 0 | 2 | | | 1 | | 0 | | | 0 |
| Martinez-Vellilla, 2016 | | Saltvedt 2002 | | medical admission | | | | 0 | | 0 | | 0 | 1 | | | 0 | | 0 | | | 1 |
|  | | Saltvedt 2006 | | medical admission | | | | 0 | | 0 | | 0 | 1 | | | 0 | | 0 | | | 1 |
| Peck,2020 | | Resnick, 2016 | | orthopaedic trauma | | | | 1 | | 1 | | 0 | 1 | | | 1 | | 0 | | | 1 |
| Smith,2020 | | Marcantonio,2001 | | hip fracture | | | | 0 | | 0 | | 0 | 0 | | | 0 | | 0 | | | 1 |
| Smith, 2020b | | Lenze, 2012 | | medical admission | | | | 2 | | 1 | | 0 | 0 | | | 0 | | 0 | | | 0 |
|  | | Timmer, 2019 | | medical admission | | | | 1 | | 1 | | 1 | 1 | | | 0 | | 0 | | | 0 |
| Yasmeen, 2020 | | Louie, 2012 | | hip fracture | | | | 2 | | 1 | | 1 | 1 | | | 1 | | 1 | | | 0 |
| **Total** | |  | |  | | | | **11** | | **14** | | **2** | **16** | | | **5** | | **1** | | | **12** |
|  | |  | |  | | | |  | |  | |  | |  | |  | |  | | |  |
|  | |  | |  | | | |  | |  | |  | |  | |  | |  | | |  |
|  | |  | |  | | | |  | |  | |  | |  | |  | |  | | |  |
|  | |  | |  | | | |  | |  | |  | |  | |  | |  | | |  |
| **Review author, year** | **RCT author, year** | | **Population** | | | | **OTHER INTERVENTION COMPONENTS** | | | | | | | | | | | | | | |
|  |  | |  | | | | **Cognitive orientation exercise** | **Team meetings & care planning** | **Discharge planning** | | | **Increased medical care** | | **Nutritional intervention** | | | **Early intervention** | | **Home visit** | | |
| Bachmann 2010 | Swanson, 1998 | | hip fracture | | | | 0 | 1 | 1 | | | 1 | | 0 | | | 1 | | 1 | | |
| de Morton, 2007 | Slaets 1997 | | medical admission | | | | 0 | 1 | 0 | | | 1 | | 0 | | | 0 | | 0 | | |
| Handoll, 2011 | Graham, 1968 | | hip fracture | | | | 0 | 0 | 0 | | | 0 | | 0 | | | 1 | | 0 | | |
| Heldmann, 2019 | Hagsten, 2004 | | hip fracture | | | | 0 | 0 | 0 | | | 0 | | 0 | | | 0 | | 1 | | |
|  | Asplund 2000 | | medical admission | | | | 0 | 0 | 1 | | | 1 | | 0 | | | 1 | | 0 | | |
|  | Counsell 2000 | | medical admission | | | | 0 | 1 | 1 | | | 1 | | 1 | | | 0 | | 0 | | |
|  | Landefeld 1995 | | medical admission | | | | 0 | 1 | 1 | | | 1 | | 1 | | | 0 | | 0 | | |
|  | Naglie, 2002 | | hip fracture | | | | 0 | 1 | 1 | | | 1 | | 0 | | | 1 | | 1 | | |
|  | Stenvall, 2007 | | hip fracture | | | | 0 | 1 | 0 | | | 1 | | 1 | | | 1 | | 0 | | |
|  | Vidan, 2005 | | hip fracture | | | | 0 | 1 | 0 | | | 1 | | 0 | | | 0 | | 0 | | |
|  | Oldmeadow, 2006 | | hip fracture | | | | 0 | 0 | 0 | | | 0 | | 0 | | | 1 | | 0 | | |
|  | Abizanda, 2011 | | medical admission | | | | 1 | 0 | 0 | | | 0 | | 0 | | | 0 | | 0 | | |
|  | Blanc-Bisson, 2008 | | medical admission | | | | 0 | 0 | 0 | | | 0 | | 1 | | | 1 | | 0 | | |
|  | Jeffs, 2013 | | medical admission | | | | 1 | 0 | 0 | | | 0 | | 0 | | | 0 | | 0 | | |
|  | Barnes, 2012 | | medical admission | | | | 0 | 1 | 1 | | | 1 | | 1 | | | 0 | | 0 | | |
|  | Prestmo, 2015 | | hip fracture | | | | 0 | 1 | 1 | | | 1 | | 1 | | | 1 | | 0 | | |
| Machado, 2020 | Greulich, 2014 | | copd | | | | 0 | 0 | 0 | | | 0 | | 0 | | | 1 | | 0 | | |
|  | Liao, 2015 | | copd | | | | 0 | 0 | 0 | | | 0 | | 1 | | | 0 | | 0 | | |
| Martinez-Vellilla, 2016 | Saltvedt 2002 | | medical admission | | | | 0 | 1 | 1 | | | 1 | | 0 | | | 1 | | 1 | | |
|  | Saltvedt 2006 | | medical admission | | | | 0 | 1 | 1 | | | 1 | | 0 | | | 1 | | 1 | | |
| Peck,2020 | Resnick, 2016 | | orthopaedic trauma | | | | 0 | 0 | 0 | | | 1 | | 0 | | | 0 | | 0 | | |
| Smith,2020 | Marcantonio,2001 | | hip fracture | | | | 0 | 0 | 0 | | | 1 | | 1 | | | 1 | | 0 | | |
| **Total** |  | |  | | | | **2** | **11** | **9** | | | **14** | | **8** | | | **12** | | **5** | | |

COPD = chronic obstructive pulmonary disease
