# Supplementary material for: Effect of inpatient rehabilitation treatment ingredients on functioning, quality of life, length of stay, discharge destination, and mortality among older adults with unplanned admission: an overview review
Source: BMC Geriatr. 2022 Jun 11;22:501. doi: 10.1186/s12877-022-03169-2 (PMC9188066; doi:10.1186/s12877-022-03169-2)

# Supplementary file 5

## List of meta-analyses:

1. Inpatient rehabilitation vs. comparator on functional mobility after inpatient rehabilitation.
2. Inpatient rehabilitation vs. comparator on functional mobility at follow up.
3. Inpatient rehabilitation vs. comparator on activities of daily living after inpatient rehabilitation.
4. Inpatient rehabilitation vs. comparator on activities of daily living at follow up.
5. Inpatient rehabilitation vs. comparator on maintained or improved activities of daily living after inpatient rehabilitation.
6. Inpatient rehabilitation vs. comparator on maintained or improved activities of daily living at follow up.
7. Inpatient rehabilitation vs. comparator on walking speed after inpatient rehabilitation.
8. Inpatient rehabilitation vs. comparator on walking endurance after inpatient rehabilitation.
9. Inpatient rehabilitation vs. comparator on walking endurance pre/post intervention.
10. Inpatient rehabilitation vs. comparator on lower limb strength after inpatient rehabilitation.
11. Inpatient rehabilitation vs. comparator on health-related quality of life after inpatient rehabilitation.
12. Inpatient rehabilitation vs. comparator on health-related quality of life at follow up.
13. Inpatient rehabilitation vs. comparator on health-related quality of life pre post intervention change scores.
14. Inpatient rehabilitation vs. comparator on length of stay.
15. Inpatient rehabilitation vs. comparator on length of stay, by region.
16. Inpatient rehabilitation vs. comparator on discharge destination of home after inpatient rehabilitation.
17. Inpatient rehabilitation vs. comparator on final discharge destination of home.
18. Inpatient rehabilitation vs. comparator on mortality after inpatient rehabilitation.
19. Inpatient rehabilitation vs. comparator on mortality at follow up.

# Inpatient rehabilitation versus comparator on functional mobility after inpatient rehabilitation.

| Systematic Review | Randomized controlled trial | Population | Intervention | Comparator | Outcome measure |
| --- | --- | --- | --- | --- | --- |
| Handoll 2011 | Mitchell 2001 | Hip fracture | Strengthening exercise; Repeated practice functions (+/-increasing demands) | Usual care (UK). 20 minutes physiotherapy per weekday. | Elderly Mobility Scale |
| Heldmann 2019 | Prestmo 2015 | Hip fracture | Repeated practice activities (+/- increasing demands); Goals and planning; Team meetings & care planning; Discharge planning; Increased medical care; Nutritional intervention; Early intervention | Usual care (Norway). Physiotherapy according to guidelines. No occupational therapists. | Timed Up and Go |
| Heldmann 2019 | Kimmel 2016 | Hip fracture | Repeated exercise rehabilitation | Usual care (Australia). Daily physiotherapy. | Timed Up and Go |
| Peck 2020 | Resnick 2016 | Orthopaedic trauma | Repeated practice activities (+/- increasing demands); Goals and planning; Feedback and monitoring; Shaping knowledge; Antecedents; Increased medical care | Usual care (USA), plus education | Physical Performance and Mobility Examination |
| Peiris 2018 | Peiris 2013 | Medical admission | Repeated exercise rehabilitation | Usual care (Australia). Physiotherapy weekdays. | Timed Up and Go |


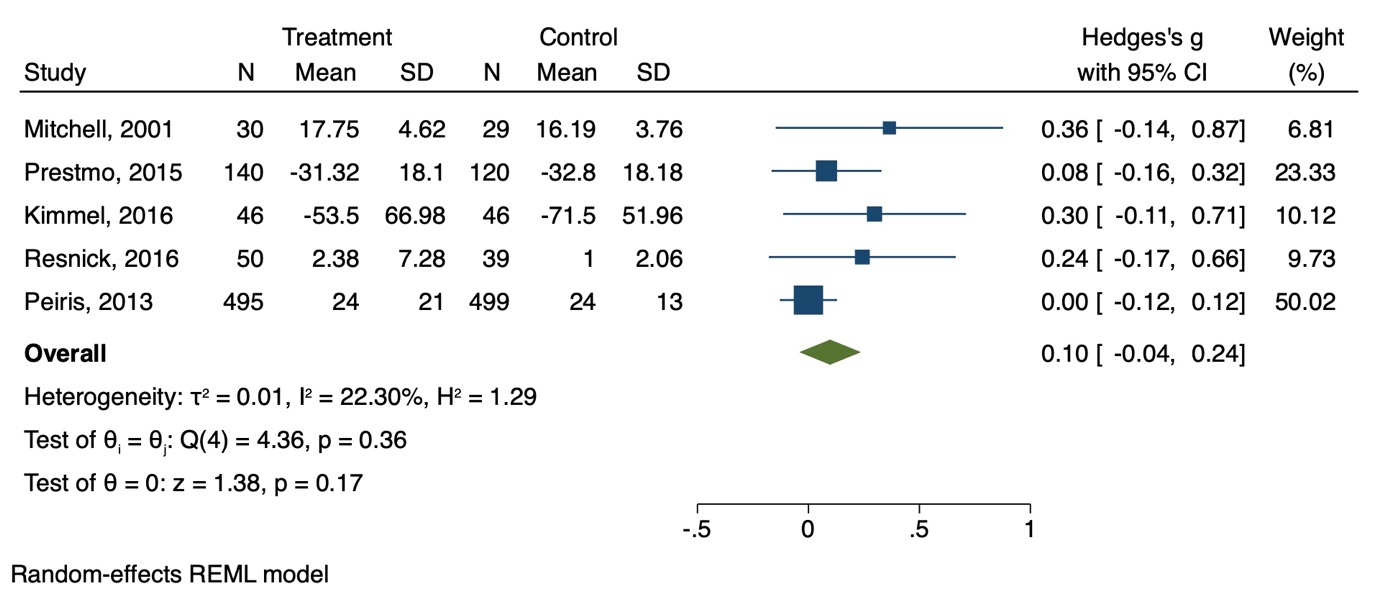


With removal of RCT from systematic review of critically low quality


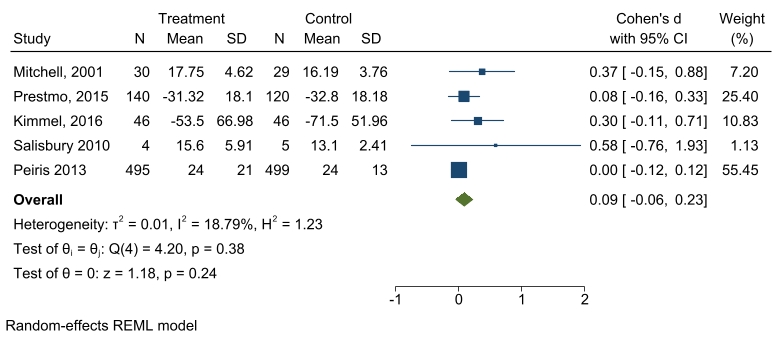


# inpatient rehabilitation versus comparator on functional mobility at follow up

#

| Systematic Review | Randomized controlled trial | Population | Intervention | Comparator | Outcome measure | Length of follow up |
| --- | --- | --- | --- | --- | --- | --- |
| Handoll 2011 | Mitchell 2001 | Hip fracture | Strengthening exercise; Repeated practice functions (+/-increasing demands) | Usual care (UK). 20 minutes physiotherapy per weekday. | Elderly Mobility Scale | 16 weeks |
| Heldmann 2019 | Prestmo 2015 | Hip fracture | Repeated practice activities (+/- increasing demands); Goals and planning; Team meetings & care planning; Discharge planning; Increased medical care; Nutritional intervention; Early intervention | Usual care (Norway). Physiotherapy according to guidelines. No occupational therapists. | Timed Up and Go | 12 months |


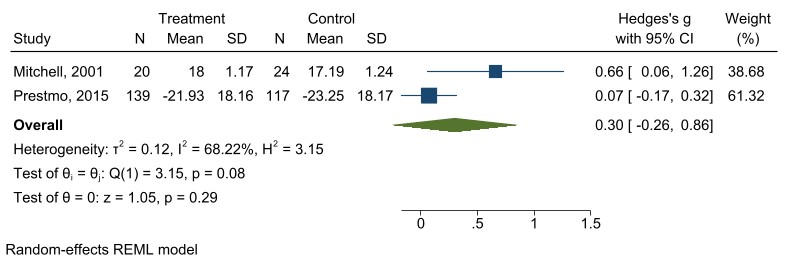


# Inpatient rehabilitation versus comparator on activities of daily living after inpatient rehabilitation.

| Systematic review | Randomized controlled trial | Population | Intervention | Comparator | Outcome measure |
| --- | --- | --- | --- | --- | --- |
| Handoll 2011 | Mitchell 2001 | Hip fracture | Strengthening exercise; Repeated practice functions (+/-increasing demands) | Usual care (UK). 20 minutes physiotherapy per weekday. | Barthel Index |
| Heldmann 2019 | Brown 2016 | Medical admission | Endurance exercise; Goals and planning; Feedback and monitoring | Usual care (USA). Allied health if referred. | modified Katz Activities of Daily Living Index |
| Heldmann 2019 | Abizanda 2011 | Medical admission | Repeated practice activities (+/- increasing demands); Goals and planning; Shaping knowledge; Cognitive orientation exercise | Usual care (Spain). Includes physiotherapy. | Barthel Index |
| Heldmann 2019 | Prestmo 2015 | Hip fracture | Repeated practice activities (+/- increasing demands); Goals and planning; Team meetings & care planning; Discharge planning; Increased medical care; Nutritional intervention; Early intervention | Usual care (Norway). Physiotherapy according to guidelines. No occupational therapists. | Barthel Index |
| Machado 2020 | Lopez-Lopez 2018 (1) | COPD | Energy applied to soft tissue | Usual care (Spain). Medical care alone. | London Chest Activity of Daily Living Score |
| Maranesi 2020 | Lopez-Lopez 2018 (2) | COPD | Exercise scheduling for strengthening | Usual care (Spain). Medical care alone. | London Chest Activity of Daily Living Score |
| Machado 2020 | Lopez-Lopez 2019a (1) | COPD | Endurance exercise; Energy applied to soft tissue; Goals and planning; Feedback and monitoring | Usual care (Spain). Medical care alone. | Functional Independence Measure |
| Machado 2020 | Lopez-Lopez 2019a (2) | COPD | Endurance exercise; Energy applied to soft tissue; Goals and planning; Feedback and monitoring | Usual care (Spain). Medical care alone. | Functional Independence Measure |
| Martinez-Velilla 2016 | Tibaek 2014 | Older adult | Strengthening exercise | Usual care (Denmark). Physiotherapy. | Barthel Index |
| Scrivener 2015 | Said 2012 | Older adult | Endurance exercise; Repeated practice activities (+/- increasing demands) | Usual care (Australia). Physiotherapy 1-2 sessions on weekdays. | Barthel Index |
| Bachmann 2010 | Swanson 1998 | Hip fracture | Repeated exercise rehabilitation; Team meetings & care planning; Discharge planning; Increased medical care; Early intervention; Home visit | Usual care (Australia). Physiotherapy. Occupational therapy on referral. | Modified Barthel Index |
| Smith 2020b | Lenze 2012 | Medical admission | Repeated exercise rehabilitation; Goals and planning; Feedback and monitoring | Usual care (USA). Physiotherapy and occupational therapy. | Barthel Index |
| Smith 2020b | Counsell 2020 | Medical admission | Repeated practice activities (+/- increasing demands); Antecedents; Team meetings & care planning; Discharge planning; Increased medical care; Nutritional intervention | Usual care (USA). Medical care. | Independent Activities of Daily Living |
| Peck 2020 | Resnick 2016 | Orthopaedic trauma | Repeated practice activities (+/- increasing demands); Goals and planning; Feedback and monitoring; Shaping knowledge; Antecedents; Increased medical care | Usual care (USA), plus education | Barthel Index |
| Smith 2020b | Timmer 2019 | Medical admission | Repeated practice activities (+/- increasing demands); Goals and planning; Feedback and monitoring; Social support; Shaping knowledge; Natural consequences | Usual care (Australia) plus a brief activity pacing education. Physiotherapy and occupational therapy. | Functional Independence Measure |
| Peiris 2018 | Peiris 2013 | Medical admission | Repeated exercise rehabilitation | Usual care (Australia). Physiotherapy weekdays. | Functional Independence Measure |
| Yasmeen 2020 | Louie 2012 | Hip fracture | Repeated practice activities (+/- increasing demands); Goals and planning; Feedback and monitoring; Social support; Shaping knowledge; Natural consequences; Comparison of behavior | Usual care (Hong Kong, China). Hip fracture protocol including activities of daily living training. | Functional Independence Measure (motor scores) |

COPD = chronic obstructive pulmonary disease


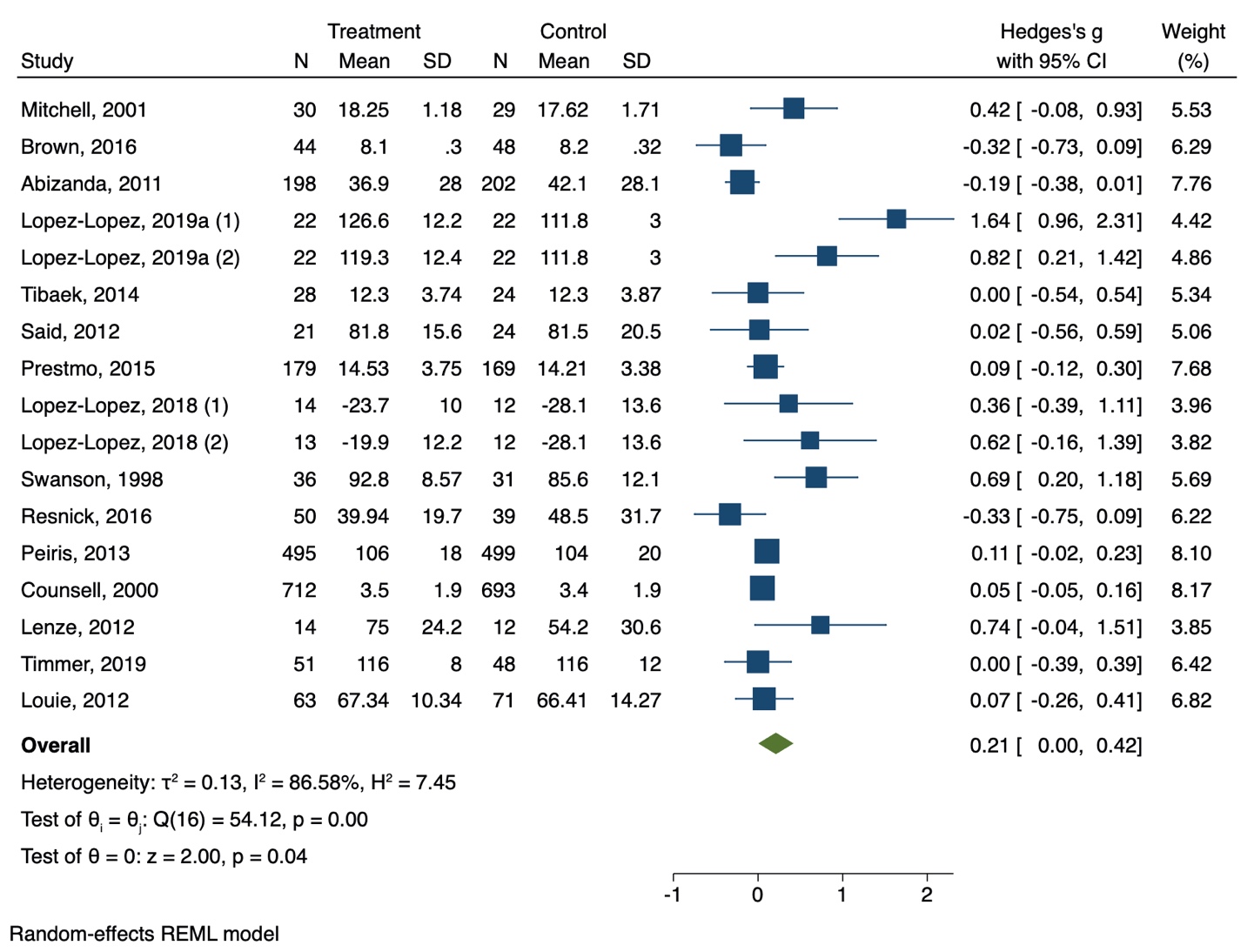


With removal of RCT from systematic review of low or critically low quality


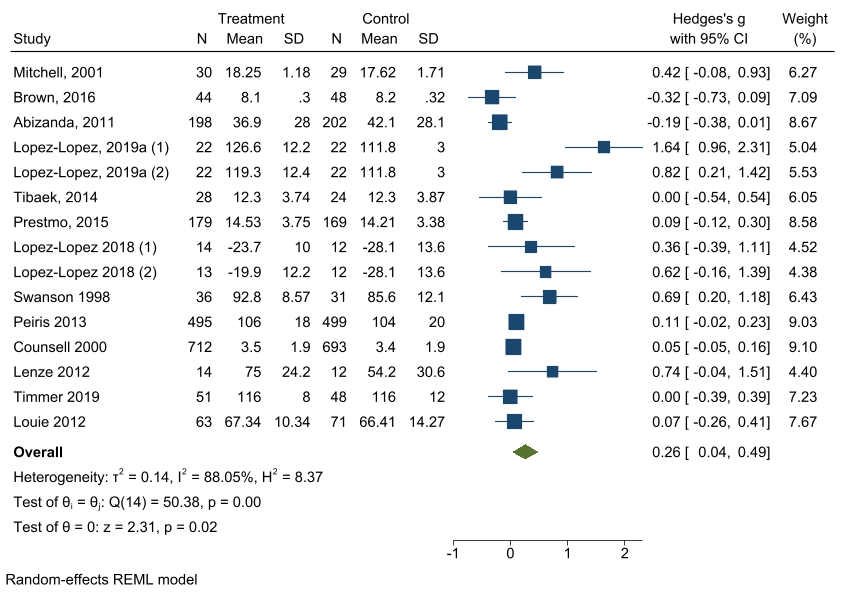


# Inpatient rehabilitation versus comparator on activities of daily living at follow up

| Systematic review | Randomized controlled trial | Population | Intervention | Comparator | Outcome measure | Length of follow up |
| --- | --- | --- | --- | --- | --- | --- |
| Scrivener 2015 | Said 2012 | Older adult | Endurance exercise; Repeated practice activities (+/- increasing demands) | Usual care (Australia). Physiotherapy 1-2 sessions on weekdays. | Barthel Index | 3 months |
| Heldmann 2019 | Prestmo 2015 | Hip fracture | Repeated practice activities (+/- increasing demands); Goals and planning; Team meetings & care planning; Discharge planning; Increased medical care; Nutritional intervention; Early intervention | Usual care (Norway). Physiotherapy according to guidelines. No occupational therapists. | Barthel Index | 12 months |
| Peiris 2018 | Peiris 2013 | medical admission | Repeated exercise rehabilitation | Usual care (Australia). Physiotherapy weekdays. | Functional Independence Measure score | 12 months |
| Peck 2020 | Resnick 2016 | orthopaedic trauma | Repeated practice activities (+/- increasing demands); Goals and planning; Feedback and monitoring; Shaping knowledge; Antecedents; Increased medical care | Usual care (USA), plus education | Barthel Index | 1 month |
| Handoll 2011 | Mitchell 2001 | Hip fracture | Strengthening exercise; Repeated practice functions (+/-increasing demands) | Usual care (UK). 20 minutes physiotherapy per weekday. | Barthel Index (disability) 20-point scale | 16 weeks |

#
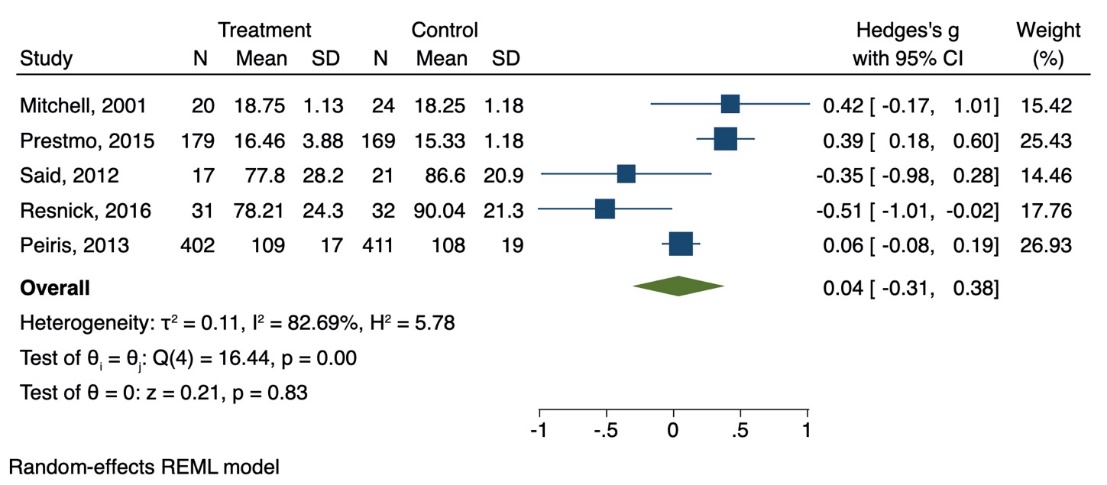


With removal of RCT from systematic review of low or critically low quality


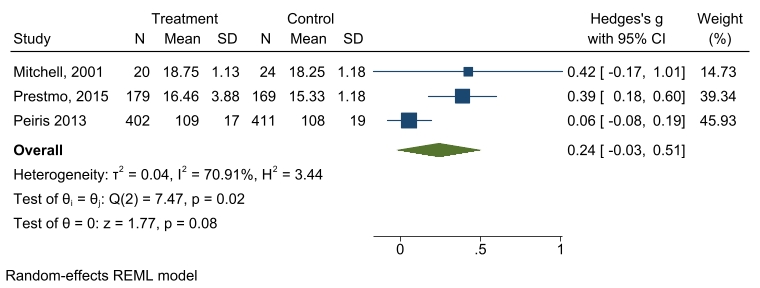


# Inpatient rehabilitation versus comparator on improved activities of daily living after inpatient rehabilitation.

| Systematic review | Randomized controlled trial | Population | Intervention | Comparator | Outcome measure |
| --- | --- | --- | --- | --- | --- |
| de Morton 2007 | Slaets 1997 | Medical admission | Repeated exercise rehabilitation; Team meetings & care planning; Increased medical care | Usual care (The Netherlands). Services provided by physicians and nurses. | SIVIS (Health Care Information Center Foundation) dependency scale |
| Smith 2020b | Counsell 2000 | Medical admission | Repeated practice activities (+/- increasing demands); Antecedents; Team meetings & care planning; Discharge planning; Increased medical care; Nutritional intervention | Usual care (USA). Medical care. | modified Katz Activities of Daily Living Index |
| Smith 2020b | Landefeld 1995 | Medical admission | Repeated practice activities (+/- increasing demands); Antecedents; Team meetings & care planning; Discharge planning; Increased medical care; Nutritional intervention | Usual care (USA). Physiotherapy. | Katz Activities of Daily Living Index |
| Heldmann 2019 | Barnes 2012 | Medical admission | Repeated practice activities (+/- increasing demands); Antecedents; Team meetings & care planning; Discharge planning; Increased medical care; Nutritional intervention | Usual care (USA). Physiotherapy if referred. | Katz Activities of Daily Living Index |
| Heldmann 2019 | Stenvall 2007 | Hip fracture | Endurance exercise; Repeated practice activities (+/- increasing demands); Goals and planning; Shaping knowledge; Team meetings & care planning; Increased medical care; Nutritional intervention; Early intervention | Usual care (Sweden). Exercise rehabilitation with daily physiotherapy and occupational therapy as needed. | Less dependent, based on Katz Index |
| Peiris 2018 | Peiris 2013 | Medical admission | Repeated exercise rehabilitation | Usual care (Australia). Physiotherapy weekdays. | Functional Independence Measure (minimally clinically important difference) |


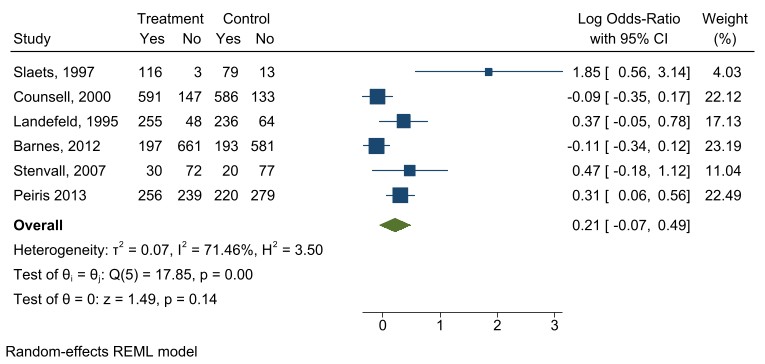


# Inpatient rehabilitation versus comparator on improved activities of daily LIVING AT follow up

| Systematic review | Randomized controlled trial | Population | Intervention | Comparator | Outcome measure | Length of follow up |
| --- | --- | --- | --- | --- | --- | --- |
| Heldmann 2019 | Stenvall 2007 | Hip fracture | Endurance exercise; Repeated practice activities (+/- increasing demands); Goals and planning; Shaping knowledge; Team meetings & care planning; Increased medical care; Nutritional intervention; Early intervention | Usual care (Sweden). Exercise rehabilitation with daily physiotherapy and occupational therapy as needed. | Less dependent, based on Katz Index | 12 months |
| Peiris 2018 | Peiris 2013 | Medical admission | Repeated exercise rehabilitation | Usual care (Australia). Physiotherapy weekdays. | Functional Independence Measure (minimally clinically important difference) | 12 months |


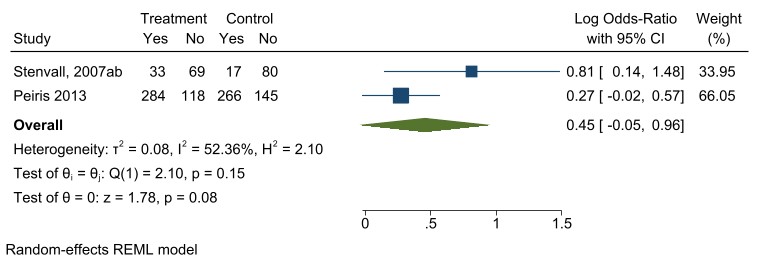


# Inpatient rehabilitation versus comparator on walking speed after inpatient rehabilitation.

| Systematic review | Randomized controlled trial | Population | Intervention | Comparator | Outcome measure |
| --- | --- | --- | --- | --- | --- |
| Handoll 2011 | Baker 1991 | Hip fracture | Endurance exercise; Antecedents | Usual care (Australia) including conventional gait training with ambulatory aids. | metres/minute |
| Handoll 2011 | Mitchell 2001 | Hip fracture | Strengthening exercise; Repeated practice functions (+/-increasing demands) | Usual care (UK). 20 minutes physiotherapy per weekday. | metres/second |
| Martinez-Velilla 2016 | Tibaek 2014 | Older adult | Strengthening exercise | Usual care (Denmark) | 10 metre walk test (seconds) |
| Peiris 2018 | Peiris 2013 | Medical admission | Repeated exercise rehabilitation | Usual care (Australia). Physiotherapy weekdays. | 10-metre walk test (metres/seconds) |
| Smith 2020b | Lenze 2012 | Medical admission | Repeated exercise rehabilitation; Goals and planning; Feedback and monitoring | Usual care (USA). Physiotherapy and occupational therapy. | Gait Speed (metres/seconds) |


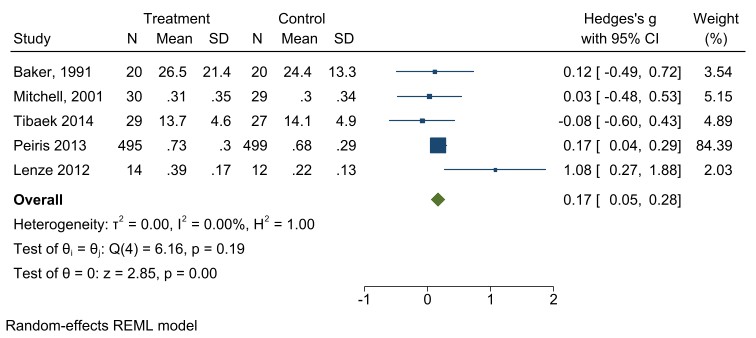


# Inpatient rehabilitation versus comparator on walking endurance after inpatient rehabilitation.

| Systematic review | Randomized controlled trial | Population | Intervention | Comparator | Outcome measure |
| --- | --- | --- | --- | --- | --- |
| Heldmann 2019 | Oldmeadow 2006 | Hip fracture | Early intervention | Delayed assisted ambulation to post op day 3 or 4 | Mean walking distance at 7 days (metres) |
| Machado 2020 | Greulich 2014 | COPD | Energy applied to soft tissue; Early intervention | Usual care (Germany). 20 minutes physiotherapy. | 6-minute walk test (metres) |
| Machado 2020 | Torres-Sanchez 2017 | COPD | Endurance exercise; Shaping knowledge | Usual care (Spain). Medical care alone. | Steps per day |
| Machado 2020 | He 2015 | COPD | Strengthening exercise; Endurance exercise; Energy applied to soft tissue; Breathing related exercise/training; Shaping knowledge; Natural consequences | Usual care (China) | 6-minute walk test (metres) |
| Machado 2020 | Kirsten 1998 | COPD | Endurance exercise | Usual care (Germany). No regular exercise, walking assessments on 4 days. | 6-minute walk test (metres) |
| Smith 2020b | Lenze 2012 | Medical admission | Repeated exercise rehabilitation; Goals and planning; Feedback and monitoring | Usual care (USA). Physiotherapy and occupational therapy. | 6-min walk (feet) |


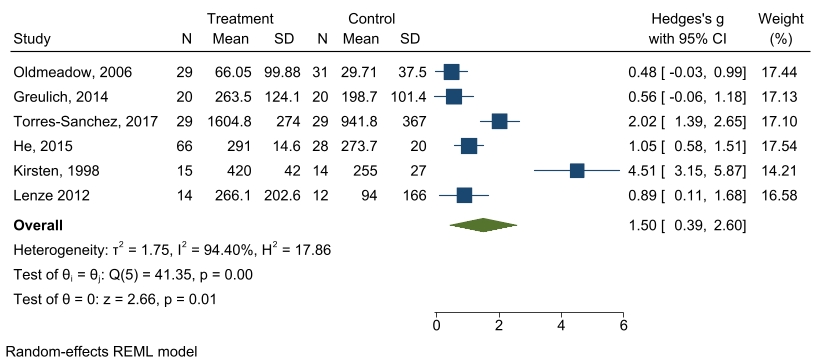


# Inpatient rehabilitation versus comparator on walking endurance pre/post intervention

| Systematic review | Randomized controlled trial | Population | Intervention | Comparator | Outcome measure |
| --- | --- | --- | --- | --- | --- |
| Machado 2020 | Torres-Sanchez 2016 | COPD | Endurance exercise; Shaping knowledge | Usual care (Spain). Medical care alone. | 2-minutes step in place test (number of repetitions) |
| Machado 2020 | Borges 2014 | COPD | Repeated practice functions (+/-increasing demands); Shaping knowledge; Comparison of behavior | Usual care (Brazil). Chest physiotherapy and advice re physical activity. | Change in 6-minute walk test |
| Machado 2020 | Liao 2015 | COPD | Endurance exercise; Breathing related exercise/ training; Shaping knowledge; Natural consequences; Nutritional intervention | Usual care (Taiwan). Health education, monitoring of vital signs and symptoms, assessing nutritional status, and nasal oxygen therapy | Change in 6-minute walk test |


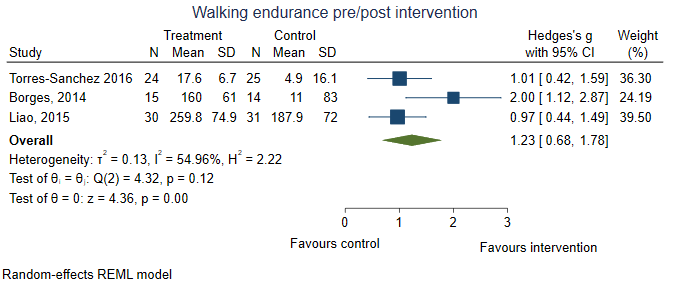


# Inpatient rehabilitation versus comparator on lower limb strength after inpatient rehabilitation.

| Systematic review | Randomized controlled trial | Population | Intervention | Comparator | Outcome measure |
| --- | --- | --- | --- | --- | --- |
| Handoll 2011 | Mitchell 2001 | Hip fracture | Strengthening exercise; Repeated practice functions (+/-increasing demands) | Usual care (UK). 20 minutes physiotherapy per weekday. | Nottingham Power Rig leg extensor power (watts) |
| Machado 2020 | Greulich 2014 | COPD | Energy applied to soft tissue; Early intervention | Usual care (Germany). 20 minutes physiotherapy. | Chair rising test |
| Machado 2020 | Torres-Sanchez 2017 | COPD | Endurance exercise; Shaping knowledge | Usual care (Spain). Medical care alone. | 30-second sit to stand (number of repetitions) |
| Machado 2020 | Lopez-Lopez 2019a (1) | COPD | Endurance exercise; Energy applied to soft tissue; Goals and planning; Feedback and monitoring | Usual care (Spain). Medical care alone. | 5-times sit to stand |
| Machado 2020 | Lopez-Lopez 2019a (2) | COPD | Endurance exercise; Energy applied to soft tissue; Goals and planning; Feedback and monitoring | Usual care (Spain). Medical care alone. | 5-times sit to stand |
| Machado 2020 | Lopez-Lopez 2018 (1) | COPD | Energy applied to soft tissue | Usual care (Spain). Medical care alone. | 5-times sit to stand |
| Maranesi 2020 | Lopez-Lopez 2018 (2) | COPD | Exercise scheduling for strengthening | Usual care (Spain). Medical care alone. | 5-times sit to stand |


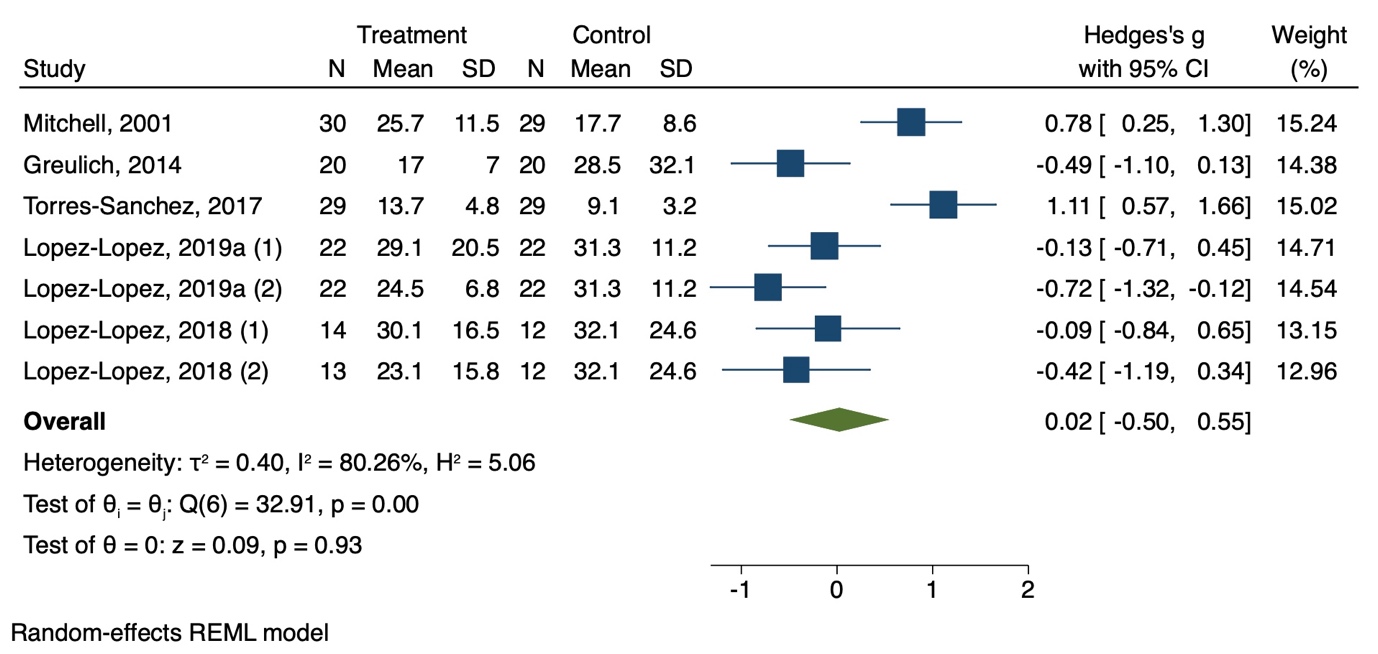


# Inpatient rehabilitation versus comparator on HEALTH-RELATED quality of life after inpatient rehabilitation.

| Systematic review | Randomized controlled trial | Population | Intervention | Comparator | Outcome measure |
| --- | --- | --- | --- | --- | --- |
| Heldmann 2019 | Prestmo 2015 | Hip fracture | Repeated practice activities (+/- increasing demands); Goals and planning; Team meetings & care planning; Discharge planning; Increased medical care; Nutritional intervention; Early intervention | Usual care (Norway). Physiotherapy according to guidelines. No occupational therapists. | EQ-5D |
| Machado 2020 | Greulich 2014 | COPD | Energy applied to soft tissue; Early intervention | Usual care (Germany). 20 minutes physiotherapy. | St George’s Respiratory Questionnaire |
| Machado 2020 | Torres-Sanchez 2018 (1) | COPD | Breathing related exercise/ training; Repeated practice functions (+/-increasing demands); Feedback and monitoring | Usual care (Spain). Medical care alone. | EQ-5D |
| Machado 2020 | Torres-Sanchez 2018 (2) | COPD | Exercise scheduling for strengthening; Feedback and monitoring | Usual care (Spain). Medical care alone. | EQ-5D |
| Machado 2020 | Lopez-Lopez 2019a (1) | COPD | Endurance exercise; Energy applied to soft tissue; Goals and planning; Feedback and monitoring | Usual care (Spain). Medical care alone. | EQ-5D |
| Machado 2020 | Lopez-Lopez 2019a (2) | COPD | Endurance exercise; Energy applied to soft tissue; Goals and planning; Feedback and monitoring | Usual care (Spain). Medical care alone. | EQ-5D |
| Peiris 2018 | Peiris 2013 | Medical admission | Repeated exercise rehabilitation | Usual care (Australia). Physiotherapy weekdays. | EQ-5D |

EQ-5D = EuroQol 5 dimensions


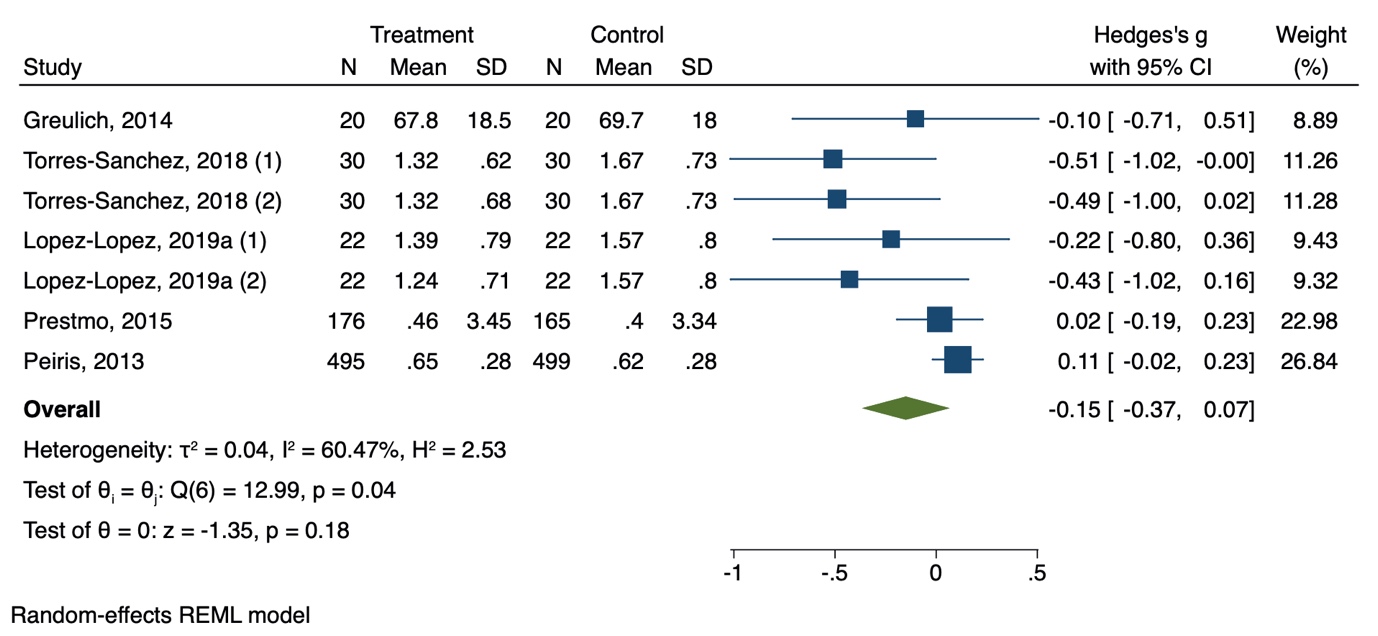


# Inpatient rehabilitation versus comparator on HEALTH-RELATED quality of life at follow up

| Systematic review | Randomized controlled trial | Population | Intervention | Comparator | Outcome measure | Length of follow up |
| --- | --- | --- | --- | --- | --- | --- |
| Heldmann 2019 | Prestmo 2015 | Hip fracture | Repeated practice activities (+/- increasing demands); Goals and planning; Team meetings & care planning; Discharge planning; Increased medical care; Nutritional intervention; Early intervention | Usual care (Norway). Physiotherapy according to guidelines. No occupational therapists. | EQ-5D | 12 months |
| Peiris 2018 | Peiris 2013 | Medical admission | Repeated exercise rehabilitation | Usual care (Australia). Physiotherapy weekdays. | EQ-5D | 12 months |

EQ-5D = EuroQol 5 dimensions


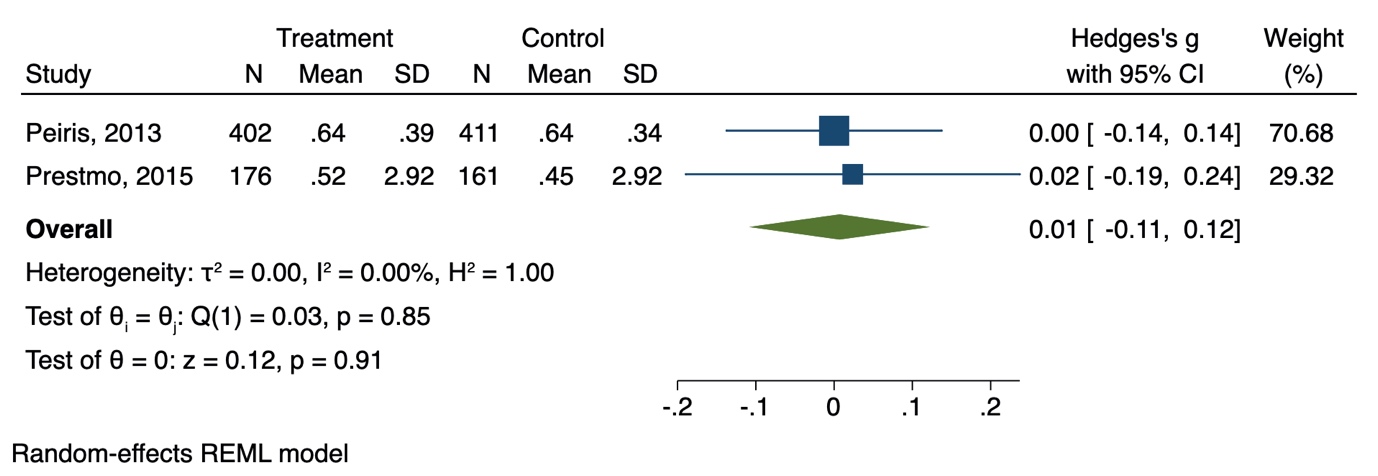


# Inpatient rehabilitation versus comparator on HEALTH-RELATED quality of life PRE-POST intervention change scores

| Systematic review | Randomized controlled trial | Population | Intervention | Comparator | Outcome measure |
| --- | --- | --- | --- | --- | --- |
| Machado 2020 | Borges 2014 | COPD | Repeated practice functions (+/-increasing demands); Shaping knowledge; Comparison of behavior | Usual care (Brazil). Chest physiotherapy and advice re physical activity. | St George’s Respiratory Questionnaire |
| Machado 2020 | Torres-Sanchez 2016 | COPD | Exercise scheduling for strengthening; Breathing related exercise/ training; Repeated practice functions (+/-increasing demands); Feedback and monitoring; Shaping knowledge | Usual care (Spain). Medical care alone. | EQ-5D |

EQ-5D = EuroQol 5 dimensions

# Inpatient rehabilitation versus comparator on length of stay

| Systematic review | Randomized controlled trial | Population | Intervention | Comparator |
| --- | --- | --- | --- | --- |
| de Morton 2007 | Slaets 1997 | Medical admission | Repeated exercise rehabilitation; Team meetings & care planning; Increased medical care | Usual care (The Netherlands). Services provided by physicians and nurses. |
| Handoll 2011 | Karumo 1977 | Hip fracture | Repeated practice activities (+/- increasing demands); Repeated exercise rehabilitation | Usual care (Finland) |
| Handoll 2011 | Lauridsen 2002 | Hip fracture | Repeated practice functions (+/- increasing demands); Repeated practice activities (+/- increasing demands) | Usual care (Denmark). Physiotherapy 15-30 minutes per weekday. |
| Handoll 2011 | Baker 1991 | Hip fracture | Endurance exercise; Antecedents | Usual care (Australia) including conventional gait training with ambulatory aids. |
| Heldmann 2019 | Asplund 2000 | Medical admission | Early intervention; Discharge planning; Increased medical care | Usual care (Sweden). Physiotherapy and occupational therapy not routinely available. |
| Smith 2020b | Counsell 2000 | Medical admission | Repeated practice activities (+/- increasing demands); Antecedents; Team meetings & care planning; Discharge planning; Increased medical care; Nutritional intervention | Usual care (USA). Medical care. |
| Heldmann 2019 | Jones 2006 | Medical admission | Exercise scheduling for strengthening; Repeated practice activities (+/- increasing demands) | Usual care (Australia). Standard physiotherapy. |
| Smith 2020b | Landefeld 1995 | Medical admission | Repeated practice activities (+/- increasing demands); Antecedents; Team meetings & care planning; Discharge planning; Increased medical care; Nutritional intervention | Usual care (USA). Physiotherapy. |
| Heldmann 2019 | Naglie 2002 | Hip fracture | Repeated practice activities (+/- increasing demands); Feedback and monitoring; Shaping knowledge; Team meetings & care planning; Discharge planning; Increased medical care; Early intervention; Home visit | Usual care (Canada). Physiotherapy if referred. Occupational therapy rarely. |
| Heldmann 2019 | Stenvall 2007 | Hip fracture | Endurance exercise; Repeated practice activities (+/- increasing demands); Goals and planning; Shaping knowledge; Team meetings & care planning; Increased medical care; Nutritional intervention; Early intervention | Usual care (Sweden). Exercise rehabilitation with daily physiotherapy and occupational therapy as needed. |
| Heldmann 2019 | Vidan 2005 | Hip fracture | Repeated exercise rehabilitation; Antecedents; Team meetings & care planning; Increased medical care | Usual care (Spain) Physiotherapy. Occupational therapy not available. |
| Heldmann 2019 | Oldmeadow 2006 | Hip fracture | Early intervention | Delayed assisted ambulation to post op day 3 or 4 |
| Heldmann 2019 | Brown 2016 | Medical admission | Endurance exercise; Goals and planning; Feedback and monitoring | Usual care (USA). Allied health if referred. |
| Heldmann 2019 | Jeffs 2013 | Medical admission | Exercise scheduling for strengthening; Feedback and monitoring; Shaping knowledge; Cognitive orientation exercise | Usual care (Australia). Allied health if referred. |
| Heldmann 2019 | Kimmel 2016 | Hip fracture | Repeated exercise rehabilitation | Usual care (Australia). Daily physiotherapy. |
| Heldmann 2019 | Prestmo 2015 | Hip fracture | Repeated practice activities (+/- increasing demands); Goals and planning; Team meetings & care planning; Discharge planning; Increased medical care; Nutritional intervention; Early intervention | Usual care (Norway). Physiotherapy according to guidelines. No occupational therapists. |
| Machado 2020 | Greulich 2014 | COPD | Energy applied to soft tissue; Early intervention | Usual care (Germany). 20 minutes physiotherapy. |
| Machado 2020 | Torres-Sanchez 2017 | COPD | Endurance exercise; Shaping knowledge | Usual care (Spain). Medical care alone. |
| Machado 2020 | Torres-Sanchez 2016 | COPD | Exercise scheduling for strengthening; Breathing related exercise/ training; Repeated practice functions (+/-increasing demands); Feedback and monitoring; Shaping knowledge | Usual care (Spain). Medical care alone. |
| Machado 2020 | Lopez-Lopez 2019a (1) | COPD | Endurance exercise; Energy applied to soft tissue; Goals and planning; Feedback and monitoring | Usual care (Spain). Medical care alone. |
| Machado 2020 | Lopez-Lopez 2019a (2) | COPD | Endurance exercise; Energy applied to soft tissue; Goals and planning; Feedback and monitoring | Usual care (Spain). Medical care alone. |
| Machado 2020 | Lopez-Lopez 2019b (1) | COPD | Energy applied to soft tissue; Repeated practice functions (+/-increasing demands) | Usual care (Spain). Medical care alone. |
| Machado 2020 | Lopez-Lopez 2019b (2) | COPD | Energy applied to soft tissue; Breathing related exercise/ training; Repeated practice functions (+/-increasing demands); Goals and planning; Feedback and monitoring; Shaping knowledge; Natural consequences | Usual care (Spain). Medical care alone. |
| Machado 2020 | Torres-Sanchez 2018 (1) | COPD | Breathing related exercise/ training; Repeated practice functions (+/-increasing demands); Feedback and monitoring | Usual care (Spain). Medical care alone. |
| Machado 2020 | Torres-Sanchez 2018 (2) | COPD | Exercise scheduling for strengthening; Feedback and monitoring | Usual care (Spain). Medical care alone. |
| Machado 2020 | Lopez-Lopez 2018 (1) | COPD | Energy applied to soft tissue | Usual care (Spain). Medical care alone. |
| Machado 2020 | Lopez-Lopez 2018 (2) | COPD | Exercise scheduling for strengthening | Usual care (Spain). Medical care alone. |
| Scrivener 2015 | Said 2012 | Older adult | Endurance exercise; Repeated practice activities (+/- increasing demands) | Usual care (Australia). Physiotherapy 1-2 sessions on weekdays. |
| Smith 2020 | Marcantonio 2001 | Hip fracture | Antecedents; Increased medical care; Nutritional intervention; Early intervention | Usual care (USA). Medical care. |
| Bachmann 2010 | Swanson 1998 | Hip fracture | Repeated exercise rehabilitation; Team meetings & care planning; Discharge planning; Increased medical care; Early intervention; Home visit | Usual care (Australia). Physiotherapy. Occupational therapy on referral. |
| Peiris 2018 | Peiris 2013 | Medical admission | Repeated exercise rehabilitation | Usual care (Australia). Physiotherapy weekdays. |
| Smith 2020b | Timmer 2019 | Medical admission | Repeated practice activities (+/- increasing demands); Goals and planning; Feedback and monitoring; Social support; Shaping knowledge; Natural consequences | Usual care (Australia) plus a brief activity pacing education. Physiotherapy and occupational therapy. |


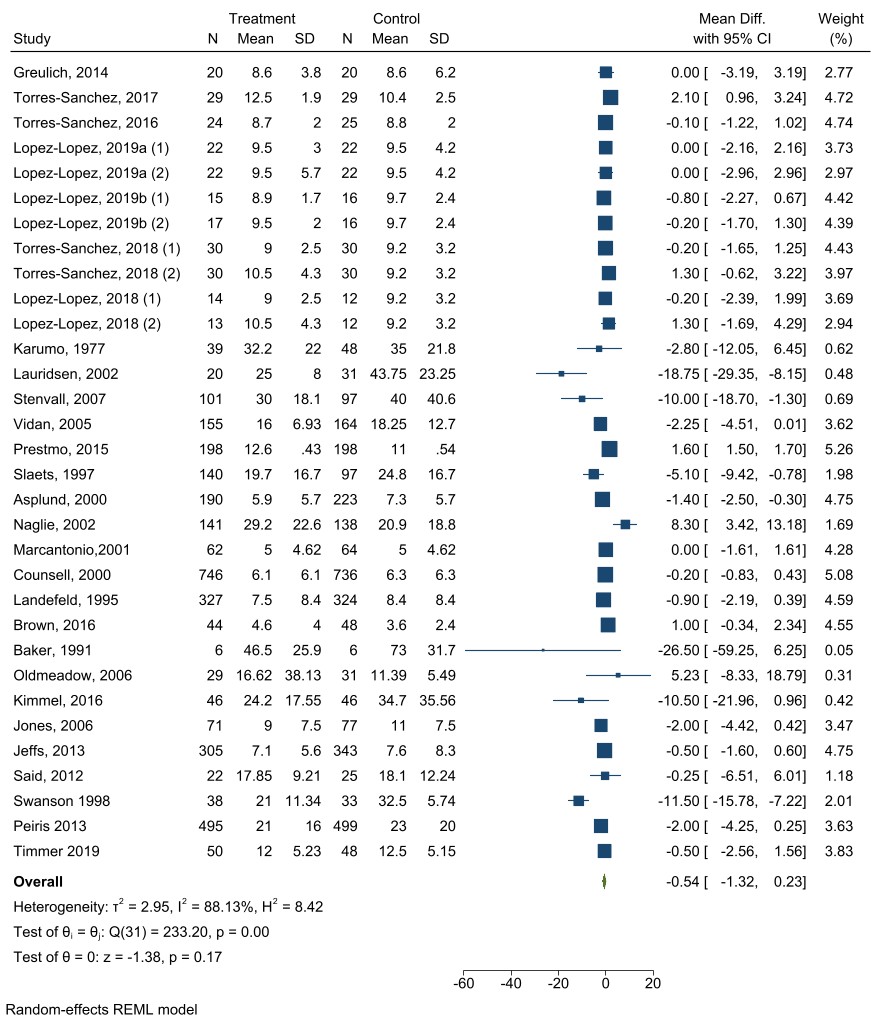


With removal of RCT from systematic review of low quality


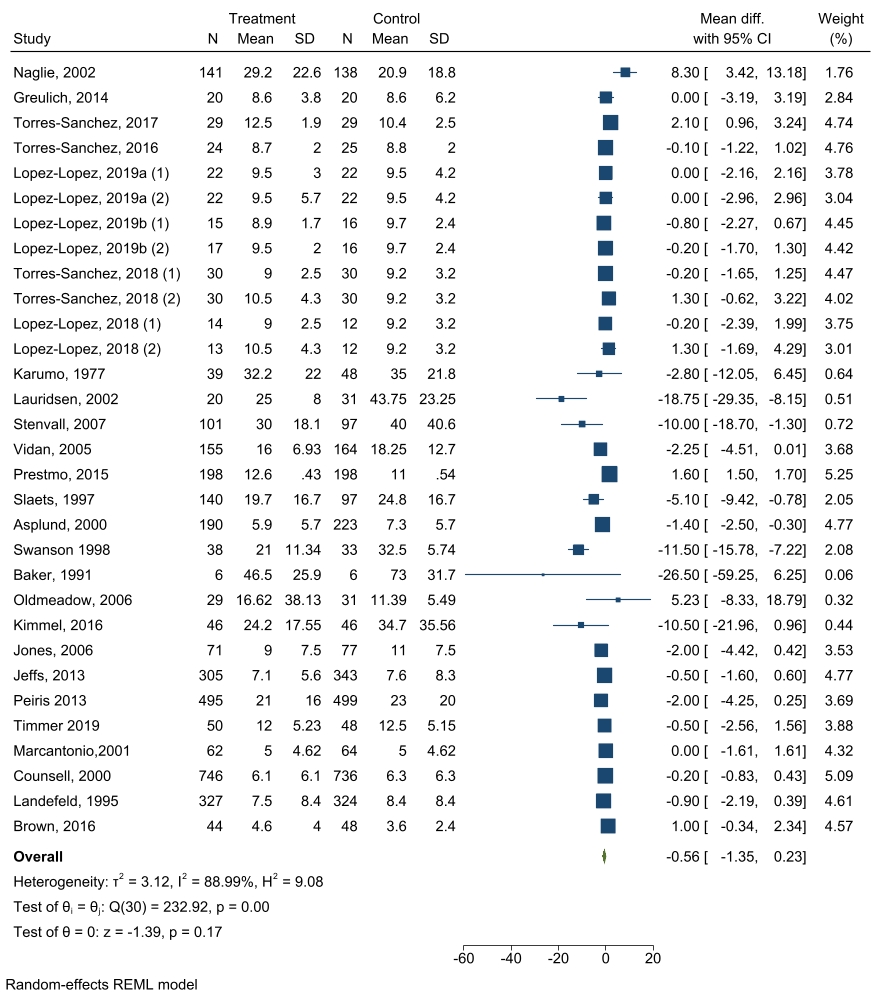


By region
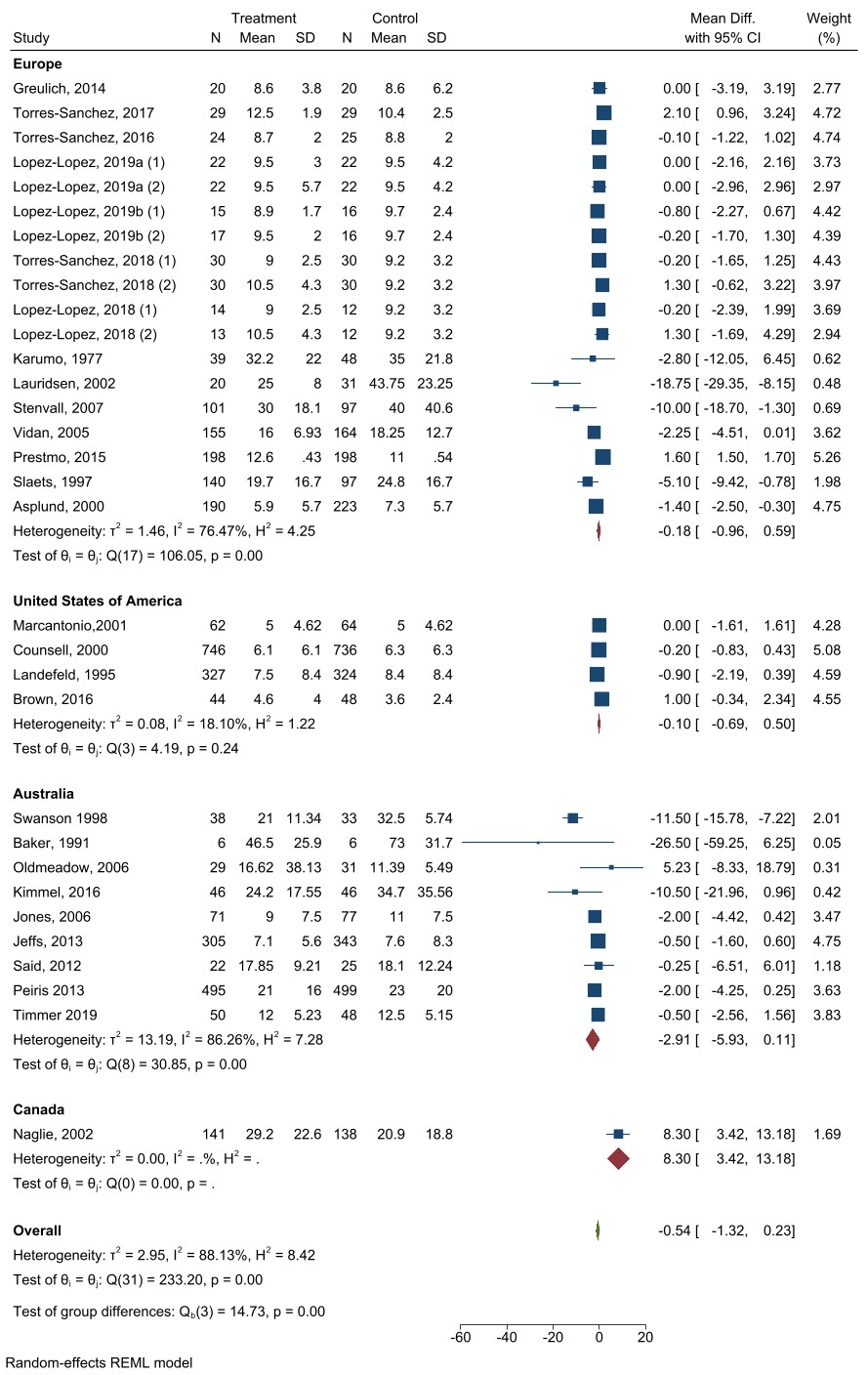


With removal of RCT from systematic review of low quality

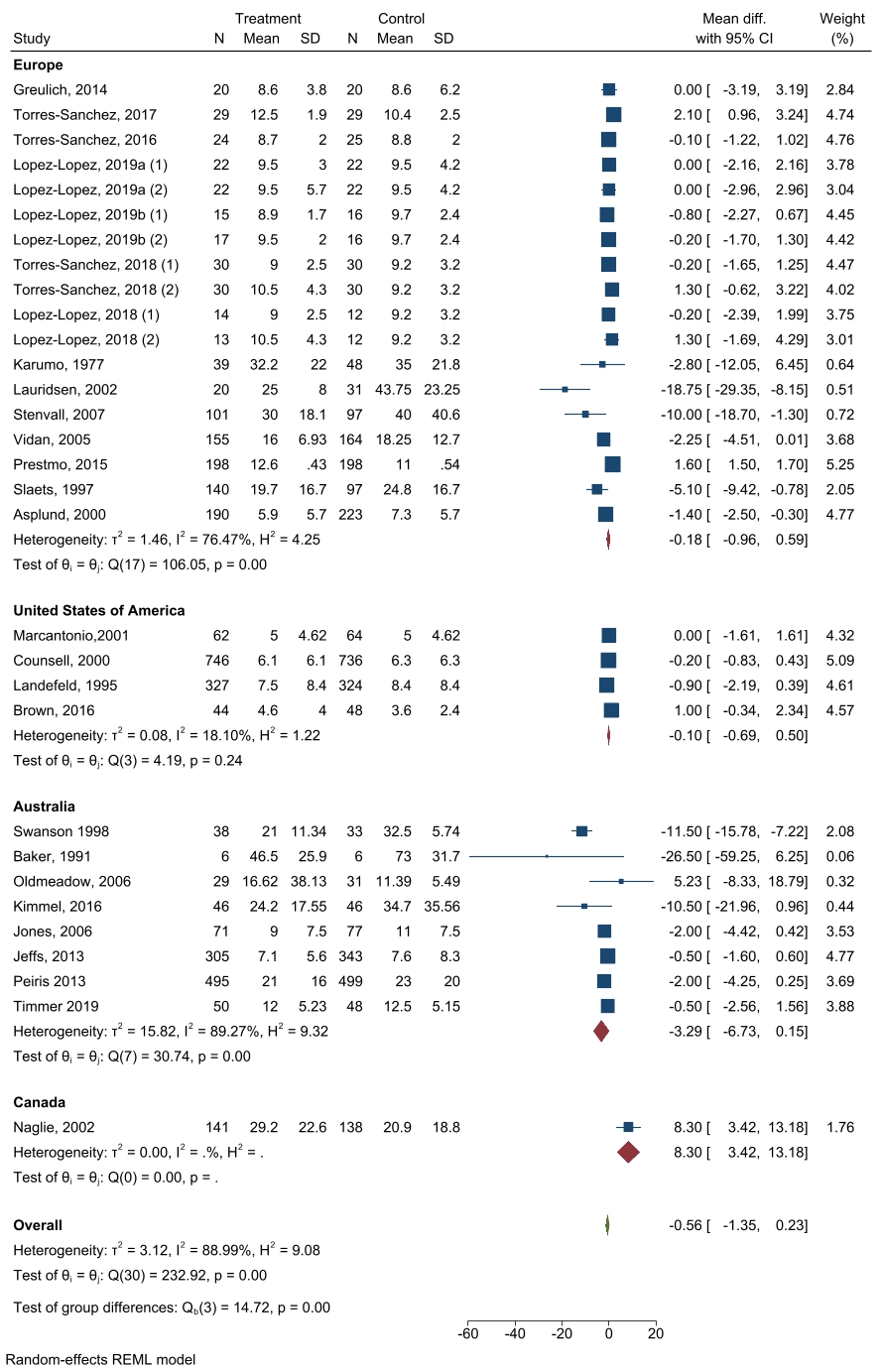


# Inpatient rehabilitation versus comparator on discharge destination of home after inpatient rehabilitation.

| Systematic review | Randomized controlled trial | Population | Intervention | Comparator |
| --- | --- | --- | --- | --- |
| Heldmann 2019 | Asplund 2000 | Medical admission | Early intervention; Discharge planning; Increased medical care | Usual care (Sweden). Physiotherapy and occupational therapy not routinely available. |
| Heldmann 2019 | Jones 2006 | Medical admission | Exercise scheduling for strengthening; Repeated practice activities (+/- increasing demands) | Usual care (Australia). Standard physiotherapy. |
| Smith 2020b | Landefeld 1995 | Medical admission | Repeated practice activities (+/- increasing demands); Antecedents; Team meetings & care planning; Discharge planning; Increased medical care; Nutritional intervention | Usual care (USA). Physiotherapy. |
| Heldmann 2019 | Naglie 2002 | Hip fracture | Repeated practice activities (+/- increasing demands); Feedback and monitoring; Shaping knowledge; Team meetings & care planning; Discharge planning; Increased medical care; Early intervention; Home visit | Usual care (Canada). Physiotherapy if referred. Occupational therapy rarely. |
| Heldmann 2019 | Oldmeadow 2006 | Hip fracture | Early intervention | Delayed ambulation to post op day 3 or 4 |
| Heldmann 2019 | Kimmel 2016 | Hip fracture | Repeated exercise rehabilitation | Usual care (Australia). Daily physiotherapy. |
| Heldmann 2019 | Barnes 2012 | Medical admission | Repeated practice activities (+/- increasing demands); Antecedents; Team meetings & care planning; Discharge planning; Increased medical care; Nutritional intervention | Usual care (USA). Physiotherapy if referred. |
| Heldmann 2019 | Prestmo 2015 | Hip fracture | Repeated practice activities (+/- increasing demands); Goals and planning; Team meetings & care planning; Discharge planning; Increased medical care; Nutritional intervention; Early intervention | Usual care (Norway). Physiotherapy according to guidelines. No occupational therapists. |
| Smith 2020 | Marcantonio 2001 | Hip fracture | Antecedents; Increased medical care; Nutritional intervention; Early intervention | Usual care (USA). Medical care. |
| Bachmann 2010 | Swanson 1998 | Hip fracture | Repeated exercise rehabilitation; Team meetings & care planning; Discharge planning; Increased medical care; Early intervention; Home visit | Usual care (Australia). Physiotherapy. Occupational therapy on referral. |
| Peck 2020 | Resnick 2016 | Orthopaedic trauma | Repeated practice activities (+/- increasing demands); Goals and planning; Feedback and monitoring; Shaping knowledge; Antecedents; Increased medical care | Usual care (USA), plus education |


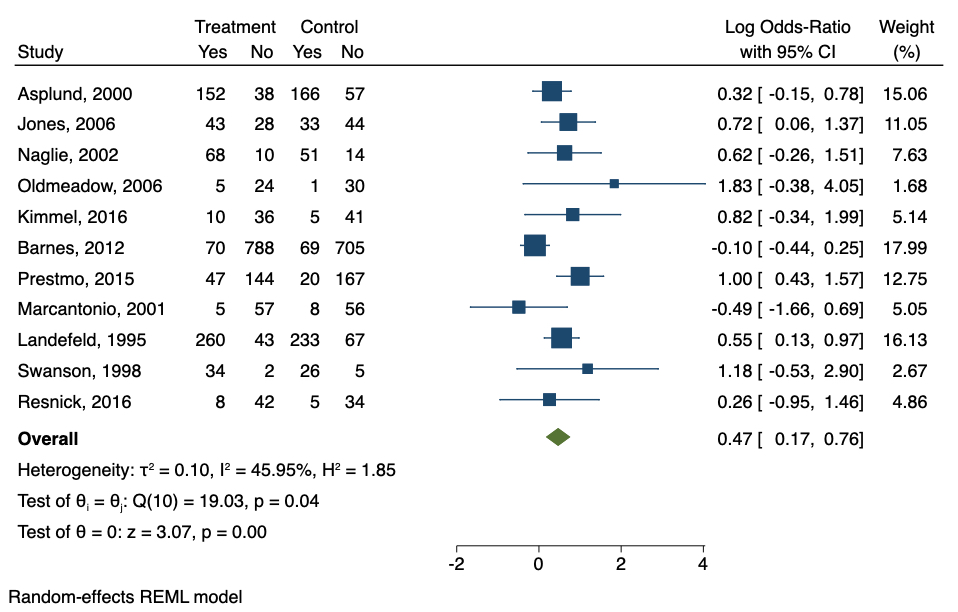


With removal of RCT from systematic review of critically low quality


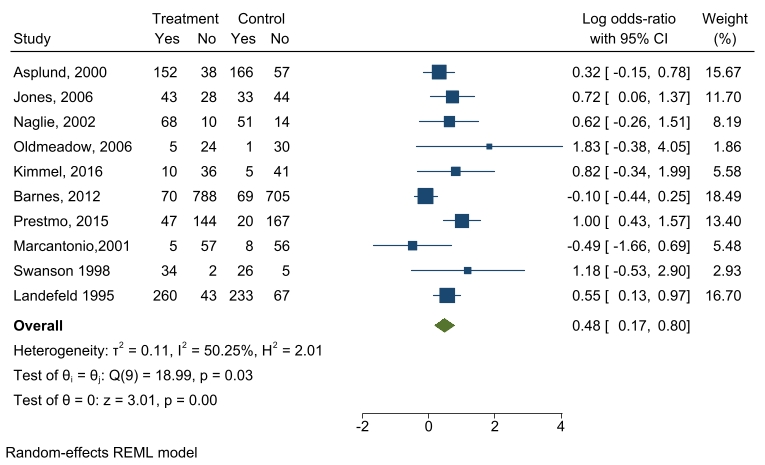


# Inpatient rehabilitation versus comparator on final destination of home

| Systematic review | Randomized controlled trial | Population | Intervention | Comparator | Length of follow up |
| --- | --- | --- | --- | --- | --- |
| Heldmann 2019 | Asplund 2000 | Medical admission | Early intervention; Discharge planning; Increased medical care | Usual care (Sweden). Physiotherapy and occupational therapy not routinely available. | 3 months |
| Heldmann 2019 | Prestmo 2015 | Hip fracture | Repeated practice activities (+/- increasing demands); Goals and planning; Team meetings & care planning; Discharge planning; Increased medical care; Nutritional intervention; Early intervention | Usual care (Norway). Physiotherapy according to guidelines. No occupational therapists. | 12 months |

# Inpatient rehabilitation versus comparator on mortality after inpatient rehabilitation.

| Systematic review | Randomized controlled trial | Population | Intervention | Comparator |
| --- | --- | --- | --- | --- |
| de Morton 2007 | Slaets 1997 | Medical admission | Repeated exercise rehabilitation; Team meetings & care planning; Increased medical care; | Usual care (The Netherlands). Services provided by physicians and nurses. |
| Heldmann 2019 | Asplund 2000 | Medical admission | Early intervention; Discharge planning; Increased medical care | Usual care (Sweden). Physiotherapy and occupational therapy not routinely available. |
| Smith 2020b | Counsell 2000 | Medical admission | Repeated practice activities (+/- increasing demands); Antecedents; Team meetings & care planning; Discharge planning; Increased medical care; Nutritional intervention | Usual care (USA). Medical care. |
| Heldmann 2019 | Jones 2006 | Medical admission | Exercise scheduling for strengthening; Repeated practice activities (+/- increasing demands) | Usual care (Australia). Standard physiotherapy. |
| Smith 2020b | Landefeld 1995 | Medical admission | Repeated practice activities (+/- increasing demands); Antecedents; Team meetings & care planning; Discharge planning; Increased medical care; Nutritional intervention | Usual care (USA). Physiotherapy. |
| Heldmann 2019 | Naglie 2002 | Hip fracture | Repeated practice activities (+/- increasing demands); Feedback and monitoring; Shaping knowledge; Team meetings & care planning; Discharge planning; Increased medical care; Early intervention; Home visit | Usual care (Canada). Physiotherapy if referred. Occupational therapy rarely. |
| Heldmann 2019 | Stenvall 2007 | Hip fracture | Endurance exercise; Repeated practice activities (+/- increasing demands); Goals and planning; Shaping knowledge; Team meetings & care planning; Increased medical care; Nutritional intervention; Early intervention | Usual care (Sweden). Exercise rehabilitation with daily physiotherapy and occupational therapy as needed. |
| Heldmann 2019 | Vidan 2005 | Hip fracture | Repeated exercise rehabilitation; Antecedents; Team meetings & care planning; Increased medical care | Usual care (Spain) Physiotherapy. Occupational therapy not available. |
| Heldmann 2019 | Oldmeadow 2006 | Hip fracture | Early intervention | Delayed ambulation to post op day 3 or 4 |
| Heldmann 2019 | Barnes 2012 | Medical admission | Repeated practice activities (+/- increasing demands); Antecedents; Team meetings & care planning; Discharge planning; Increased medical care; Nutritional intervention | Usual care (USA). Physiotherapy if referred. |
| Bachmann 2010 | Swanson 1998 | Hip fracture | Repeated exercise rehabilitation; Team meetings & care planning; Discharge planning; Increased medical care; Early intervention; Home visit | Usual care (Australia). Physiotherapy. Occupational therapy on referral. |
| Peck 2020 | Resnick 2016 | Orthopaedic trauma | Repeated practice activities (+/- increasing demands); Goals and planning; Feedback and monitoring; Shaping knowledge; Antecedents; Increased medical care | Usual care (USA), plus education |


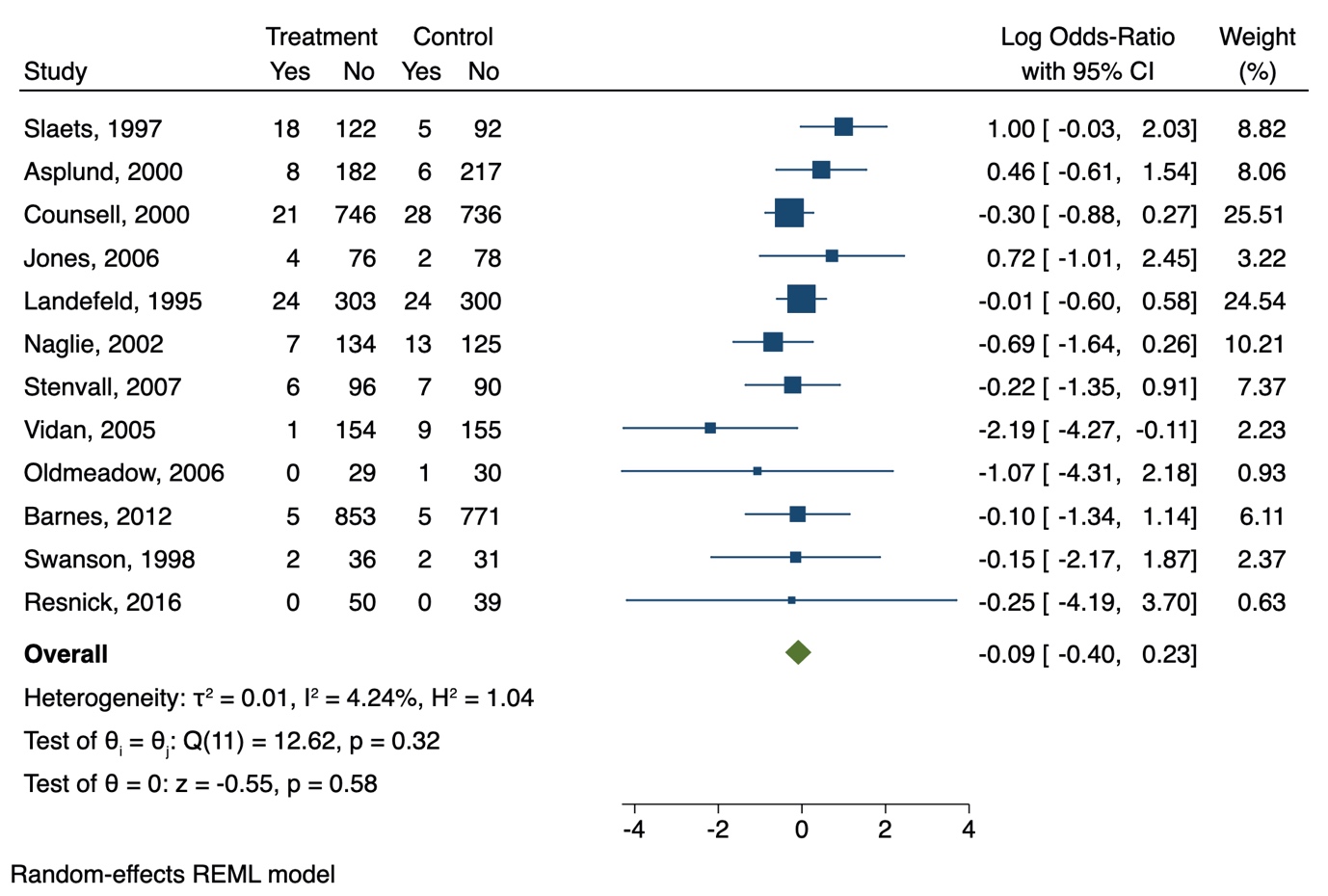


With removal of RCT from systematic review of critically low quality


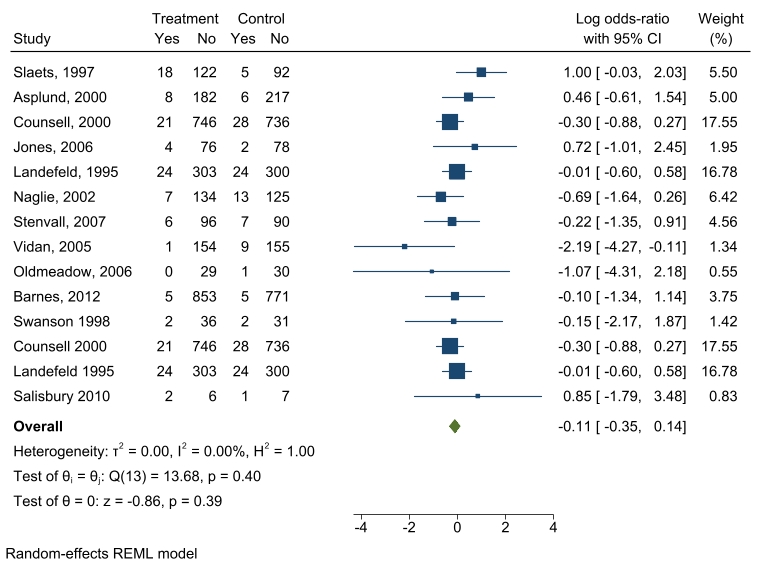


# Inpatient rehabilitation versus comparator on mortality at follow up

| Systematic review | Randomized controlled trial | Population | Intervention | Comparator | Length of follow up |
| --- | --- | --- | --- | --- | --- |
| Handoll 2011 | Graham 1968 | Hip fracture | Endurance exercise; Early intervention | Delayed weight bearing until 12 weeks after surgery | 12 months |
| Handoll 2011 | Mitchell 2001 | Hip fracture | Strengthening exercise; Repeated practice functions (+/-increasing demands) | Usual care (UK). 20 minutes physiotherapy per weekday. | 16 weeks |
| Heldmann 2019 | Asplund 2000 | Medical admission | Early intervention; Discharge planning; Increased medical care | Usual care (Sweden). Physiotherapy and occupational therapy not routinely available. | 3 months |
| Smith 2020b | Counsell 2000 | Medical admission | Repeated practice activities (+/- increasing demands); Antecedents; Team meetings & care planning; Discharge planning; Increased medical care; Nutritional intervention | Usual care (USA). Medical care. | 3 months |
| Smith 2020b | Landefeld 1995 | Medical admission | Repeated practice activities (+/- increasing demands); Antecedents; Team meetings & care planning; Discharge planning; Increased medical care; Nutritional intervention | Usual care (USA). Physiotherapy. | 3 months |
| Heldmann 2019 | Naglie 2002 | Hip fracture | Repeated practice activities (+/- increasing demands); Feedback and monitoring; Shaping knowledge; Team meetings & care planning; Discharge planning; Increased medical care; Early intervention; Home visit | Usual care (Canada). Physiotherapy if referred. Occupational therapy rarely. | 6 months |
| Heldmann 2019 | Stenvall 2007 | Hip fracture | Endurance exercise; Repeated practice activities (+/- increasing demands); Goals and planning; Shaping knowledge; Team meetings & care planning; Increased medical care; Nutritional intervention; Early intervention | Usual care (Sweden). Exercise rehabilitation with daily physiotherapy and occupational therapy as needed. | 12 months |
| Heldmann 2019 | Vidan 2005 | Hip fracture | Repeated exercise rehabilitation; Antecedents; Team meetings & care planning; Increased medical care | Usual care (Spain) Physiotherapy. Occupational therapy not available. | 12 months |
| Martinez-Velilla, 2016 | Saltvedt 2002 | Medical admission | Shaping knowledge; Antecedents; Team meetings & care planning; Discharge planning; Increased medical care; Early intervention; Home visit | Usual care (Norway), Allied health if referred. | 12 months |
| Smith 2020b | Timmer 2019 | Medical A  admission | Repeated practice activities (+/- increasing demands); Goals and planning; Feedback and monitoring; Social support; Shaping knowledge; Natural consequences | Usual care (Australia) plus a brief activity pacing education. Physiotherapy and occupational therapy. | 3 months |
| Bachmann 2010 | Swanson 1998 | Hip fracture | Repeated exercise rehabilitation; Team meetings & care planning; Discharge planning; Increased medical care; Early intervention; Home visit | Usual care (Australia). Physiotherapy. Occupational therapy on referral. | 6 months |
| Peck 2020 | Resnick 2016 | Orthopaedic trauma | Repeated practice activities (+/- increasing demands); Goals and planning; Feedback and monitoring; Shaping knowledge; Antecedents; Increased medical care | Usual care (USA), plus education | 1 month |


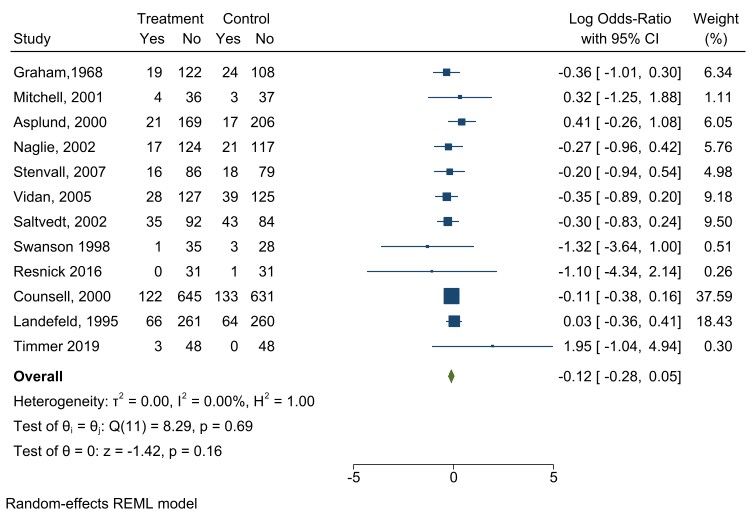


With removal of RCT from systematic review of critically low quality


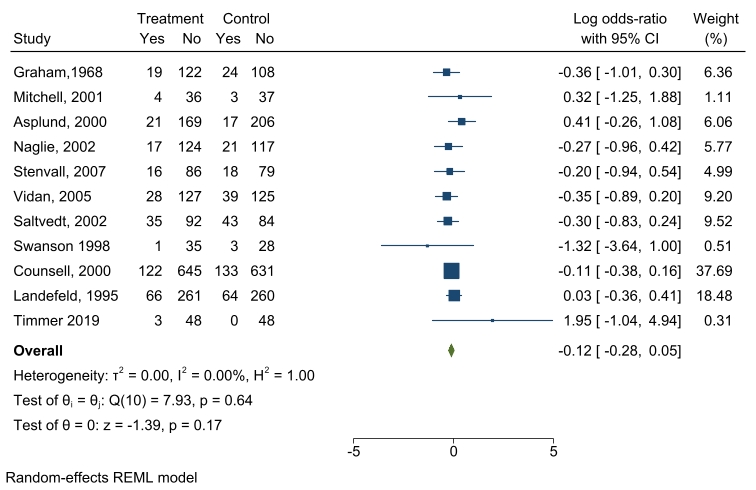

Supplement: Supplementary file 5 — Additional file 5: Supplementary File 5. Meta-analyses results. Results of meta-analyses (forest plot) of randomized controlled trials identified from systematic reviews included in this overview review for functioning, quality of life, length of stay, discharge destination and mortality. Each meta-analysis is accompanied by a table which describes the characteristics of each randomized controlled trial included in each meta-analysis. [file 12877_2022_3169_MOESM5_ESM.docx]
