# Supplementary material for: Effect of inpatient rehabilitation treatment ingredients on functioning, quality of life, length of stay, discharge destination, and mortality among older adults with unplanned admission: an overview review
Source: BMC Geriatr. 2022 Jun 11;22:501. doi: 10.1186/s12877-022-03169-2 (PMC9188066; doi:10.1186/s12877-022-03169-2)
Supplement: Supplementary file 6 — Additional file 6: Supplementary File 6. Treatment ingredient meta-analyses results. Results of meta-analyses (forest plot) of randomized controlled trials identified from systematic reviews included in this overview review by treatment ingredient. [file 12877_2022_3169_MOESM6_ESM.docx]

SUPPLEMENTARY FILE 6

# List of meta-analyses

1. Inpatient rehabilitation vs. comparator on activities of daily living after inpatient rehabilitation.
2. Subgroup with ‘goals and planning’
3. Subgroup with ‘feedback and monitoring’
4. Subgroup with ‘repeated practice activities’
5. Subgroup with ‘endurance exercise’
6. Subgroup with ‘shaping knowledge’
7. Subgroup with ‘increased medical care’
8. Subgroup with ‘strengthening exercise’
9. Subgroup with ‘energy applied to soft tissue’
10. Subgroup with ‘repeated exercise rehabilitation’
11. Subgroup with ‘nutritional intervention’
12. Subgroup with ‘natural consequences’, ‘social support’
13. Subgroup with ‘antecedents’
14. Subgroup with ‘early intervention’
15. Inpatient rehabilitation vs. comparator on walking speed at end of intervention
    1. Subgroup with ‘strengthening exercise’
    2. Subgroup with ‘goals and planning’
    3. Subgroup with ‘repeated exercise rehabilitation’
16. Inpatient rehabilitation vs. comparator on walking endurance at end of intervention
    1. Subgroup with ‘endurance exercise’
    2. Subgroup with ‘energy applied to soft tissue’
    3. Subgroup with ‘shaping knowledge’
    4. Subgroup with ‘early intervention’
    5. Subgroup with ‘strengthening exercise’
    6. Subgroup with ‘goals and planning’
17. Inpatient rehabilitation vs. comparator on walking endurance pre/post intervention
18. Subgroup with ‘endurance exercise’
19. Inpatient rehabilitation vs. comparator on discharge destination of home at intervention end (overall and by diagnosis).
20. Subgroup with ‘increased medical care’
21. Subgroup with ‘early intervention’
22. Subgroup with ‘repeated practice activities (+/- increasing demands)’
23. Subgroup with ‘team meetings & care planning’
24. Subgroup with ‘discharge planning’
25. Subgroup with ‘antecedents’
26. Subgroup with ‘nutritional intervention’
27. Subgroup with ‘goals and planning’
28. Subgroup with ‘repeated exercise rehabilitation’

# INPATIENT REHABILITATION VERSUS COMPARATOR ON ACTIVITIES OF DAILY LIVING after inpatient rehabilitation FOR SUBGROUP WITH ‘GOALS AND PLANNING’

# INPATIENT REHABILITATION VERSUS COMPARATOR ON ACTIVITIES OF DAILY LIVING after inpatient rehabilitation FOR SUBGROUP WITH ‘FEEDBACK AND MONITORING’

# INPATIENT REHABILITATION VERSUS COMPARATOR ON ACTIVITIES OF DAILY LIVING after inpatient rehabilitation FOR SUBGROUP WITH ‘REPEATED PRACTICE ACTIVITIES’

# Inpatient rehabilitation versus comparator on activities of daily living after inpatient rehabilitation for subgroup with ‘endurance EXERCISE’

# INPATIENT REHABILITATION VERSUS COMPARATOR ON ACTIVITIES OF DAILY LIVING after inpatient rehabilitation FOR SUBGROUP WITH ‘SHAPING KNOWLEDGE’

# INPATIENT REHABILITATION VERSUS COMPARATOR ON ACTIVITIES OF DAILY LIVING after inpatient rehabilitation FOR SUBGROUP WITH ‘INCREASED MEDICAL CARE’

# Inpatient rehabilitation versus comparator on activities of daily living after inpatient rehabilitation for subgroup with ‘strengthening exercise’

# INPATIENT REHABILITATION VERSUS COMPARATOR ON ACTIVITIES OF DAILY LIVING after inpatient rehabilitation FOR SUBGROUP WITH ‘ENERGY APPLIED TO SOFT TISSUE’

# INPATIENT REHABILITATION VERSUS COMPARATOR ON ACTIVITIES OF DAILY LIVING after inpatient rehabilitation FOR SUBGROUP WITH ‘REPEATED EXERCISE REHABILITATION’

# INPATIENT REHABILITATION VERSUS COMPARATOR ON ACTIVITIES OF DAILY LIVING after inpatient rehabilitation FOR SUBGROUP WITH ‘NUTRITIONAL INTERVENTION’

# INPATIENT REHABILITATION VERSUS COMPARATOR ON ACTIVITIES OF DAILY LIVING after inpatient rehabilitation FOR SUBGROUP WITH ‘ANTECEDENTS’

# INPATIENT REHABILITATION VERSUS COMPARATOR ON ACTIVITIES OF DAILY LIVING after inpatient rehabilitation FOR SUBGROUP WITH ‘EARLY INTERVENTION’

# Inpatient rehabilitation versus comparator on walking speed after inpatient rehabilitation for subgroup with ‘strengthening Exercise’


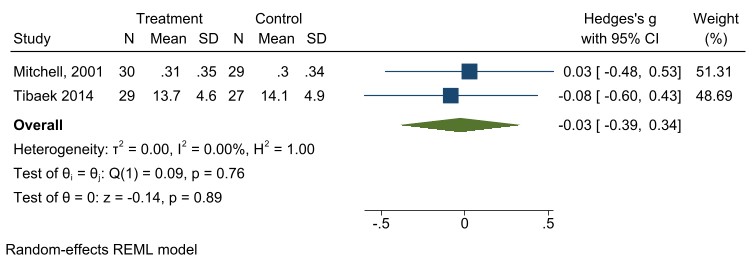


# INPATIENT REHABILITATION VERSUS COMPARATOR ON WALKING SPEED after inpatient rehabilitation FOR SUBGROUP WITH ‘REPEATED EXERCISE REHABILITATION’

#

# INPATIENT REHABILITATION VERSUS COMPARATOR ON WALKING ENDURANCE after inpatient rehabilitation FOR SUBGROUP WITH ‘ENDURANCE EXERCISE’

# INPATIENT REHABILITATION VERSUS COMPARATOR ON WALKING ENDURANCE after inpatient rehabilitation FOR SUBGROUP WITH ‘SHAPING KNOWLEDGE’


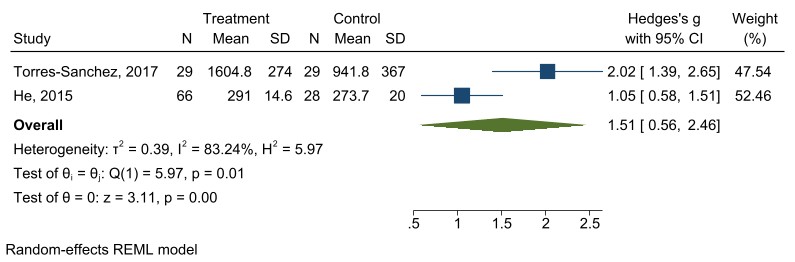


# Inpatient rehabilitation versus comparator on walking endurance after inpatient rehabilitation for subgroup with ‘Early intervention’

# Inpatient rehabilitation versus comparator on walking endurance pre/post intervention for subgroup with ‘ENDURANCE EXERCISE’

# INPATIENT REHABILITATION VERSUS COMPARATOR ON DISCHARGE DESTINATION OF HOME after inpatient rehabilitation FOR SUBGROUP WITH ‘INCREASED MEDICAL CARE’

# INPATIENT REHABILITATION VERSUS COMPARATOR ON DISCHARGE DESTINATION OF HOME after inpatient rehabilitation FOR SUBGROUP WITH ‘EARLY INTERVENTION’

# Inpatient rehabilitation versus comparator on discharge destination of home after inpatient rehabilitation for subgroup with ‘Repeated practice activities’

# INPATIENT REHABILITATION VERSUS COMPARATOR ON DISCHARGE DESTINATION OF HOME after inpatient rehabilitation FOR SUBGROUP WITH ‘TEAM MEETING AND CARE PLANNING’

# INPATIENT REHABILITATION VERSUS COMPARATOR ON DISCHARGE DESTINATION OF HOME after inpatient rehabilitation FOR SUBGROUP WITH ‘DISCHARGE PLANNING’

# INPATIENT REHABILITATION VERSUS COMPARATOR ON DISCHARGE DESTINATION OF HOME after inpatient rehabilitation FOR SUBGROUP WITH ‘ANTECEDENTS’

# INPATIENT REHABILITATION VERSUS COMPARATOR ON DISCHARGE DESTINATION OF HOME after inpatient rehabilitation FOR SUBGROUP WITH ‘NUTRITIONAL INTERVENTION’

# INPATIENT REHABILITATION VERSUS COMPARATOR ON DISCHARGE DESTINATION OF HOME after inpatient rehabilitation FOR SUBGROUP WITH ‘GOALS AND PLANNING’

# INPATIENT REHABILITATION VERSUS COMPARATOR ON DISCHARGE DESTINATION OF HOME after inpatient rehabilitation FOR SUBGROUP WITH ‘REPEATED EXERCISE REHABILITATION’
