# Supplementary material for: Effect of inpatient rehabilitation treatment ingredients on functioning, quality of life, length of stay, discharge destination, and mortality among older adults with unplanned admission: an overview review
Source: BMC Geriatr. 2022 Jun 11;22:501. doi: 10.1186/s12877-022-03169-2 (PMC9188066; doi:10.1186/s12877-022-03169-2)
Supplement: Supplementary file 7 — Additional file 7: Supplementary File 7. Narrative results. Results of randomized controlled trials identified from systematic reviews included in this overview review which were not incorporated into the meta-analyses and reasons why they were not incorporated. [file 12877_2022_3169_MOESM7_ESM.docx]

Supplementary File 7: Summary of randomized controlled trials/outcomes not included in meta-analyses.

| **Reason not in MA** | **Review author year** | **RCT author year** | **Outcome** | **Result** |
| --- | --- | --- | --- | --- |
| RCT in MA with different measure | Heldmann 2019 | Kimmel 2016 | modified Iowa Level of Assistance score intervention end | No between group difference. Between group difference favouring intervention when controlling for confounders. |
| no measure of dispersion | Heldmann 2019 | Blanc-Bisson 2008 | Course of ADL (Katz Index) between intervention end and follow up. | No between group difference. |
| sole study | Heldmann 2019 | Jones 2006 | change in Barthel Index at intervention end stratified by Barthel Index at admission | No between group difference. |
| sole study | Heldmann 2019 | Jones 2006 | change in TUG | No between group difference. |
| RCT in MA with different measure | Heldmann 2019 | Prestmo 2015 | Short Physical Performance Battery at intervention end | No between group difference. |
| RCT in MA with different measure | Heldmann 2019 | Prestmo 2015 | Short Physical Performance Battery at follow up (12 months) | Between group difference favouring intervention: 0.69 (95% CI: 0.1, 1.28) p = 0.023 |
| no measure of central tendency/dispersion | Heldmann 2019 | Hagsten 2004 | Modified Klein-Bell ADL Scale at follow up (2 months) | Between group differences: moving around indoors (p = 0.03), Performance of light housework (p = 0.05), and getting in and out of a car (p = 0.05) |
| RCT in MA with different measure | Handoll2011 | Mitchell 2001 | TUG at intervention end | No between group difference. |
| RCT in MA with different measure | Handoll2011 | Mitchell 2001 | TUG at follow up (16 weeks) | No between group difference. |
| sole study | Handoll2011 | Mitchell 2001 | gait speed- (metres/second) at follow up (16 weeks) | No between group difference. |
| no measure of central tendency/dispersion | Handoll2011 | Karumo 1977 | Mortality at follow-up (9 weeks) | No between group difference. |
| RCT in MA with different measure | Machado 2020 | Lopez-Lopez 2019a (1) | London chest ADL score at intervention end | No between group difference. |
| RCT in MA with different measure | Machado 2020 | Lopez-Lopez 2019a (2) | London chest ADL score at intervention end | No between group difference. |
| no measure of central tendency/dispersion | Machado 2020 | Liao 2015 | 6MWD at intervention end | Between group difference favouring intervention p <0.05. |
| no measure of central tendency/dispersion | Machado 2020 | Nava 1998 | 6MWD at intervention end | Between group difference favouring intervention p < 0.001. |
| no measure of central tendency/dispersion | Machado 2020 | Borges 2014 | Saint George’s Respiratory Questionnaire at follow up (1 month) | Between group difference favouring intervention for impact domain p < 0.05. No between group difference for activity domain. |
| no measure of central tendency/dispersion | Machado 2020 | Borges 2014 | 6MWD at follow up (30 days) | Within group differences: control p > 0.05. intervention p < 0.05. |
| no measure of central tendency/dispersion | Machado 2020 | He 2015 | CRQ-SAS score at intervention end. | Within group differences: control ‘slight decline’. intervention p <0.001. |
| no measure of central tendency/dispersion | Martinez Vellila 2016 | Saltvedt 2002 | TUG at intervention end | No between group difference. |
| no measure of central tendency/dispersion | Martinez Vellila 2016 | Saltvedt 2002 | Barthel Index at follow up (12 months) | No between group difference. |
| RCT in MA with different measure | Peiris 2018 | Peiris 2013 | EuroQoL visual analogue scale at intervention end | No between group difference. |
| RCT in MA with different measure | Peiris 2018 | Peiris 2013 | EuroQoL visual analogue scale (12 months) | No between group difference. |
| RCT in MA with different measure | Peiris 2018 | Peiris 2013 | Personal Care Participation Assessment and Resource Tool at intervention end | No between group difference. |
| sole study | Peiris 2018 | Peiris 2013 | EuroQoL questionnaire 5D (minimally clinically important difference) at intervention end | Between group difference favouring intervention (RR = 1.18 95% CI 1.04 – 1.34) |
| sole study | Peiris 2018 | Peiris 2013 | EuroQoL questionnaire 5D (minimally clinically important difference) (12 months) | No between group difference. |
| sole study | Scrivener 2015 | Said 2012 | change in DEMMI at intervention end | No between group difference. |
| RCT in MA with different measure | Smith, 2020b | Counsell 2000 | Physical Performance and Mobility Examination at intervention end | Between group difference favouring intervention (mean difference 0.63, 95% CI 0.09 - 1.17) p = 0.027) |
| RCT in MA with different measure | Smith, 2020b | Timmer 2019 | AusTOMs-OT Impairment-Scale 4 at intervention end | No between group difference. |
| RCT in MA with different measure | Smith, 2020b | Timmer 2019 | AusTOMs-OT Impairment-Scale 4 (3 months) | No between group difference. |
| RCT in MA with different measure | Smith, 2020b | Timmer 2019 | AusTOMs-OT Impairment-Scale 7 at intervention end | No between group difference. |
| RCT in MA with different measure | Smith, 2020b | Timmer 2019 | AusTOMs-OT Impairment-Scale 7 (3 months) | No between group difference. |
| RCT in MA with different measure | Smith, 2020b | Timmer 2019 | AusTOMs-OT Impairment-Scale 8 at intervention end | No between group difference. |
| RCT in MA with different measure | Smith, 2020b | Timmer 2019 | AusTOMs-OT Impairment-Scale 8 (3 months) | No between group difference. |
| RCT in MA with different measure | Smith, 2020b | Timmer 2019 | AusTOMs-OT Activity limitation-scale 4 at intervention end | No between group difference. |
| RCT in MA with different measure | Smith, 2020b | Timmer 2019 | AusTOMs-OT Activity limitation-scale 4 (3 months) | No between group difference. |
| RCT in MA with different measure | Smith, 2020b | Timmer 2019 | AusTOMs-OT Activity limitation-scale 7 at intervention end | No between group difference. |
| RCT in MA with different measure | Smith, 2020b | Timmer 2019 | AusTOMs-OT Activity limitation-scale 7 (3 months) | No between group difference. |
| RCT in MA with different measure | Smith, 2020b | Timmer 2019 | AusTOMs-OT Activity limitation-scale 8 at intervention end | No between group difference. |
| RCT in MA with different measure | Smith, 2020b | Timmer 2019 | AusTOMs-OT Activity limitation-scale 8 (3 months) | No between group difference. |
| RCT in MA with different measure | Yasmeen, 2020 | Louie 2012 | Lawton Instrumental ADL scale at intervention end | Within group difference pre and post programme in both control (p<0.01) and intervention (p<0.01). No between group difference. |

MA = meta-analysis; RCT= randomized controlled trial; ADL = activities of daily living; eq-5d = EuroQol 5 dimensions; 6mwd = 6 minute walk distance; TUG = timed up and go; CRQ-SAS score = Chronic Respiratory Disease Questionnaire Self-Administered Standardized; DEMMI = de Morton Mobility Index. AusTOMs-OT = Australian Therapy Outcome Measures-Occupational Therapy
